# Transcriptomic survey of key reproductive and metabolic tissues in mouse models of polycystic ovary syndrome

**DOI:** 10.1038/s42003-022-04362-0

**Published:** 2023-01-18

**Authors:** Yu Pei, Sanjiv Risal, Hong Jiang, Haojiang Lu, Eva Lindgren, Elisabet Stener-Victorin, Qiaolin Deng

**Affiliations:** 1 Department of Physiology and Pharmacology, Karolinska Institutet, Stockholm, Sweden; 2 Center for molecular medicine, Karolinska University Hospital, Stockholm, Sweden

**Keywords:** Transcriptomics, Physiology

## Abstract

Excessive androgen production and obesity are key to polycystic ovary syndrome (PCOS) pathogenesis. Prenatal androgenized (PNA), peripubertal androgenized, and overexpression of nerve growth factor in theca cells (17NF) are commonly used PCOS-like mouse models and diet-induced maternal obesity model is often included for comparsion. To reveal the molecular features of these models, we have performed transcriptome survey of the hypothalamus, adipose tissue, ovary and metaphase II (MII) oocytes. The largest number of differentially expressed genes (DEGs) is found in the ovaries of 17NF and in the adipose tissues of peripubertal androgenized models. In contrast, hypothalamus is most affected in PNA and maternal obesity models suggesting fetal programming effects. The *Ms4a6e* gene, membrane-spanning 4-domains subfamily A member 6E, a DEG identified in the adipose tissue in all mouse models is also differently expressed in adipose tissue of women with PCOS, highlighting a conserved disease function. Our comprehensive transcriptomic profiling of key target tissues involved in PCOS pathology highlights the effects of developmental windows for androgen exposure and maternal obesity, and provides unique resource to investigate molecular mechanisms underlying PCOS pathogenesis.

## Abstract (web summary)

Transcriptomic mapping of key reproductive and metabolic tissues and oocytes in mouse models of polycystic ovary syndrome reveals that the hypothalamus is susceptible to fetal programming while adipose and ovary are more affected upon postnatal androgen exposure.

## Introduction

PCOS affects ~15% of women in their reproductive age and the key feature of the syndrome is hyperandrogenism leading to abnormal follicular development, infertility, and an increased risk of type 2 diabetes^1–5^. Genetic factors account for <10% of the heritability^1,6–10^ and the etiology of PCOS is considered as an interplay of genetic, epigenetic, and developmental factors^11–15^. To understand the complex pathophysiology of PCOS, >30 PCOS-like animal models have been developed, among which rodent models are the most common. Rodents and humans share evolutionarily conserved similarities in the regulation of reproductive function by the hypothalamic-pituitary-gonad (HPG) axis and ovarian folliculogenesis^16^. Additionally, rodent models of PCOS can model many characteristics of the human disorder including hyperandrogenism, elevated LH, disrupted cyclicity, presence of follicular cysts/polycystic ovaries, and altered insulin sensitivity^1^.

Hyperandrogenism plays a key role in the pathogenesis of PCOS and several PCOS-like mouse models have applied excessive androgen at different developmental time windows including the prenatal androgenization (PNA) and the peripubertal androgenization models together with the transgenic 17NF model overexpressing nerve growth factor (NGF) in the ovarian theca cells^13,17–21^. These models differ in terms of the timing, dose, and approach of androgen exposure, which may result in different effects in downstream pathogenesis. The PNA mouse model is established by dihydrotestosterone (DHT) exposure to the pregnant dam at gestational days 16.5–18.5 and the female first-generation offspring exhibits disturbed estrous cycle and metabolic alterations in adulthood^19,22,23^. The peripubertal androgenized mouse model induced by continuous DHT exposure from 4 weeks of age through a slow-releasing DHT pellet or silastic tube implanted subcutaneously displays robust reproductive and cardiometabolic features reflecting PCOS symptoms^17,24,25^. 17NF model overexpresses NGF in theca cells driven by 17α-hydroxylase promoter leading to ovarian hyperandrogenism^18,20^, which reflects women with PCOS having a twofold increase in NGF in the ovarian follicular fluid^18^ indicating the role of NGF in the PCOS pathology. Excess ovarian NGF in mice, causes irregular cyclicity, subfertility, enhanced ovarian sex steroid production, and enhanced granulosa cell apoptosis^18^. Moreover, 17NF mice also display metabolic dysfunction as reflected by impaired glucose metabolism and aberrant adipose tissue morphology and function, and hepatic steatosis^18–20^ mirroring the PCOS pathophysiology. Together, these three PCOS-like mouse models are exposed to androgen at different developmental windows and serve our goal to delineate the shared and different molecular pathways/mechanisms involved in their contribution to reproductive and metabolic phenotypes of PCOS regulated by the HPG axis.

Obesity is also tightly linked with PCOS, which is one of the important factors contributing to PCOS development. A large-scale genome-wide meta-analysis of women with PCOS demonstrates that there is a shared genetic architecture between metabolic traits, including obesity and PCOS^6,26^. Obesity is also a common feature in the PNA, peripubertal androgenized, and 17NF mouse models and there is evidence showing that maternal obesity also affects germ cells and their female offspring^13,27,28^. Therefore, we also include the maternal obesity model to compare the effects of diet-induced obesity with that of androgen-induced programming since not much is known about the common and unique molecular features in their target tissues and MII oocytes in first-generation female offspring.

In three PCOS-like mouse models as well as in the diet-induced maternal obesity model, we, therefore, performed single-cell RNA-seq of MII oocytes and bulk mRNA-seq of key tissues (hypothalamus, subcutaneous adipose tissue, and ovary) to characterize androgen- and obesity-specific molecular effects. Our findings reveal that the hypothalamus is susceptible to fetal programming while adipose and ovary tissues are greatly affected after exposure around the pubertal stage. Meanwhile, MII oocytes are differentially affected by crosstalking with ovarian somatic tissue.

## Results

### Phenotypic features and experimental outline

**Fig. 1:**
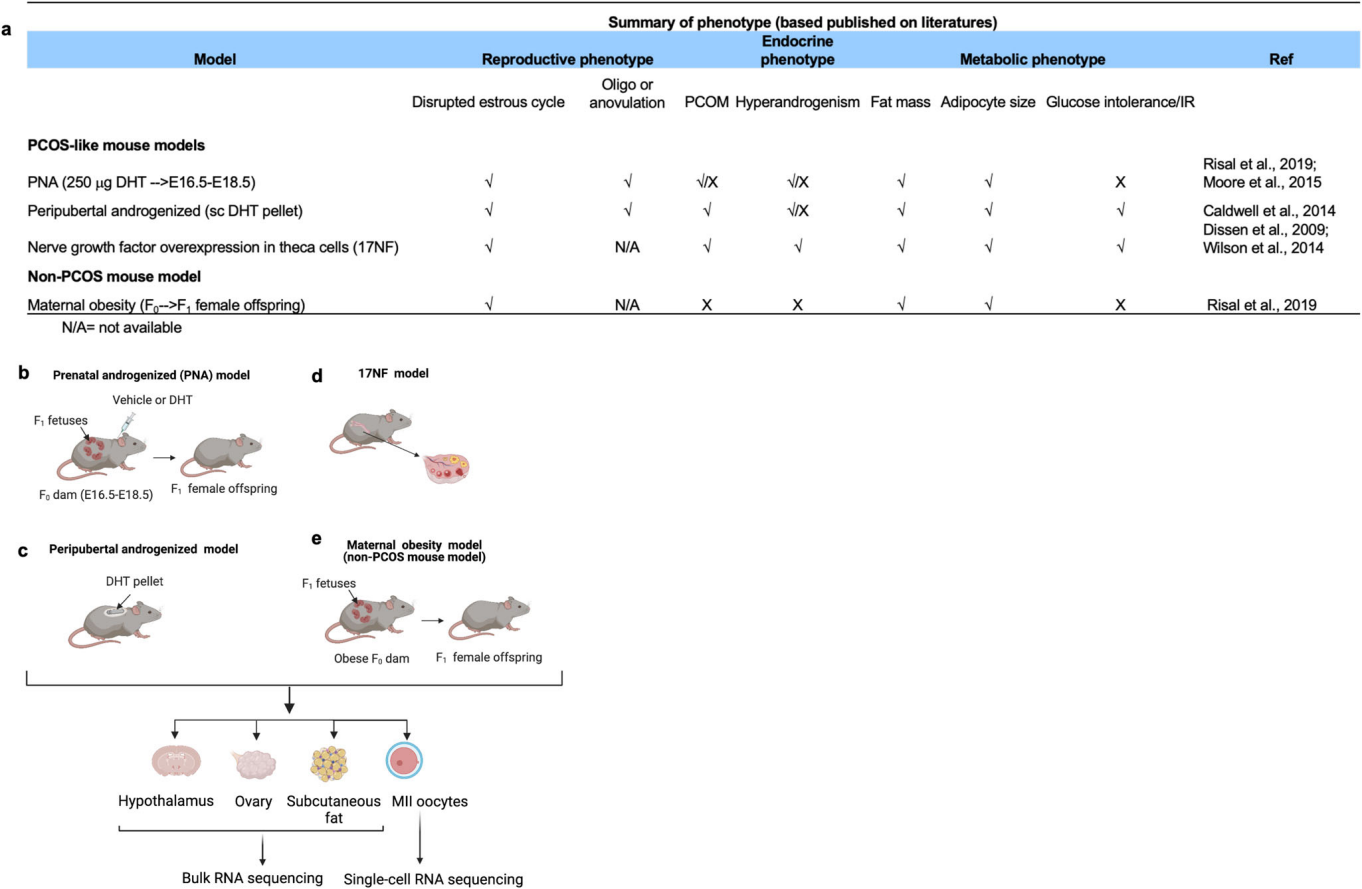
Summary of metabolic and reproductive phenotype and Illustration of three PCOS-like mouse models and a maternal high-fat high sugar (HFHS)-treated mouse model for comparative transcriptomic analyses of collected target tissues and MII oocytes. **a** Summary of metabolic and reproductive phenotype of Prenatal androgenized (PNA), Peripubertal androgenized, 17NF, and maternal obesity. **b** PNA in which F1 female offspring was analyzed. **c** Peripubertal androgenized (subcutaneous DHT implant), and **d** 17NF model: theca-cell specific nerve growth factor (NGF) overexpressing transgenic mouse models. **e** F_1_ female offspring of HFHS diet-induced maternal obese model. Different targeted tissues, namely hypothalamus, pituitary, ovaries, and subcutaneous adipose tissue are used for transcriptomic analysis by bulk mRNA sequencing. Metaphase II (MII) oocytes are used for single-cell RNA sequencing. N/A = not available.

The phenotypic features of the PNA and maternal obesity, the peripubertal androgenization, and 17NF mouse models are summarized in Fig. 1a^13,17–21^. To understand and define the common and distinct molecular signatures of key target tissues in these mouse models, we carried out bulk mRNA-seq of the hypothalamus, subcutaneous adipose tissue, and ovary as well as single-cell mRNA-seq of MII oocytes using Smart-seq2 (Fig. 1b-e).

### Distinct transcriptomic profile of hypothalamus by androgen exposure at different developmental windows

**Fig. 2:**
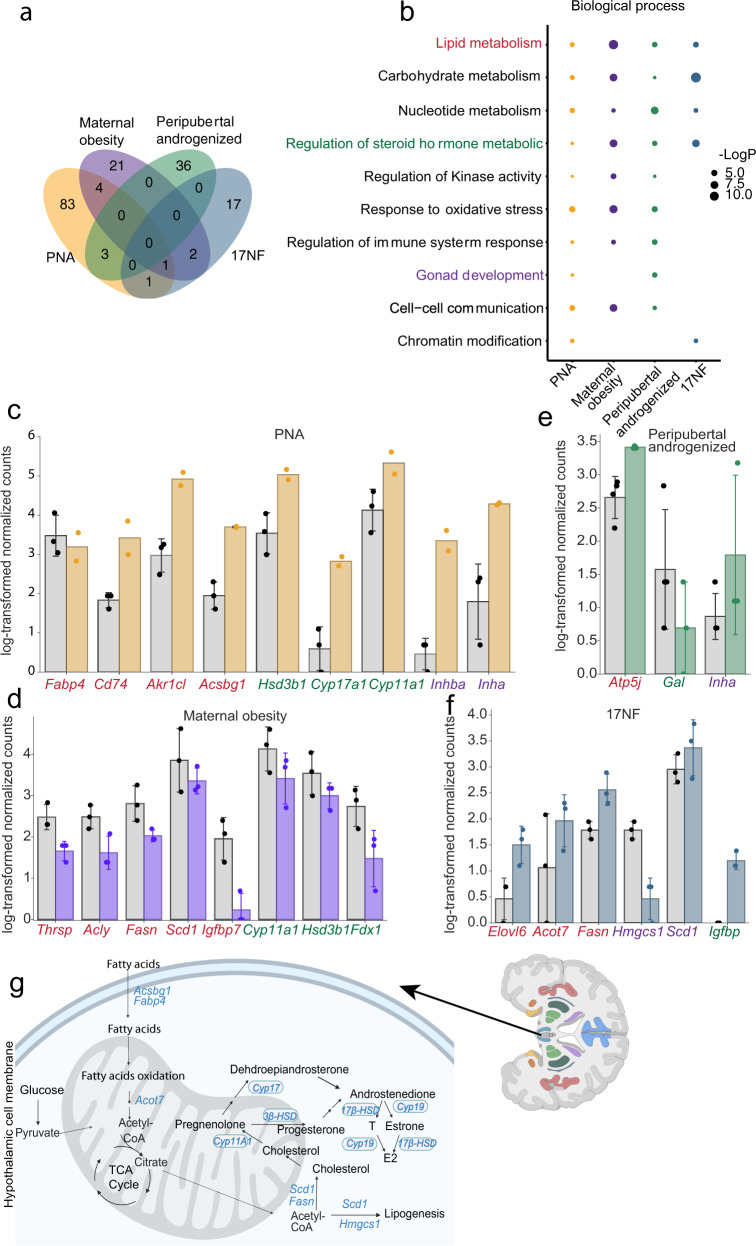
Common and distinct transcriptomic signature of the hypothalamus. **a** Venn diagram of DEGs across all animal models (PNA model, *n* = 3 in control + vehicle, *n* = 2 in prenatal DHT; maternal obesity, *n* = 3 in control, *n* = 3 in maternal obesity; Peripubertal androgenized model, *n* = 4 in control + vehicle, *n* = 3 in Peripubertal DHT; 17NF, *n* = 3 in control, *n* = 3 in 17NF). **b** Comparison of enriched gene ontology terms of DEGs across all animal models. **c**–**f** Bar plot showing expression level of DEGs enriched in lipid metabolism, steroid hormone metabolism, and gonad development in PNA, Peripubertal androgenized, 17NF, and maternal obesity, respectively. **g** Illustration of DEGs involved in lipid and steroid metabolism pathways in hypothalamus (Created with BioRender.com). Data are presented as mean ± SD. *n* number of animals.

Differentially expressed genes (DEGs) in the hypothalamus within PNA, peripubertal androgenized, and 17NF models together with fetal exposure to maternal obesity are given in Table S1. There are five overlapping DEGs between the PNA and maternal obesity mouse models: *Cfd*, *Cyp11a1*, *Fabp4*, and *Hsd3b1*, in addition to *Scd1* which is also differently expressed in the 17NF model (Fig. 2a). There are no common DEGs in the peripubertal androgenized and the 17NF mouse models (Fig. 2a). Moreover, there are three common DEGs, *Nudt3, Inha*, and *Mapk8ip3* between PNA and the peripubertal androgenized model and one additional DEG (*Rps25*) between PNA and 17NF models (Fig. 2a). To further annotate the function of identified DEGs in the hypothalamus across these four animal models, we performed GO enrichment biological processes analysis. Interestingly, all animal models showed enriched pathways related to lipid, nucleic acids, carbohydrate, nucleotide, and steroid hormone metabolism, but these pathways were regulated by different set of genes (Fig. 2b). In addition, gonad development GO terms are found in the PNA and the peripubertal androgenized mice which likely reflects the unique effect of systematic androgen exposure on the hypothalamus-ovary axis related to reproduction dysfunction (Fig. 2b). Notably, common biological pathways were found between the PNA, maternal obesity, and peripubertal androgenized models whereas the 17NF model deviated, suggesting different molecular mechanisms. All DEGs involved in lipid metabolism, steroid metabolism, and gonad development biological processes were further highlighted in their gene expression in each model with a color corresponding to each pathway highlighted (Fig. 2c–f, Table S2, Supplementary Data 1). Most genes in these pathways were upregulated in the PNA model whereas most genes were downregulated in maternal obesity, indicating opposite effect on the hypothalamus in the fetal programming by androgen and diet, respectively on DEGs (Fig. 2c, d). Notably, DEGs are different in each model although involved in the same biological processes. For example, in the hypothalamus of the PNA mouse model, *Acsbg1*, *Cd74*, *Akr1cl*, *Fabp4*, and *Scd1* are DEGs involved in fatty acid biosynthesis and steroid metabolism (Fig. 2c) whereas in the peripubertal androgenized model are *Atp5j*, *Gal*, and *Inha* differentially expressed in the same pathways (Fig. 2e). In the 17NF hypothalamus, a panel of DEGs, e.g., *Acot7, Elovl6, Fasn, Hmgcs1*, and *Scd1* (Fig. 2f) are involved in triglycerides and cholesterol metabolism. The implicated function of selected DEGs has been further illustrated in the lipid and steroid metabolism pathways (Fig. 2g).

### Common PCOS-related ovarian cellular processes revealed in different mouse models

**Fig. 3:**
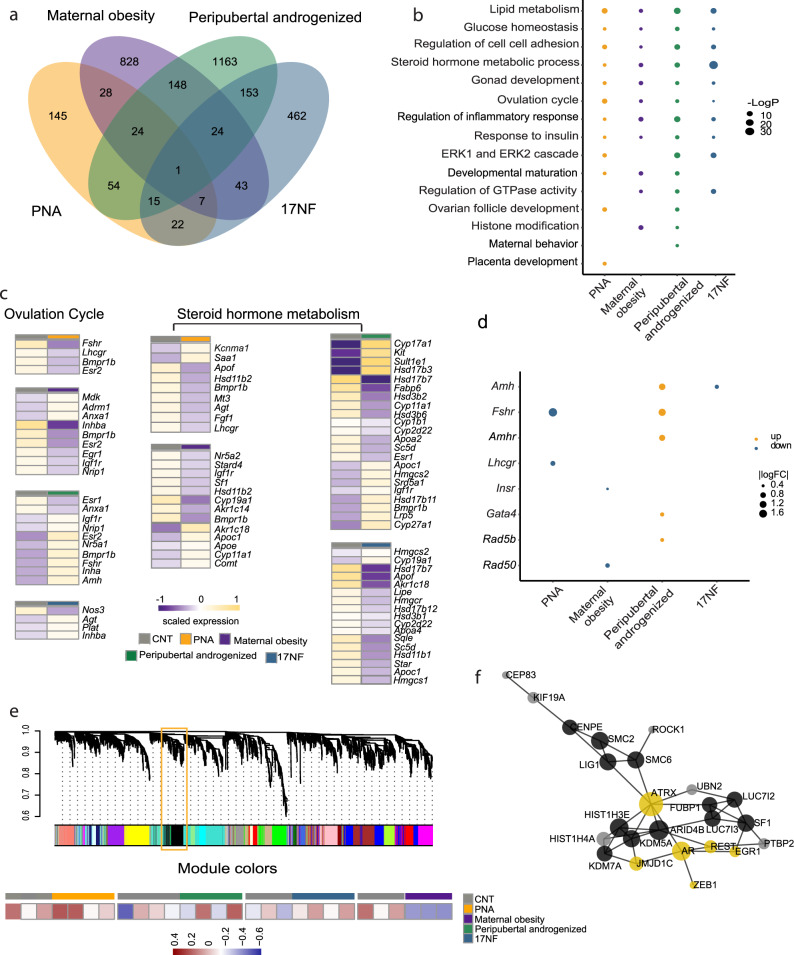
Common and distinct transcriptomic signature of the ovary. **a** Venn diagram of DEGs across all animal models (PNA model, *n* = 3 in control + vehicle, *n* = 4 in Prenatal DHT; peripubertal androgenized model, *n* = 4 in control + vehicle, *n* = 4 in Peripubertal DHT; 17NF, *n* = 3 in control, *n* = 4 in 17NF; maternal obesity, *n* = 3 in control, *n* = 3 in maternal obesity). **b** Comparison of enriched gene ontology term of DEGs across all animal models. **c** Heatmap of expression of DEGs enriched in Ovulation cycle and Steroid hormone metabolism in PNA, maternal obesity, Peripubertal androgenized, and 17NF respectively. **d** Overlapped DEG genes of ovary tissue with GWAS genes women with PCOS in PNA, maternal obesity, Peripubertal androgenized, and 17NF. **e** Module clustering tree diagram across animal models. Key module gene network involved in PCOS-like animal models. **f** Protein–Protein Interaction network of genes in black module (black module in **e**). PPI Data are retrieved from String database. *n* = number of animals.

One of the hallmarks of PCOS is chronic anovulation and the ovaries of women with PCOS contain more small antral follicles than normal ovaries. The irregular estrous cycle is one of the most prominent phenotypes in these PCOS-like mouse models. To further explore the underlying transcriptomic change that might be leading to the ovarian phenotypes, we first conducted DEGs analysis in the ovary among the PCOS-like mouse models and the maternal obesity model (Fig. 3a, and Table S3). The greatest number of DEGs is found in the peripubertal androgenized model coinciding with a strong reproductive phenotype induced by adult programming of androgen. We find 16 common DEGs among the three PCOS-like mouse models and 1 DEG (*Heph*) shared by all four models. To compare the common and unique pathways influenced by differential programming, we did a GO enrichment analysis of biological processes on DEGs (Table S4) in all four animal models. We identified pathways involved in metabolism, glucose homeostasis, steroid hormone metabolic process, response to insulin, and gonad development (Fig. 3b). The common pathway among the three PCOS-like mouse models is the ERK1 and ERK2 cascades. The ovarian follicle development pathway is shared between PNA and peripubertal androgenized models in agreement with the effects of DHT exposure. Notably, the unique pathway in PNA is placenta development, which reflects PNA modeling strategy, and maternal behavior in the peripubertal androgenized mouse model, which is in line with our previous observations that PNA and peripubertal androgen exposure induce anxiety-like behavior^29^.

To understand how the time point and dose of androgen and diet exposure affect the ovarian gene expression profile in these mouse models, we examined all DEGs that are involved in ovulation and identified several common and unique genes (Fig. 3c, Supplementary Data 2). *Bmpr1b* and *Fshr* are downregulated in PNA but upregulated in the peripubertal androgenized model indicating that the window of androgen exposure is critical and results in a different outcome in the ovulation cycle. The *Esr2* expression, on the other hand, is downregulated in both PNA and peripubertal androgenized mice. *Esr2*-mediated signaling is predominant in ovarian granulosa cells and plays an important role in follicle maturation and ovulation^30^. PNA and maternal obesity models, representing fetal programming, shared two downregulated genes *Bmpr1b* and *Esr2*, and *Inha* is upregulated in the peripubertal androgenized and in the 17NF models, respectively. Different ovarian steroid hormones are involved in regulating timely ovulation in female mammals. We found that the steroid hormone biosynthesis is also affected in the PNA, in the peripubertal androgenized, and in the diet-induced obesity models (Fig. 3c, Supplementary Data 2). In the PNA and maternal obesity models, respectively, *Hsd11b2* is commonly downregulated, and in the peripubertal androgenized and 17NF models are *Hsd17b7*, *Hsd3b1*, *Hsd3b2, Hsd3b6*, *Hsd17b7*, *Hsd3b1*, and *Hsd11b2* dysregulated.

To further explore the androgen exposure effects, we compared the DEGs with genes linked to PCOS SNPs identified in Genome-Wide Association Studies (GWAS) studies. We found that seven PCOS risk genes were dysregulated in our animal models (Fig. 3d). The reproductive risk genes (*Fshr* and *Lhcgr*) are downregulated in the PNA model, and the metabolic risk genes (*Insr* and *Rad50*) are downregulated in the maternal obesity model. While the peripubertal androgenized model has both metabolic and reproductive risk genes upregulated (*Amh*, *Amhr2*, *Fshr*, and *Rab5b*), and in the 17NF model *Amh* is downregulated (Fig. 3d). These findings support the relevance of the different PCOS-like mouse models reflecting common PCOS-related ovarian cellular processes.

To find common gene sets signature across PCOS-like mouse models, we conducted a weighted gene co-expression network analysis (WGCNA) to define the relationship between gene sets (modules) and phenotype features^31^. We found 16 significant gene modules in PNA, peripubertal androgenized, 17NF, and maternal obesity animal models. (Fig. 3e and Table S5) and module1 (MEblack) contained 215 genes that were expressed across the PNA, peripubertal androgenized, and 17NF models but not in the maternal obesity model. Gene network analyses of the 215 genes in the MEblack module revealed the androgen receptor and its interactome are preserved across the three PCOS-like mouse models, but not in the obesity model. The low-density lipoprotein receptor (LDLR) mRNA binding and splicing proteins encoded by *Sf1* and *Fubp1* are also preserved in this module (Fig. 3f, Supplementary Fig. 1a and Table S5).

### Ligand-receptor interaction between MII oocytes and ovary modulated by androgen exposure at different developmental windows

**Fig. 4:**
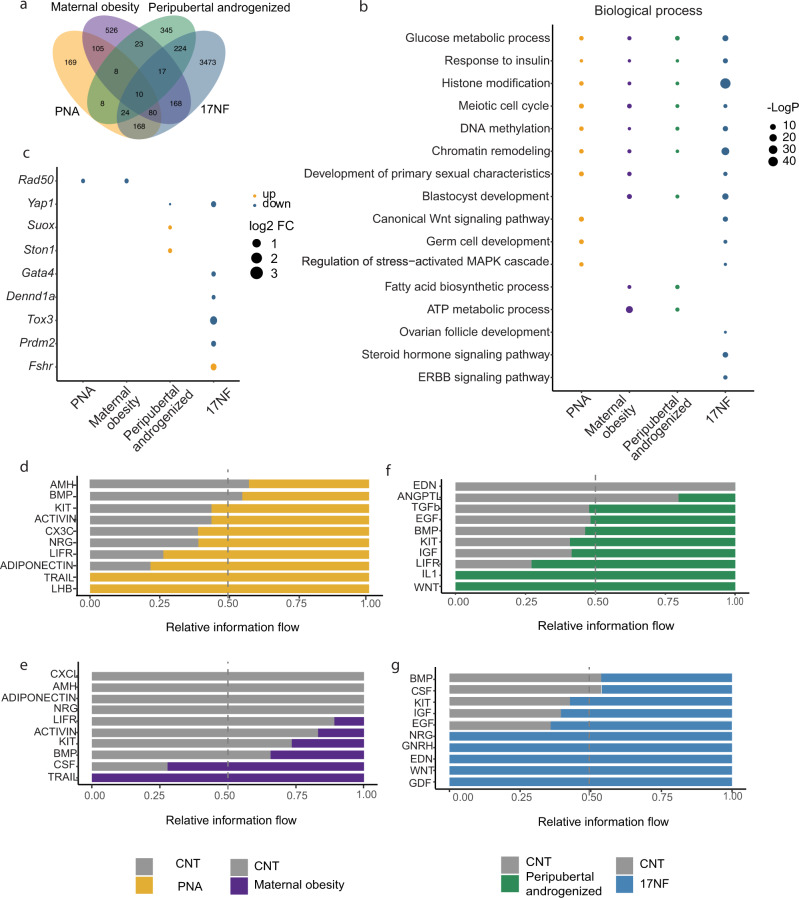
Common and distinct transcriptomic signature of MII oocytes. **a** Venn diagram of DEGs of MII oocytes across all animal models (PNA model, *n* = 8/3 animals in control + vehicle, *n* = 10/2 animals in Prenatal DHT; maternal obesity, *n* = 8/3 animals in control, *n* = 15/3 animals in maternal obesity; peripubertal androgenized model, *n* = 56/8 animals in control + vehicle, *n* = 67/8 animals in peripubertal DHT; 17NF, *n* = 16/3 animals in control, *n* = 16/3 animals in 17NF). *n* = number of MII oocytes. **b** Comparison of enriched gene ontology term of DEGs in MII oocytes across all animal models. **c** Overlap DEG genes of MII oocytes with GWAS genes women with PCOS in PNA, maternal obesity, Peripubertal androgenized, and 17NF. **d**–**g** Significant signaling pathways were ranked based on their differences in overall information flow within the inferred networks between control and treatment group in PNA, maternal obesity, Peripubertal androgenized, and 17NF, respectively.

To further understand how different windows of androgen and maternal obesity exposures affect the development and gene expression of MII oocytes, we carried out the single-cell transcriptomic analysis of MII oocytes from the four animal models. Unique and overlapped genes are presented in the Venn diagram (Fig. 4a and Table S6). Interestingly, most DEGs were detected in the MII oocytes of the 17NF model suggesting that genetic modification of theca cells greatly affects the oocytes. In addition, we found 34 common DEGs among the three PCOS-like models and 10 DEGs (*Obox5, Tcl1b1, Mki67, Rapgef5, Mphosph6, Afap1l2, Gmnn, Mfap1b, Gadd45gip1, Ints9*) are shared by all four models. To define the functional role of these DEGs, GO annotation revealed common biological processes involved in all models such as glucose metabolic process, response to insulin, meiotic cell cycle, etc. One pathway i.e., development of primary sexual characteristics was shared by the three PCOS-like mouse models. And 17NF models contained several unique pathways such as ovarian follicle formation, steroid hormones, and ERBB signaling pathways, suggesting strong effects of local increase in androgen due to genetic modification of theca cells (Fig. 4b and Table S7). Several DEGs in MII oocytes in each model were also linked to PCOS susceptible gene loci identified from GWAS (Fig. 4c). We found that 17NF showed the most common DEGS with PCOS GWAS genes compared to other models. As there is plentiful evidence to show that androgens stimulate the growth of both preantral and antral follicles and impair the follicle maturation^32^, and as MII oocytes and surrounding follicular niche cells communicate bi-directionally in the ovary, we further investigate ligand-receptor interactions between MII oocytes and ovaries by using a ligand-receptor database (CellChat DB)^33^.

By ligand-receptor analysis, we identified several differentials signaling pathways in each animal model. The members of BMP signaling have been reported strongly related to follicular development and involved in PCOS pathology^34^. In the PNA, maternal obesity, and the 17NF models, we found that BMP signaling is reduced compared to control (Fig. 4d–g): the ligand Bmp4 and its receptors [Bmpr1a + Bmpr1b + Bmpr2] in PNA; Bmp7 [Bmpr1a + Bmpr1b + Bmpr2] in maternal obesity; Bmp5 + Bmp15 [Bmpr1b + Bmpr1a] in 17NF model, respectively. Interestingly, AMH signaling is reduced in PNA and maternal obesity models (Fig. 4d, e). This is likely due to androgen-induced reduction of oocyte-specific BMP which normally stimulates AMH levels. We found several cell proliferation signaling pathways such as EGF signaling: Tgfa [Egfr+Erbb4], IGF signaling: Igf1 [Igfr+Itga6] to be increased in the peripubertal in line with previous studies^35^. KIT signaling involved in the regulation of follicle growth and oocytes development is increased in PNA, peripubertal androgenized model and in 17NF models, respectively. Notably, we found activation of inflammatory pathway including TRAIL: Tnfs10 [Tnfrs10b]; CSF: Il34 [Csfr and Csf1-Csfr1]; LIFR: Ctf [Lifr] in all models. In the PNA model, we found increased enrichment of ligand-receptor pairs that are potentially driven by in utero androgen exposure. Specifically, activin: Inhba [Acvr1b + Acvr2a], adiponectin: Adipoq [Adipor2 and Adipor1], tumor necrosis factor-related apoptosis-inducing ligand (TRAIL) Tnfs10 [Tnfrs10b] and luteinizing hormone subunit beta (LHB) Lhb [Lhcgr] (Fig. 4d). In the peripubertal androgenized model, ligand-receptor pairs in CCL with Ccl25 [Ackr4], IL1 with Il1a [Il1r2] and Il1b [I1r2] and WNT (Wnt1 [Fzd1 to Fzd10 + Lrp5]) pathways were enriched compared to control (Fig. 4f). Notably, several more signaling pathways for ligand-receptor pairs were enriched in the 17NF model such as NRG [Erbb4-Nrg2), with GnRH [Gnrh1, Gnrhr] and GDF Gdf9 [bmpr2] uniquely found in 17NF model compared to control (Fig. 4g). Collectively, these results suggest that signaling pairs involved in immunity, development, and fertility are differently affected in each model.

### Distinct metabolic pathways in MII oocytes affected by androgen exposure at different developmental windows

**Fig. 5:**
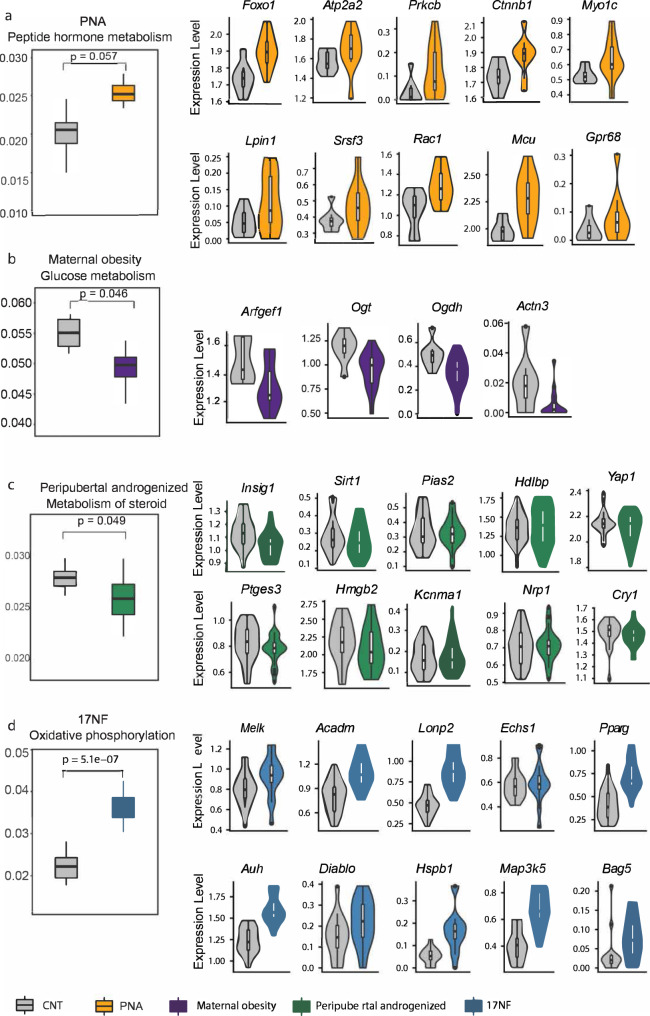
Metabolic pathway analysis of MII oocytes. **a**–**d** Boxplot showing the key metabolic pathways in PNA (*n* = 8/3 animals in control + vehicle, *n* = 10/2 animals in prenatal DHT), Maternal obesity (*n* = 8/3 animals in control, *n* = 15/3 animals in maternal obesity), Peripubertal androgenized (*n* = 56/8 animals in control + vehicle, *n* = 67/8 animals in Peripubertal DHT) and 17NF model (*n* = 16/3 animals in control, *n* = 16/3 animals in 17NF), respectively. Violin plot with an overlay of boxplots showing the DEGs expression in each metabolic pathway. For the boxplot within each violin plot, middle lines indicate median values, boxes range from the 25th to 75th percentiles. *n* number of MII oocytes.

Emerging evidence indicates that metabolism is a major determinant of oocyte quality^36^. Besides the ligand-receptor signaling effects, to investigate if any metabolic changes affect oocytes quality, we quantified the metabolism activity of MII oocytes in the PCOS-like mouse models and in the maternal obesity model. Peptide hormone metabolism is affected in the PNA model (Fig. 5a, Supplementary Data 3). Several DEGs involved in this pathway such as *Foxo1, Atp2a2, Prkcb, Ctnnb1, Lpin1, Srsf3, Rac1* might be crucial to drive androgen effects. *Foxo1* belongs to the forkhead transcription factor family known to have cellular functions including cell growth and differentiation. The expression of *Foxo1* is highly increased in cumulus cells of women with PCOS. *Foxo1* is a key downstream molecule of IGF1 signaling, regulating the circulatory metabolism and hormone levels in hypothalamus-pituitary axis and adipose tissue. Glucose is essential to generate ATP for energy in the metabolite cumulus-oocyte complex. Oocyte itself is poor to metabolize glucose, instead, the oocyte is reliant on cumulus cells to take up glucose on its behalf. In the maternal obesity model, the glucose metabolism is significantly decreased, indicating delayed oocytes maturation in offspring of obese mothers (Fig. 5b, Supplementary Data 3). The DEGs involved in this pathway are *Arfgef1, Ogt, Ogdh,* and *Actn3*. In the peripubertal androgenized model, metabolism is significantly reduced and the expression of DEGs involved in steroid pathway like *Yap1*, linked to PCOS susceptible gene loci identified from GWAS; *Sirt1* in regulation of systemic energy and steroid hormone homeostasis are also decreased (Fig. 5c, Supplementary Data 3). We also identified that the unique oxidative phosphorylation metabolism pathway is significantly upregulated in 17NF mice model (Fig. 5d, Supplementary Data 3).

### Adipose tissue largely affected independent of exposure window to androgen

**Fig. 6:**
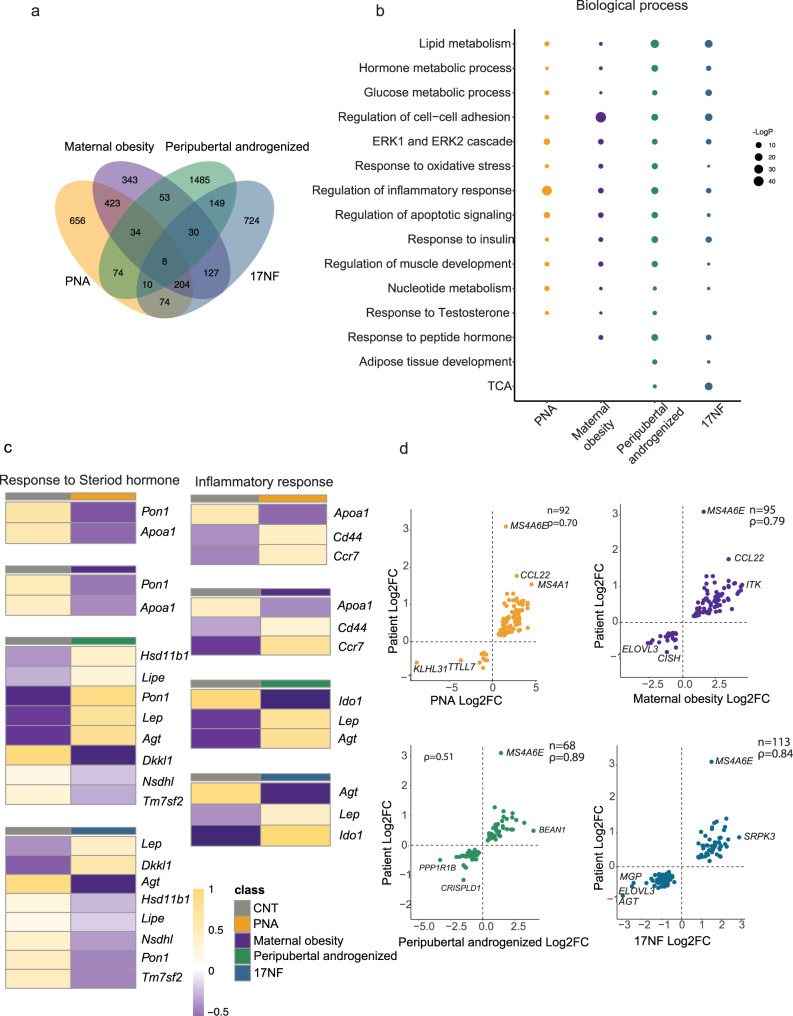
Common and distinct transcriptomic signature of adipose tissue. **a** Venn diagram of DEGs across all animal models (PNA model, *n* = 3 in control + vehicle, *n* = 4 in Prenatal DHT; maternal obesity, *n* = 3 in control, *n* = 3 in maternal obesity; Peripubertal androgenized model, *n* = 4 in control + vehicle, *n* = 4 in Peripubertal DHT; 17NF, *n* = 3 in control, *n* = 3 in 17NF). **b** Comparison of enriched gene ontology term of DEGs in adipose tissue across all animal models. **c** Heatmap of expression of DEGs enriched in Response to steroid hormone, inflammatory response in PNA, maternal obesity, Peripubertal androgenized, and 17NF, respectively. **d** Scatter plot comparing alteration of gene expression between disease and controls in mouse models and patients^41^. Yellow dots indicate the genes whose expression alterations in mouse models are in line with patients. Blue dots indicate the genes whose expression alterations in mouse models are opposite to patients. The spearman correlation coefficient in corresponding group is also shown. n = number of animals.

Obesity is a common feature in women with PCOS and adipose tissue dysfunction is likely involved in the development of the syndrome and associated metabolic disturbances. All models used have increased fat mass and enlarged adipocytes (Fig. 1a) indicating aberrant adipose tissue function and metabolic dysfunctions. Transcriptomic analysis of subcutaneous adipose tissue showed common and unique DEGs (Fig. 6a and Table S8). The largest number of DEGs (1910) was found in the peripubertal androgenized model suggesting potent adult programming of DHT in adipose tissue (Fig. 6a). Total, 18 common DEGs were detected in the three PCOS-like mouse models, whereof 8 were shared by all models. Subsequent functional analysis of DEGs in each model shows shared biological processes; lipid, glucose, and nucleotide metabolism, regulation of inflammatory response, and response to testosterone, except in 17NF model (Fig. 6b). Notably, adipose tissue development was enriched in peripubertal androgenized and 17NF models as adult programming models. We then selected DEGs in each model involved in the same pathways and show that in response to a steroid hormone, *Apoa1* and *Pon1* were both downregulated in PNA and maternal obesity models indicating a fetal programming effect, whereas *Hsd11b*, *Lep*, *Lipe*, and *Sqle* were affected in the peripubertal androgenized and 17NF models (Fig. 6c, Supplementary Data 4). Notably, *Pon1* was influenced by both differential androgen exposures and by maternal obesity in adipose tissue. However, *Pon1* expression is upregulated in PNA and peripubertal androgenized models whereas it was downregulated in 17NF and maternal obesity models. *Pon1* gene encodes the calcium-dependent antioxidant enzyme paraoxonase1. It has been shown that there is an inverse correlation between *Pon1* and hyperandrogenism due to PCOS^37^. As obesity is linked to chronic inflammation, we also analyzed the DEGs in the regulation of inflammatory response. *Ccr7*, a chemokine receptor expressed in various immune cells and is linked to obesity as its knockout in mice results in protection from diet-induced obesity^38^. Upregulation of *Ccr7* in adipose tissue indicates inflammation due to increased adiposity (Fig. 6c, Table S9 and Supplementary Data 4). This notion is further supported by higher expression pattern of *Cd44* in the PNA and maternal obesity models. *Cd44* likely plays a regulatory role in obesity-linked metabolic syndrome^39^. In peripubertal androgenized and 17NF models, the *Ido1*, an anti-inflammatory gene, was downregulated, and upregulated, respectively (Fig. 6d and Table S9). In contrast to fetal programming (PNA and maternal obesity), peripubertal androgen exposure and 17NF showed distinct impaired glucose homeostasis (Fig. 1a). Our transcriptomic profile of adipose tissue showed that upregulated *Mup1* expression is common to PNA, maternal obesity, and peripubertal androgenization (Supplementary Fig. 2). The functional enrichment analyses in these models showed that *Mup1* is involved in glucose homeostasis an indicator of insulin sensitivity^40^. In the 17NF model, functional enrichment of DEGs in the glucose homeostasis displayed upregulation of *Insr*, *Irs1*, *Ppara, Acacb, Dgal2, and Rorc* (Supplementary Fig. 2). Taken together, independent of exposure window to androgen or maternal obesity, our findings reveal that adipose tissue is largely affected likely contributing to aberrant adipose tissue function and glucose intolerance often observed in these models (Fig. 6c). Next, we performed correlation analyses between fold changes of DEGs in each mouse model with published fold changes of DEGs in adipose tissue of women with PCOS^41^ and found strong correlation between each model and women with PCOS (Fig. 6d). Interestingly, *Ms4a6e* is common to all PCOS-like mouse models also with a high fold-change in expression in adipose tissue of women with PCOS (Fig. 6d and Table S10), both fetal programming models, PNA and the maternal obesity, showed a strong correlation with *CCL22* expression in women with PCOS (Fig. 6d). This finding suggests that gene expression of subcutaneous adipose tissue in all mouse models recapitulates gene expression of women with PCOS.

### Comparison of transcriptomic profiles across key tissues and MII oocytes in different mouse models

**Fig. 7:**
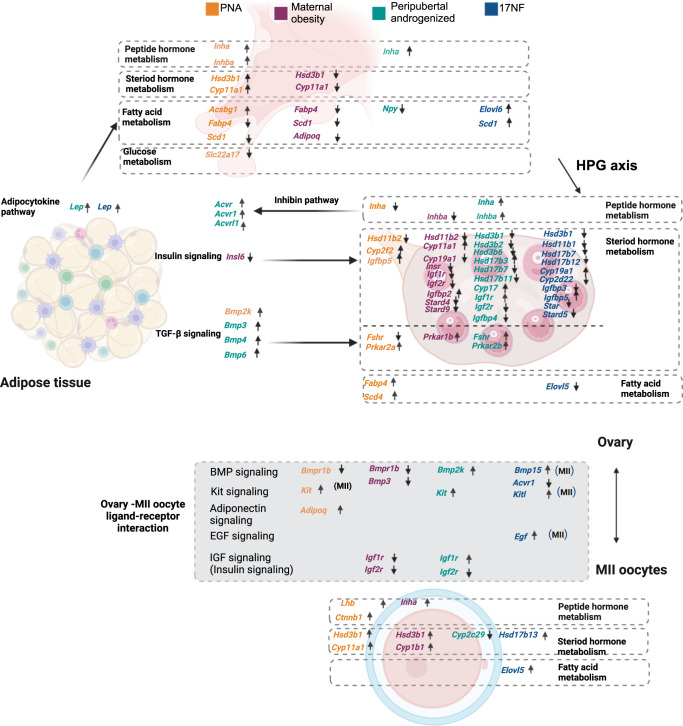
Transcriptomic interaction among hypothalamus, ovary, adipose tissue, and MII oocytes in PCOS-like models. **a** Transcriptomic crosstalk and unique gene signatures among hypothalamus, ovary, adipose tissue, and MII oocytes in PNA, maternal obesity, Peripubertal androgenized, and 17NF, respectively.

Next, we explored the DEGs-affected metabolic pathways including peptide hormone metabolism, steroid hormone metabolism fatty acid metabolism among target tissues in each animal model, and found common and unique DEGs and possible signaling pathways across all tissue in PCOS-like mouse models and maternal obesity model. (Fig. 7, Supplementary Fig. 3, Tables S11–13). By ligand-receptor analyses, we identified possible signaling pathways such as BMP signaling pathway, Kit signaling pathway, adiponectin unique affected by PNA model, EGF and IGF (insulin signaling pathway) pathways affecting MII oocytes quality.

The ovary and adipose tissues shared many common DEGs among the PCOS-like mouse models indicating a transcriptional network modulated by hyperandrogenism in the peripheral tissues, with most DEGs in the peripubertal androgenized model followed by the 17NF and the PNA mouse models (Table S11). Interestingly, as a fetal programming effect, first-generation female offspring in the maternal obesity model had more common dysregulated genes in the ovary and adipose tissue compared to the offspring in the PNA model. Several genes in the PNA, peripubertal androgenized, and 17NF models established a regulatory network in the hypothalamus-ovary-adipose axis with either exact match or family member match gene expression. For example, in the PNA model, *Atp1b1* and *Atp2a3* are downregulated in the hypothalamus and adipose tissue, respectively, showing family members in the transcriptomic network. To extend exploration, we consider a steroid hormone metabolism-regulating panel of genes. The *Hsd3b1* expression is affected by androgen and maternal obesity in the fetal programming milieu. In the PNA model, *Hsd3b1* is upregulated in the hypothalamus and MII oocytes, whereas in maternal obesity *Hsd3b1* is downregulated in the hypothalamus and upregulated MII oocytes (Table S12). On the other hand, adult programming displayed a consistent pattern of gene expression as shown in the peripubertal androgenized and 17NF models. Besides common gene signatures in different tissues and PCOS-like mouse models, we demonstrated a unique gene influenced by differential programming resulting from hyperandrogenism and exposure to maternal obesity with downregulated expression of *Cfd*, which is not affected in peripubertal androgenized and 17NF models. Additionally, in the PNA model, *Fabp4* is downregulated in the hypothalamus and upregulated in the ovary, and *Fabp3* (family member match of *Fabp4*) is upregulated in adipose tissue, indicating an influence of PNA on lipid metabolism in different tissues. Peripubertal androgenized and 17NF models also showed a unique differential gene expression pattern of *Car3*, *Cyp27a1*, *Car5b*, and *Cyp2d22* in the ovary and adipose tissue (Fig. 7, Table S13).

## Discussion

To model PCOS pathogenesis such as androgen excess, several mouse models have been developed to study pathophysiology. However, comprehensive transcriptomic profiling at the molecular level across key reproductive and metabolic tissues among different mouse models is still lacking. As each model could only mimic certain pathophysiological features, it is important to understand how the same target tissue is differentially affected among different models. Therefore, we performed extensive transcriptomic profiling to define the molecular effects of androgen in different PCOS-like mouse models in comparison to maternal obesity.

Like other complex diseases such as type 2 diabetes^42,43^ PCOS is a highly heritable disorder. However, only a small proportion of the heritability can be accounted for by the ~20 susceptibility loci identified by GWAS^15^. Nonetheless, reproductive and metabolic phenotypes are associated with specific PCOS susceptibility loci, supporting the role of genetic factors in pathogenesis^15^. Indeed, we recently showed that ~70% of daughters of women with PCOS receive the diagnosis of PCOS around their twenties^13^. Moreover, we also showed that transmission of PCOS across generations in mice occurs as a result of maternal androgen exposure implying that the maternal-fetal environment may account for the mother-to-daughter inheritance of the syndrome in a non-genetic manner^13^. Clinical evidence of a hyperandrogenic fetal environment is that PCOS women’s daughters display a longer anogenital distance^44^ and higher levels of facial sebum production^45^ at birth, markers of in utero hyperandrogenism. Amniotic fluid from daughters of women with PCOS showed significantly elevated testosterone compared with control women during mid-gestation^46^, which represents a critical window for the development of the hypothalamus. Exposure to elevated level of androgen might result in fetal programming of the germ cells, hypothalamus, and other targeted tissues.

The adipocyte-fatty-acid-binding protein (FABP4) is an adipokine involved in the regulation of whole-body insulin sensitivity, as well as lipid and glucose metabolism, and has been implicated in the development of PCOS through regulation of transcription and/or protein alterations^47^. These previous reports support our findings that the *Fabp4* gene expression is dysregualted in the hypothalamus only in our fetal programming mouse models (PNA and diet-induced maternal obesity), but not in the adult programming models (peripubertal androgenization and 17NF). In the peripubertal androgenization mice, but not in the fetal programming models, the expression of *Gal* is downregulated in the hypothalamus, which codes neuropeptide galanin. Serum level of Galanin has been implicated as a risk factor in metabolic and cardiovascular diseases in women with PCOS^48^. The expression of *Acot7, Elovl6, Fasn, Hmgcs1*, and *Scd1* genes involved in triglycerides and cholesterol metabolism display differential expression patterns and are upregulated in the PNA and 17NF models and downregulated in maternal obesity mouse model in line with previous observations^47^.

Interestingly, the strongest GO enrichment pathways in the hypothalamus were found in the PNA and maternal obesity models followed by peripubertal androgenized with the least enrichment in 17NF mouse models indicating that the fetal programming exerts a stronger effect on the hypothalamus compared to adult programming. That adult programming has less pronounced effect on hypothalamic gene expression is supported by the recent finding that peripubertal androgen exposure does not impact luteinizing hormone pulse frequency^49^ as it does in the PNA model^22,23^. Thus, the discrepancies in the hypothalamic transcriptomic and functional enrichment profiling among the mouse models suggest there are critical windows for exposure of androgens and obesity that affect the development of hypothalamus. The hypothalamic neurogenesis occurs between E10.5 and E16.5 in mice followed by gliogenesis and terminal differentiation which overlaps with the sexual maturation of the animal^50^. The different time points of androgens exposure affect distinct stages of hypothalamus development. Hypothalamus also regulates the reproductive axis and both PNA and peripubertal androgenized mouse models showed GO annotation linked to gonad development.

To extend our findings in the hypothalamus to the reproductive axis in these animal models, we performed transcriptomic analyses also of the ovaries and MII oocytes from these models. The ovary is a heterogeneous organ and comprises different cell types. For functional reproductive life, both autocrine and paracrine communications among these cells play an important role in follicle growth and oocyte maturation. To understand the temporal influence of androgen in differential programming, we analyzed ligand-receptor interaction between the ovary and MII oocytes in these PCOS-like animal models. Our study showed common and unique ligand-receptor interaction in ovary-MII oocytes in fetal (PNA and maternal obesity) and adult programming PCOS-like mouse models. The 17NF model, a transgenic model with selective overexpression of nerve growth factor in the ovarian theca-interstitial cells, directly influence follicular development displays unique differential ligand-receptor pairs such as GDF (Gdf9-bmpr2). *Gdf9* is an oocyte-specific gene, playing an important role in oogenesis^51^. Moreover, we identified the differential signaling pathways such as BMP signaling affecting follicle development; EGF and IGF signaling affects cell proliferation; KIT signaling that is import for oocyte maturation in differential programming PCOS-like mouse models; adiponectin signaling as a positive regulator of metabolic function^52^. Our ligand-receptor interaction analysis provides a resource to study the signaling changes in the ovary. Notably, we identified the biological processes that are involved in metabolic pathways in MII oocytes in all PCOS-like mouse models and in the maternal obesity mouse model.

It has been reported that hypothalamic *Gmp6a*, *Rgs2, and Txnip* are likely to be involved in an estrous cycle in female mice^53^. Moreover, *Rgs2* is regulated by GnRH^54^, which implies the hypothalamus-ovary axis in the regulation of estrous cyclicity. In support of these previous findings, we found that *Gpm6a* expression is downregulated in the PNA model, and *Txnip* and *Rgs2* are upregulated in the hypothalamus with the latter also detected by WGCNA analysis in the ovary supporting the strong link between hypothalamus-ovary axis in the regulation of estrous cyclicity. Peripubertal androgenization also affects the estrous cycle gene expression with downregulation of *Gal* in the hypothalamus^53^. These findings support that *Gmp6a*, *Rgs2, Txnip*, and *Gal* dysregulation are linked to irregular estrous cycle in the PNA and peripubertal androgenized mouse models. Moreover, functional analysis showed similar activated pathways among the four models. Our in-depth assessments of DEGs related to estrus cyclicity revealed that fetal programming, as in PNA and diet-induced obesity, showed a distinct and common expression pattern compared to those influenced by adult programming. Additionally, activated ovarian pathways linked to the placenta development (*E2f8, Fgfr2, Cited2, Cdh1, Krt19, Socs3*) in PNA and maternal behavior (*Oxtr, Kalrn, Crebrf*, and *Pten*) in peripubertal androgenized mice are unique considering the temporal difference in androgen exposure.

The function of subcutaneous fat is coordinated by neuroendocrine and hormonal cues from outside of the fat depot^55^. Moreover, hyperandrogenism affects adiposity and adipogenesis. Subcutaneous adipose tissue transcriptomic profiling displayed dysregulated *Pon1* expression and is affected by differential exposure to hyperandrogenism as well as exposure to maternal obesity. It has been shown that dyslipidemia in women with PCOS is linked to PON1, an oxidative enzyme associated with apoA1 on HDL particles^56^. In line with this observation, all PCOS-like mouse models and the maternal obesity model shared GO enrichment related to response to oxidative stress: in PNA and maternal obesity and peripubertal androgenized and 17NF. To link our preclinical findings in adipose tissue to humans, we overlapped adipose tissue DEGs in the different mice models and with adipose tissue DEGs of women with PCOS^41^. The result showed that altered *MS4A6E* gene expression is shared by all PCOS-like models, a gene that has been shown to be involved in neurodegenerative disorders^57^. Moreover, we found that adult androgen programming has the highest number of common DEGs between the ovary and adipose tissue with the lowest number of common DEGs in maternal obesity followed by PNA mouse models. Common gene regulation between the hypothalamus, ovary, and adipose tissue was found in PNA (*Ccl* members), peripubertal androgenized (*Wfdc* members), and 17NF (*Elovl* members).

How differential gene expression in target tissues may contribute to phenotypic changes in PCOS-like and maternal obesity mouse models is unclear. Therefore, we here characterized gene expression at the bulk level in our unique collection of tissue and MII oocytes from three well-phenotyped PCOS-like mice models and one diet-induced maternal obesity model. Although our study contained a small size for tissue samples and applied *p* value rather than *q* value to identify DEGs, it provides a comprehensive transcriptome resource of key target tissues in three mouse models of PCOS and one of maternal obesity for the research community serving as a reference in the model selection. Moreover, it increases our understanding of molecular features underlying phenotypic alterations in each model allowing comparison with human transcriptomic profiling data for future mechanistic research on PCOS pathogenesis. In addition, several common functional pathways are identified among all mouse models despite different gene targets. The peripheral tissues: ovary and adipose tissue are more affected than the hypothalamus in the peripubertal androgenized, the 17NF, and in the maternal obesity mouse models. Not surprisingly, the fetal programming exerts a strong effect on hypothalamic gene expression as compared to the peripubertal and adult programming, and the transgenic 17NF mouse model exerted the strongest effect on ovarian gene expression profile. The adipose tissue of the peripubertal androgenized model was the most affected which is in line with the phenotype of this model with increased fat mass and altered glucose metabolism. Importantly, the increased/decreased *MS4A6E* expression in subcutaneous adipose tissue of women with PCOS and all mouse model, highlights a conservative disease mechanism.

## Methods

### Ethical approvals

All animal experiments were approved by the Stockholm Ethical Committee for Animal Research (10798-2017 and 17538-2020) in accordance with the legal requirements of the European Community (SJVFS 2017:40) and the directive 2010/63/EU of the European Parliament on the protection of animals used for scientific purposes. Animal care and procedures were performed in accordance with guidelines specified by European Council Directive and controlled by Comparative Medicine Biomedicum, Karolinska Institutet, Stockholm, Sweden.

### Experimental animals

All mice were maintained under a 12-h light/dark cycle and in a temperature-controlled room with ad libitum access to water and a diet. Prior to starting the experiments, the number of animals required for the experiments was estimated from our previous work based on the same model where the success of the breeding was ~60% of the F0 dams^13,21,25,58^.

### Prenatal androgen-exposed model

To generate prenatal androgen-exposed offspring^13^, 3-week-old female C57Bl/6 J mice (Janvier Labs, Le Genest-Saint-Isle, France) were fed on control diet (Research Diets, D12328) comprising 11% fat, 73% carbohydrates [0% sucrose], and 16% proteins. These mice were randomly divided into the control and PNA groups after mating with male mice fed on chow diet and were subcutaneously injected from E16.5 to E18.5 with 50 µl of a solution containing (**1**) a mixture of 5 µl benzyl benzoate (B6630; Sigma-Aldrich) and 45 µl sesame oil (S3547; Sigma-Aldrich, St. Louis, Missouri, USA) i.e. vehicle (control), or (**2**) 250 µg dihydrotestosterone (5α androstan-17β-ol-3-one, A8380; Sigma-Aldrich, St. Louis, Missouri, USA) dissolved in a mixture of 5 µl benzyl benzoate and 45 µl sesame oil. First-generation female offspring were subjected to phenotypic testing prior finalization which has been described in detail elsewhere^13^.

### Peripubertal DHT-exposed model

To generate hyperandrogenemic females, 4-week-old adult female mice C57Bl/6 J mice (Janvier Labs, Le Genest-Saint-Isle, France) were implanted subcutaneously with a 10-mm length (pellet) of DHT or, as control, a no-DHT pellet^59^. This 10-mm pellet contained 5.24 mg DHT. Before implantation, the pellets were equilibrated in saline for 24 hours at 37 °C^59^. DHT pellets were prepared as described^60^.

### 17NF mouse model

The breeding, genetic background, and generation of the transgenic 17NF mice (MGI Cat# 5662267, RRID: MGI:5662267) has previously been described in detail^18,19^. These mice overexpressed NGF driven by the 17alpha-hydroxylase gene promoter in theca cells of the ovary. The transgene expression in each batch of homozygous 17NF mice was confirmed by genotyping.

### Diet-induced maternal obesity model

To generate the diet-induced maternal obesity model, 4-week-old female C57Bl/6 J mice (Janvier Labs, Le Genest-Saint-Isle, France) were fed an HFHS diet (Research Diets, D12331) comprising 58% fat, 26% carbohydrates [17% sucrose], and 16% proteins or a control diet (Research Diets, D12328) for 6 weeks prior mating^13^. Eight- to 12-week-old male mice fed on chow diet were used for mating and fed an in-house chow diet (*R34*, Lantmännen, Kimstad, Sweden). First-generation female offspring were subjected to phenotypic testing which has been described in detail elsewhere^13^.

### MII oocytes and tissues collection

To collect MII oocytes from PNA, peripubertal DHT, 17NF, and maternal obesity mouse models, 20-week-old female were superovulated by injecting 5 IU of pregnant mare’s serum gonadotropin (PMSG) (Folligon, MSD Animal Health Care, Stockholm, Sweden) followed by 5 IU of human chorionic gonadotropin (hCG) (Pregnyl 5000IE, Merck Sharp & Dohme AB, Stockholm, Sweden) 48 h after PMSG priming. Cumulus-oocyte complexes were isolated at 16 h post-hCG injection from oviduct ampulla. Denuded single-MII oocytes were then obtained by removing the cumulus mass in M2 medium (M7167; Merck KGaA, Darmstadt, Germany) containing 0.3 mg/ml hyaluronidase (H3884; Merck KGaA, Darmstadt, Germany) at room temperature.

At finalization, mice were fasted for 2 hours before blood, oocyte, and tissue collection. Briefly, the subcutaneous adipose tissue, ovaries, and hypothalamus were quickly dissected on ice, snap-frozen in liquid nitrogen, and stored at −80°C.

### RNA isolation and bulk RNA-sequencing library preparation

Mouse subcutaneous adipose tissue, hypothalamus, and ovary were homogenized in 1 ml TRI reagent (T9424, Sigma-Aldrich). Total RNA was extracted as per the manufacturer’s instructions and quality was confirmed by Agilent 2100 bioanalyzer with RIN 6–7. Then 1 ng of total RNA was applied to bulk RNA-sequencing library. Sequencing libraries were generated according to Smart-seq3 protocol^61^. Briefly, polyA(+) RNA was reverse transcribed by Maxima H-minus reverse transcriptase (Thermo Fisher). The second-strand synthesis was conducted by a template-switching reaction and 12 cycle of PCR was performed for cDNA amplification by KAPA HIFI HotStart polymerase (Roche). Then cDNA was purified by 22% PEG (Sigma-Aldrich) beads. Aglient 2100 BioAnalyzer (Agilent Technologies) was performed to check the quality and quantity of cDNA libraries. Sequencing libraries were generated by tagmenting 200 pg cDNA using Nextera XT Tn5 transposase (Illumina) and amplified for 10 cycles.

### MII oocytes from PNA, maternal obesity, and 17NF models sequencing library preparation

Single- MII oocyte was prepared by Smart-seq2 protocol. Following cell lysis, polyA(+) RNA was captured by SuperScript II reverse transcriptase (Thermo Fisher), Template swishing reaction was utilizing for second-strand synthesis. cDNA amplification was prepared by 14 cycles of PCR reaction using KAPA HIFI HotStart ReadyMix (KAPA Biosystems) and the libraries were purified by magnetic beads. Aglient 2100 BioAnalyzer (Agilent Technologies) were applied for checking the cDNA quality. Sequencing libraries were generated by tagmentation 1 ng cDNA by Tn5 transposase and amplified for eight cycles.

### MII oocytes from Peripubertal model sequencing library preparation

Single-MII oocytes were prepared by Smart-seq3 protocol. Following cell lysis, polyA(+) RNA was reverse transcribed by Maxima H-minus reverse transcriptase (Thermo Fisher) as mentioned below. cDNA amplification was performed by PCR (14 cycles) followed by beads purification. Sequencing libraries were generated by Nextera XT Tn5 transposase (Illumina) and amplified for 10 cycles.

### RNA-seq data processing

Raw reads generated by Smart-seq3 protocol were mapped by zUMIs pipeline^62^. Raw reads generated by Smart-seq2 protocol were mapped to mouse reference genome (GRCm38/mm10) using STAR default arguments.

### DEG analysis and gene ontology analysis

DEGs were calculated using DESeq2 method (R package “DEGseq2 of version 1.34.0). DEG genes were defined by apeglm-shrunk Wald test *p* < 0.05 with log2 fold change >0.5 or log2 fold change <−0.5. Gene Ontology analysis of DEGs was performed by ‘Clusterprofiler’ R package and the biological terms were defined with FDR *q* < 0.05. Specifically, over representation analysis (ORA) is used in this package to determine whether the biological terms are enriched. The *p* values are calculated by hypergeometric distribution as the probability of these observed number of co-occurrence from random sampling for all the genes that have annotation. The *p* values are adjusted for multiple comparison confining *q* value <0.05 that inspect the proportion of false positive when the *p* < 0.05.

### WGCNA analyses

Normalized data were performed by ‘WGCNA’ R package. The power parameter with soft threshold of nine was selected by ‘pickSoftThreshold’ function. The Pearson correlation was used in the analyses and the correlation between module eigengenes, and different treatment of mice model were calculated to identify modules of interest that were significantly associated with the treatment of mice model.

### Cell-cell communication analyses

To investigate cell-cell communication between MII oocyte and ovary cells, ‘CellChat’ R package was performed to analysis the ligand and receptor between oocyte and ovary. Ligand-receptor pairs are defined based on ‘CellChatDB’ database. Based on biological function, all the interactions are grouped into 229 signaling pathway families. The differentially expressed signaling genes were identified by Wilcoxon rank sum test with significance level of 0.05.

### Metabolic pathway analysis of MII oocyte

MII oocytes metabolic pathway quantification was conducted by ‘scMetabolism’ R package. The function AUCell was used to quantify the metabolic activity after implement with Seurat pipeline. The genes for pathway analyses can be found online (https://github.com/wu-yc/scMetabolism).

### Statistics and reproducibility

Statistical analysis and data visualization in the present study was performed by using the R software (version 4.1.1, R Foundation for Statistical Computing, Vienna, Austria; http://www.r-project.org). Unless specifically stated, *p* or FDR values <0.05 were considered statistically significant.

### Reporting summary

Further information on research design is available in the Nature Research Reporting Summary linked to this article.

## Supplementary information

Supplementary Information

Description of additional supplementary files

Supplementary Data

Reporting summary

## Data Availability

Raw data on PNA, peripubertal androgenized, maternal obesity, and 17NF females are available. All raw and analyzed single-cell RNA-sequencing data of mouse MII oocytes from PNA, peripubertal androgenized, maternal obesity, and 17NF females are available at SRA database with accession number PRJNA856805.

## Code availability

Codes that support corresponding figures are available at GitHub (https://github.com/lunapei520/Transcriptomic-survey-of-key-reproductive-and-metabolic-tissues-in-mouse-models-of-polycystic-ovary-.git).
