# Supplementary material for: Transcriptomic survey of key reproductive and metabolic tissues in mouse models of polycystic ovary syndrome
Source: Commun Biol. 2023 Jan 18;6:69. doi: 10.1038/s42003-022-04362-0 (PMC9849269; doi:10.1038/s42003-022-04362-0)
Supplement: Supplementary file 1 — Supplementary Information [file 42003_2022_4362_MOESM1_ESM.pdf]

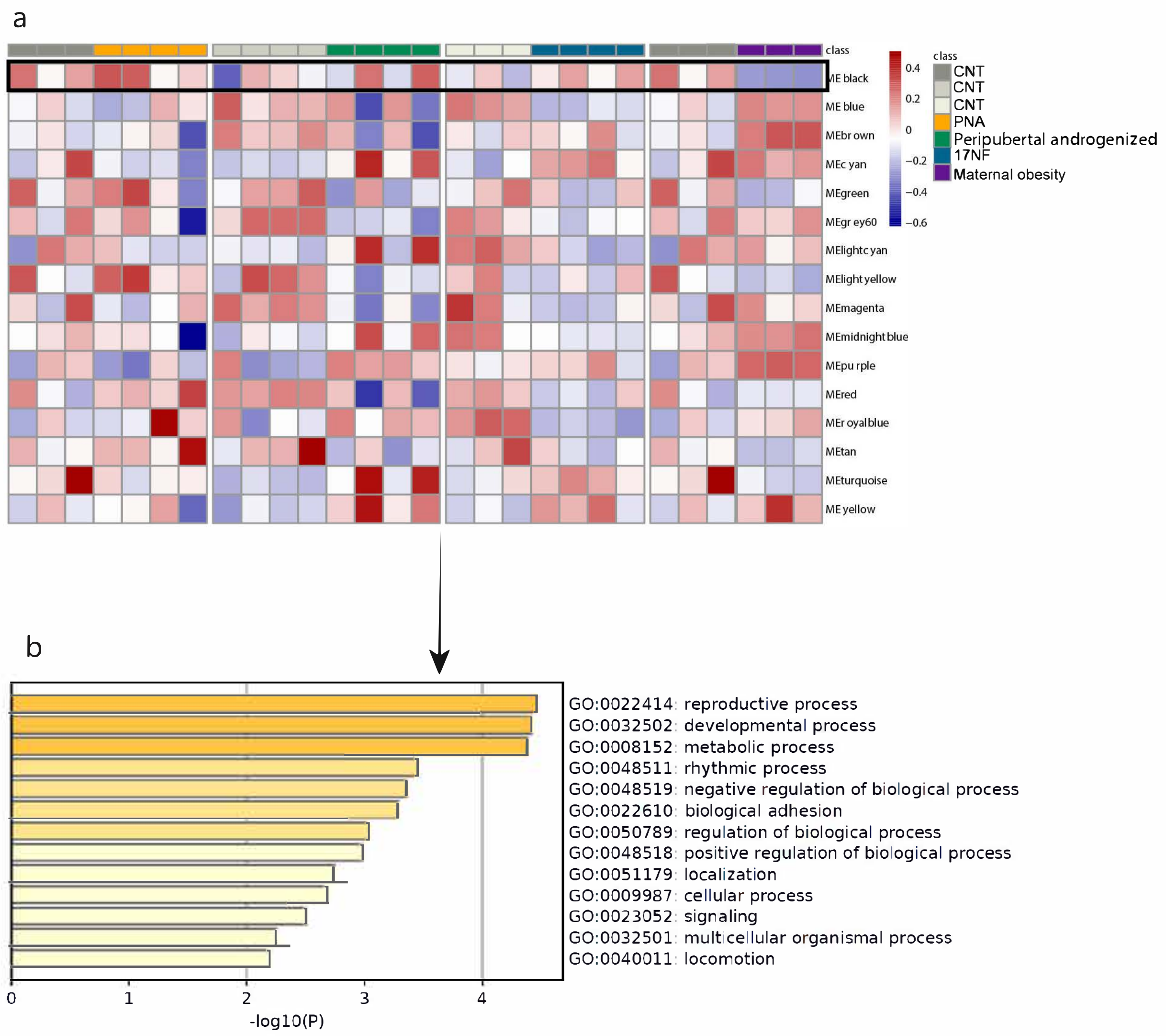

Supplementary Figure 1. (a) Heatmap depicting the correlation between module eigengenes and PNA, Peripubertal androgenized, 17NF and maternal obesity model phenotype. (b) GO enrichment analysis of selected module gene.

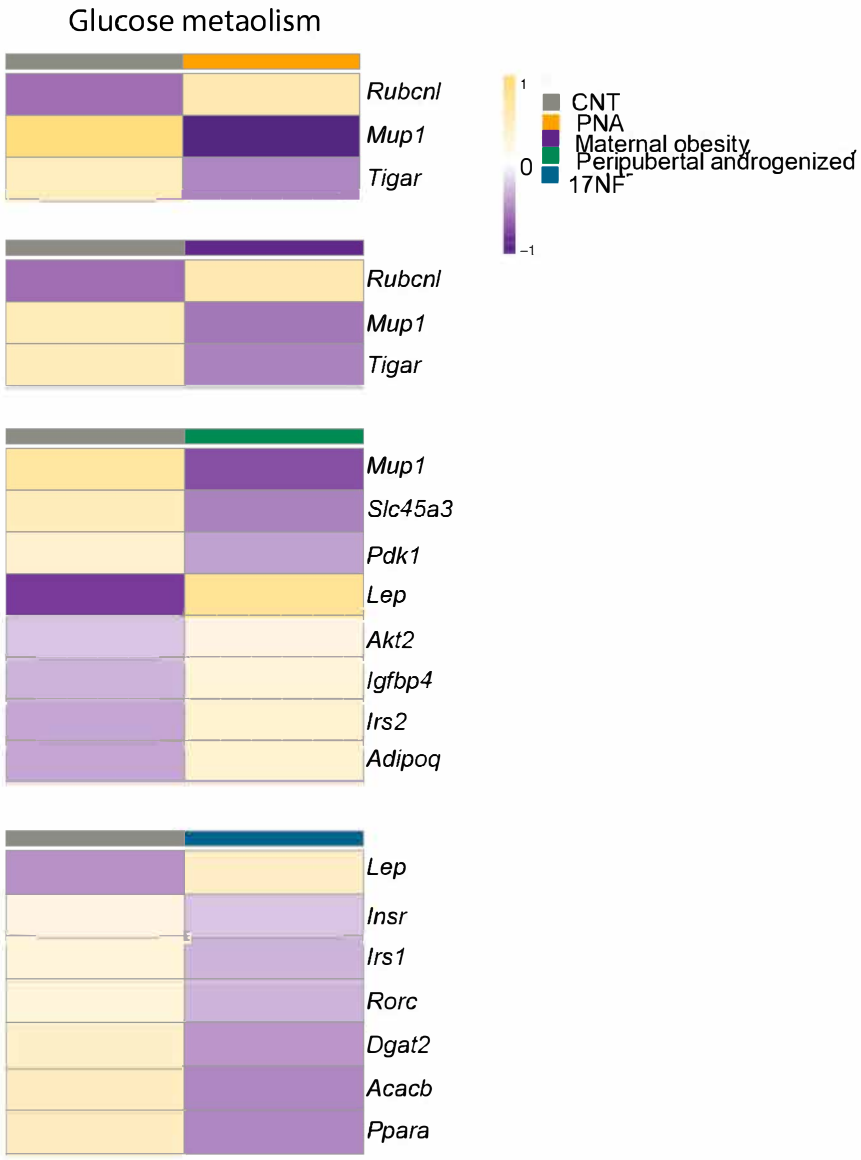

Supplementary Figure 2. Heatmap of expression of DEGs of adipose tissue enriched in glucose homeostasis in PNA, maternal obesity, Peripubertal androgenized, and 17NF, respectively.

a

Models	Hypothalamus	Ovary	MII oocytes	Adipose Tissue
**PNA**				
	Atp1b1↓			*Atp2a3*↓
	Ccl21a ↑	*Ccl21a* ↓		*Ccl5* ↑
				Chchd2-ps ↑
				Csf2rb ↑
				Fcer2a ↓
				Hspb1 ↓
				Ms4a6b ↑
	Rpl10-ps6↑			*Rpl3l* ↓
		Wfdc18↑		*Wfdc1* ↓
**Peripubeteral androgenized**				
	Atp5j ↑	*Atp1a3*↓		*Atp1a3*↓
		C1qa↑		*C1qa*↑
		C1qb↑		*C1qb*↑
		C1qc↑		*C1qc*↑
		Ccl6↑		*Ccl6*↑
		Cfp↑		*Cfp*↑
		Chchd2-ps↓		*Chchd2-ps*↑
		Csf1r ↑		*Csf1r* ↑
		Elovl5↓		*Elovl5*↓
		Fcer1g↑		*Fcer1g*↑
		Hspb1↓		*Hspb1*↓
		Ms4a7↑		*Ms4a7*↑
		Prune2↓		*Prune2*↓
		Rpl3-ps1↓		*Rpl3-ps1*↓
		Tcim↓		*Tcim* ↑
		Tkt↓		*Tkt*↓
	Wfdc18 ↓	*Wfdc17*↑		*Wfdc17*↑
**17NF**				
		Atp1a3↓		*Atp1a3*↓
		C1qa↑		*C1qa*↑
		C1qb↑	*C1qb*↑	*C1qb*↑
		C1qc↑		*C1qc*↑
		Ccl6↑		*Ccl6*↑
		Cfp↑		*Cfp*↑
		Chchd2-ps↓		*Chchd2-ps*↓
		Csf1r ↑		*Csf1r* ↑
	Elovl6↑	*Elovl5*↓	*Elovl5*↑	*Elovl5*↓
		Fcer1g↑		*Fcer1g*↑
		Hspb1↓	*Hspb1* ↑	*Hspb1*↓
		Ms4a7↑		*Ms4a7*↑
		Prune2↓		*Prune2*↓
		Rpl3-ps1↓		*Rpl3-ps1*↓
		Tcim↓		*Tcim*↓
		Tkt↓		*Tkt*↓
		Wfdc17↑		*Wfdc17*↑
**Maternal obesity**				
		Atp1a3 ↑		*Atp1a3* ↑
		Ccl4 ↑		*Ccl5* ↑
		Chchd2-ps↑		*Chchd2-ps* ↓
				Csf2rb ↑
		Elovl6↓		*Elovl5*↓
		Fcer1a ↓		*Fcer2a* ↑
		Hspb1 ↑		*Hspb1* ↓
		Ms4a4c↑		*Ms4a6b* ↑
				Prune2↓
		Rpl5-ps2↓		*Rpl5-ps1*↑
				Tkt↓
				Wfdc3↑

b

Models	Hypothalamus	Ovary	MII oocytes	Adipose Tissue
**PNA**	*Hsd3b1* ↑	*Hsd11b2* ↓	*Hsd3b1* ↑	
			Hsd17b7 ↓	
**Peripubeteral androgenized**		*Hsd17b7* ↓	*Hsd17b12* ↑	*Hsd11b1*↑
		Hsd3b1 ↓		*Hsd17b10*↑
		Hsd3b2↓		
		Hsd3b6↓		
**17NF**		*Hsd11b1*↓	*Hsd17b13* ↑	*Hsd11b1*↓
		Hsd17b12↓		*Hsd17b12*↓
		Hsd17b7↓		*Hsd17b7*↓
		Hsd3b1↓		
**Maternal obesity**	*Hsd3b1* ↓	*Hsd11b2*↓	*Hsd17b10* ↑	
			Hsd3b1 ↑	

c

Models	Hypothalamus	Ovary	MII oocytes	Adipose Tissue
**PNA**				
	Cfd↓			*Cfd*↓
		Car14↓		
	Cyp11a1↑	*Cyp2f2*↑	*Cyp11a1*↓	
	Fabp4↓	*Fabp4*↑		*Fabp3*↑
	Fam131a↓	*Fam213b*↓	*Fam221a*↓	
	Scd1↓	*Scd4*↑		
**Peripubeteral androgenized**				
		Car3↑		*Car3*↑
		Cyp27a1↑		*Cyp27a1*↑
		Cyp2d22↓	*Cyp2c29*↓	
	Fam133b↑	*Fam213b*↓	*Fam220a*↓	*Fam213b*↑
**17NF**		*Car5b*↓		*Car5b*↓
				Cfd↓
		Cyp19a1↑	*Cyp19a1*↑	
		Cyp2d22↓		*Cyp2d22*↓
				Fabp5↓
		Fam213b↓	*Fam214b*↓	*Fam111a*↑
	Scd1↑			*Scd2*↓
**Maternal obesity**				
		Car14↓		*Car14*↓
	Cyp11a1↓	*Cyp11a1*↑	*Cyp1b1*↑	*Cyp27a1*↓
		Cyp19a1↓		
	Fabp4↓			
	Fam193b↑	*Fam213b*↑	*Fam222b*↓	
	Scd1↓			*Scd3*↓

Supplementary Figure 3. Summary of specific gene sets in each mouse model identified across tissues: (a) hypothalamus, ovary, adipose tissue and MII oocytes, (b) specific genes changed in steroid hormone metabolism in each mouse model (PNA, peripubertal androgenized, 17NF and maternal obesity) and (c) specific gene expression alteration unique to each animal models.

Table S1: Differentially expressed genes (DEGs) in the hypothalamus in PNA, Peripubertal androgenized, 17NF, and maternal obesity animal models

PNA			Peripubertal Androgenized			17NF			Maternal Obesity		
Gene	Log2FoldChange	P-value	Gene	Log2FoldChange	P-value	Gene	Log2FoldChange	P-value	Gene	Log2FoldChange	P-value
Acsbg1	1,80	0,01	*A0pc11*	1,59	0,03	*Acot7*	1,69	0,04	*Acly*	-1,79	0,02
Acta1	-4,28	0,03	*Ank2*	2,97	0,05	*Elovl6*	2,81	0,04	*Adh1*	-3,92	0,00
Adig	-4,57	0,04	*Atp5j*	0,86	0,02	*Fasn*	1,62	0,00	*Adipoq*	-2,53	0,03
Agfg2	-4,50	0,05	*Calm3*	2,07	0,01	*Gm10800*	-3,12	0,05	*Cfd*	-1,68	0,00
Akr1b7	-4,05	0,04	*Ccdc6*	-3,65	0,03	*Gm11263*	2,85	0,03	*Cyp11a1*	-1,16	0,03
Akr1cl	1,92	0,00	*Cox4i1*	0,85	0,01	*Gm24447*	-1,15	0,04	*Fabp4*	-1,90	0,00
Aldoa	-0,94	0,02	*Csn2*	-3,54	0,03	*Hmgcs1*	-2,66	0,04	*Fam193b*	4,02	0,04
Atp1b1	-2,72	0,01	*Csn3*	-3,38	0,02	*Hp1bp3*	-3,23	0,03	*Fasn*	-1,60	0,02
Atp5g2	2,85	0,01	*Eif5*	-3,78	0,02	*Igfbp7*	3,95	0,03	*Fdx1*	-2,15	0,01
Atpaf1	-4,77	0,03	*Fam133b*	3,23	0,02	*Mlf2*	2,73	0,01	*Gm10925*	-0,58	0,04
Bex2	-1,44	0,05	*Gal*	-2,23	0,03	*mt-Tf*	-4,47	0,00	*Gm28437*	-0,67	0,02
Car3	-1,64	0,00	*Gfap*	3,25	0,02	*mt-Tm*	-3,27	0,00	*Gm3764*	3,43	0,05
Ccl21a	2,28	0,05	*Gm11599*	-3,60	0,04	*Pafah1b3*	3,35	0,03	*Hsd3b1*	-1,09	0,05
Cd200	-3,88	0,04	*Gm15564*	-1,35	0,03	*Pdcd5*	2,13	0,05	*Igfbp7*	-4,52	0,00
Cd74	1,69	0,02	*Gm28661*	0,67	0,04	*Rny1*	-2,78	0,00	*Lgals1*	-1,93	0,01
Cfd	-1,33	0,00	*Gpx3*	-1,55	0,00	*Rps20*	1,83	0,02	*Mfap2*	-4,33	0,02
Clu	0,91	0,03	*Inha*	2,18	0,02	*Rps25*	-1,19	0,04	*Mgst1*	-2,59	0,00
Csn1s2a	-5,49	0,01	*Lars2*	-1,10	0,00	*Scd1*	0,96	0,01	*mt-Cytb*	-0,68	0,03
Cyp11a1	0,76	0,03	*Mapk8ip3*	-3,37	0,02	*Tnnt3*	3,38	0,03	*Pcdh17*	2,40	0,03
Cyp17a1	3,12	0,02	*Mobp*	1,69	0,00	*Trappc2l*	-3,97	0,02	*Pnrc1*	-4,25	0,03
Eef1a1	1,17	0,00	*mt-Nd4l*	0,90	0,04	*Trmt112*	4,072	0,02	*Psmb10*	3,40	0,05
Fabp4	-1,37	0,01	*Mycbp2*	-3,62	0,04				*Scd1*	-1,12	0,04
Fam131a	-4,53	0,04	*Nefm*	1,94	0,04				*Selenok*	1,33	0,03
Fau	0,94	0,02	*Notch3*	-4,03	0,01				*Serinc3*	-1,71	0,04
Gja1	1,90	0,02	*Npy*	-3,19	0,03				*Sfrp4*	-1,75	0,04
Gm10132	1,35	0,02	*Nudt19*	2,707	0,030				*Stmn3*	1,17	0,03
Gm15501	2,16	0,01	*Nudt3*	-2,324	0,031				*Thrsp*	-1,71	0,03
Gm4149	0,94	0,04	*Pfkl*	2,681	0,032						
Gm8210	2,33	0,04	*Ppt1*	-3,843	0,015						
Gpm6a	-2,92	0,04	*Psmb3*	1,857	0,048						
Gstm1	1,27	0,02	*Rnf157*	-3,497	0,044						
Gstm2	2,78	0,00	*Skp1a*	2,810	0,028						
H3f3a	1,34	0,04	*Sparc*	-0,791	0,037						
Hba-a1	-1,23	0,01	*Tapbp*	-3,019	0,042						
Hbb-bs	-1,30	0,04	*Timp2*	-2,168	0,047						
Hmgcs2	2,21	0,01	*Tmem191c*	-3,019	0,042						
Hsd3b1	1,19	0,00	*U2af1*	3,137	0,027						
Hsp90b1	1,30	0,05	*Wap*	-2,600	0,041						
Inha	2,56	0,00	*Wfdc18*	-2,701	0,013						
Inhba	4,51	0,00									
Lyz2	2,20	0,00									
Malat1	0,63	0,01									
Mapk8ip3	-4,85	0,02									
Mapt	-1,30	0,04									
Mbp	-1,01	0,05									
Meg3	-1,10	0,00									
Mest	-4,78	0,03									
mt-Th	-1,77	0,01									
mt-Ts2	-1,56	0,03									
Mylpf	-5,01	0,02									
Nsmf	-5,02	0,01									
Ntrk2	-2,77	0,02									
Nudt3	-2,29	0,03									
Pcolce	2,92	0,03									
Pitpnm1	-3,07	0,01									
Pmch	-1,93	0,01									
Ptma	1,37	0,02									
Rab3a	-1,69	0,03									
Rgs2	2,56	0,04									
Rpl10-ps6	4,30	0,04									
Rpl18a	0,93	0,00									
Rpl23	0,85	0,01									
Rpl31	0,77	0,02									
Rpl39	1,71	0,00									
Rpl5	0,99	0,05									
Rpl7	1,81	0,00									
Rps13	1,30	0,05									
Rps14	0,76	0,02									
Rps15a	1,39	0,00									
Rps16	0,91	0,04									
Rps17	1,33	0,01									
Rps18	1,01	0,00									
Rps23	0,72	0,05									
Rps24	1,21	0,01									
Rps25	1,07	0,02									
Rps26	1,18	0,03									
Rps27a	1,70	0,00									
Rps28	0,99	0,05									
Rps3a1	1,28	0,02									
Rps4x	1,28	0,03									
Rps5	0,98	0,02									
Rps7	1,12	0,04									
S0p47	-1,95	0,04									
Scd1	-1,13	0,03									
Slc22a17	-2,42	0,00									
Snhg11	-1,15	0,04									
Tceal9	1,29	0,01									
Tmem176b	2,09	0,03									
Tpt1	1,01	0,03									
Trf	-5,56	0,00									
Txnip	2,40	0,03									

Table S2: DEGs involved in lipid metabolism, steroid metabolism, and gonad development biological processes in hypothalamus of PNA, Peripubertal androgenized, 17NF, and maternal obesity animal models

PNA				Peripubertal Anddrogenized				17NF				Maternal Obesity			
ID	Term	p-value	adjust.p	ID	Term	p-value	adjust.p	ID	Term	p-value	adjust.p	ID	Term	p-vaule	adjust.p
GO:0019216	Lipid metabolism	0,02	0,02	GO:0032368	Lipid metabolism	0,01	0,01	GO:0006631	Lipid metabolism	0,01	0,01	GO:0019216	Lipid metabolism	2,1E-05	0,00
GO:0019218	Regulation of steroid hormone metabolic process	0,04	0,04	GO:2000833	Regulation of steroid hormone metabolic process	0,02	0,02	GO:0009261	Nucleotide metabolism	0,02	0,02	GO:0008207	Regulation of steroid hormone metabolic process	0,00	0,00
GO:0090100	Regulation of Kinase activity	0,04	0,04	GO:0010737	Regulation of Kinase activity	0,04	0,04	GO:0006631	Carbohydrate metabolism	0,00	0,00	GO:0006957	Regulation of Kinase activity	0,01	0,01
GO:0009145	Nucleotide metabolism	0,02	0,02	GO:0009150	Nucleotide metabolism	0,00	0,00	GO:0008202	Regulation of steroid hormone metabolic process	0,00	0,00	GO:0009150	Nucleotide metabolism	0,03	0,03
GO:0006979	Response to oxidative stress	0,01	0,01	GO:0006119	Response to oxidative stress	0,01	0,01	GO:0070828	Chromatin modification	0,03	0,03	GO:0006979	Response to oxidative stress	0,00	0,00
GO:0002683	Regulation of immune systerm response	0,04	0,04	GO:0006959	Regulation of immune systerm response	0,01	0,01					GO:0002313	Regulation of immune systerm response	0,02	0,02
GO:0006631	Carbohydrate metabolism	0,02	0,02	GO:0046942	Carbohydrate metabolism	0,04	0,04					GO:0072330	Carbohydrate metabolism	0,00	0,00
GO:0034113	Cell-cell communication	0,01	0,00	GO:0086019	Cell-cell communication	0,03	0,03					GO:0098742	Cell-cell communication	0,00	0,00
GO:0008585	Gonad development	0,04	0,04	GO:0032276	Gonad development	0,01	0,01								
GO:0031055	Chromatin modification	0,03	0,03												

Table S3: DEGs analysis in the ovary in PNA, Peripubertal androgenized, 17NF, and maternal obesity animal models

PNA			Peripubertal androgenized			17NF			Maternal obesity		
Gene	Log2FoldChange	P-value	Gene	Log2FoldChange	P-value	Gene	Log2FoldChange	P-value	Gene	Log2FoldChange	P-value
1700066M21Rik	1,01	0,02	*Rps2-ps2*	-2,79	0,04	*Tubb4b-ps2*	-1,17	0,04	*Snhg6*	-0,70	0,00
Cd28	1,44	0,04	*Adhfe1*	1,58	0,00	*Il1rl2*	1,14	0,02	*Rpl7*	-0,52	0,01
Inha	-0,52	0,04	*2610203C22Rik*	-1,68	0,02	*Mettl21c*	0,92	0,05	*Tram2*	-0,79	0,03
Nppc	-2,13	0,03	*Sgk3*	0,82	0,01	*D630023F18Rik*	1,77	0,00	*Khdc1b*	-0,72	0,02
Mroh2a	1,96	0,03	*Tcf24*	1,34	0,01	*Map2*	-1,09	0,00	*Sdhaf4*	0,60	0,00
Iqca	2,80	0,02	*Ncoa2*	0,52	0,04	*Myl1*	2,59	0,00	*Cavin2*	-0,50	0,02
Cnt0p5a	1,08	0,03	*Eya1*	3,46	0,01	*Slc11a1*	1,13	0,01	*Hspe1*	-0,64	0,00
Tnnt2	-1,06	0,04	*Tram2*	1,05	0,02	*Des*	-0,74	0,02	*Kctd18*	-0,66	0,03
Lhx9	1,00	0,03	*Kcnq5*	3,05	0,00	*Acsl3*	-0,67	0,01	*Sgo2a*	-0,89	0,05
Tdrd5	-0,76	0,04	*Ptp4a1*	0,53	0,04	*Ngef*	1,30	0,01	*Fam126b*	-0,58	0,01
Astn1	-2,42	0,04	*2010300C02Rik*	2,56	0,05	*Hjurp*	0,53	0,00	*Cflar*	-0,61	0,00
Ildr2	0,60	0,01	*Aff3*	1,11	0,03	*Klhl30*	-1,52	0,01	*Bmpr2*	-0,71	0,00
3110045C21Rik	-1,03	0,04	*Mettl21c*	1,19	0,03	*Pam*	-1,08	0,00	*Eif4a-ps4*	0,83	0,00
Trp53bp2	0,62	0,04	*Tex30*	0,61	0,02	*Cnt0p5a*	1,28	0,05	*Eef1b2*	-0,53	0,00
Ccdc3	-0,92	0,01	*Tmeff2*	1,94	0,00	*Epb41l5*	-0,84	0,00	*D630023F18Rik*	0,68	0,01
Celf2	0,68	0,01	*Mars2*	-0,79	0,04	*Tmem37*	-0,64	0,01	*Rpl37a*	-0,58	0,00
Enkur	2,51	0,02	*Adam23*	1,56	0,00	*3110009E18Rik*	-0,68	0,04	*Igfbp2*	0,54	0,02
Tmem141	-0,52	0,02	*Idh1*	-0,86	0,02	*Adora1*	1,04	0,00	*Cxcr2*	-4,10	0,03
Spaca9	2,68	0,02	*Cyp27a1*	1,59	0,00	*Cyb5r1*	0,52	0,02	*Pnkd*	0,61	0,00
Cobll1	0,58	0,02	*Tuba4a*	0,85	0,01	*Ptprv*	-0,81	0,03	*Chpf*	0,53	0,02
Zfp385b	-1,05	0,04	*Des*	-1,79	0,00	*Cfh*	0,18	0,04	*Irs1*	-0,71	0,03
Ankrd63	-0,90	0,05	*Obsl1*	0,61	0,02	*Rgl1*	-0,73	0,01	*Fbxo36*	0,74	0,02
Patl2	-2,77	0,04	*Tmem198*	1,69	0,05	*Ncf2*	0,87	0,02	*Rpl19-ps1*	-0,61	0,02
Pced1a	-0,78	0,04	*Inha*	0,96	0,00	*R0sel*	1,04	0,00	*Snord82*	-1,94	0,01
Fermt1	2,48	0,02	*Acsl3*	0,61	0,05	*Nphs2*	-3,82	0,01	*Pde6d*	0,61	0,01
Cst12	-0,80	0,02	*Utp14b*	1,28	0,04	*Angptl1*	-1,14	0,00	*Efhd1*	0,54	0,00
Cst8	-1,17	0,04	*Serpine2*	0,67	0,03	*Myoc*	-2,14	0,00	*Agap1*	-0,60	0,01
Bpifb4	-1,69	0,01	*Irs1*	0,92	0,02	*Fmo2*	-1,38	0,00	*Ramp1*	0,71	0,00
N0t	-0,59	0,02	*Col4a4*	1,46	0,00	*Mettl18*	-0,89	0,02	*Hes6*	0,54	0,04
Adig	1,52	0,02	*Col4a3*	1,19	0,04	*Atp1b1*	-0,68	0,00	*Pam*	-0,62	0,01
Wisp2	0,72	0,01	*Pid1*	0,84	0,01	*Mpc2*	-0,60	0,00	*B230216N24Rik*	-1,18	0,03
Wfdc2	1,97	0,03	*Rpl19-ps1*	-0,71	0,03	*Rgs5*	-0,58	0,00	*Dsel*	-0,75	0,05
Slc38a5	-1,52	0,00	*Snord82*	-2,00	0,00	*Hsd17b7*	-2,56	0,02	*Rpl28-ps1*	-0,73	0,00
Mid1ip1	-0,60	0,04	*Cops7b*	0,50	0,03	*Fcgr3*	0,63	0,00	*R3hdm1*	-0,61	0,00
Elf4	0,86	0,01	*Nppc*	1,58	0,00	*Fcer1g*	0,68	0,00	*Rpl31-ps9*	-1,48	0,05
Rps2-ps13	-0,71	0,05	*Inpp5d*	0,98	0,02	*F11r*	-0,65	0,00	*Rab7b*	0,60	0,02
Rtl8c	-0,67	0,04	*Ugt1a6a*	-1,36	0,00	*B930036N10Rik*	-3,35	0,02	*Mfsd4a*	-1,13	0,00
Xlr3b	-1,12	0,03	*D0jb3*	1,29	0,05	*Cd48*	0,85	0,03	*Plekha6*	-1,38	0,03
Heph	-0,70	0,03	*Arl4c*	0,86	0,02	*Tma7-ps*	0,60	0,00	*Atp2b4*	-0,55	0,03
Ercc6l	1,69	0,02	*Sh3bp4*	0,66	0,03	*Slamf8*	1,74	0,02	*Ppp1r12b*	-0,79	0,00
Arxes2	-0,50	0,04	*Ackr3*	0,65	0,03	*Cadm3*	1,92	0,00	*Nr5a2*	-0,53	0,02
Plp1	-1,21	0,04	*Per2*	1,26	0,02	*Ifi213*	2,82	0,03	*Ivns1abp*	-0,76	0,00
Pak3	-0,59	0,05	*Kif1a*	1,22	0,00	*Ifi207*	1,50	0,03	*9430034N14Rik*	-1,57	0,03
Alas2	-2,05	0,01	*Pdcd1*	-1,20	0,03	*Mndal*	1,10	0,00	*Tdrd5*	-1,08	0,00
Rps12-ps7	3,28	0,04	*Pam*	-0,84	0,00	*Ifi202b*	-3,97	0,00	*Ralgps2*	-0,73	0,00
Phex	-2,97	0,01	*Zcchc2*	0,65	0,03	*Ephx1*	0,60	0,00	*Prrc2c*	-0,57	0,00
S100g	2,63	0,04	*Tmem37*	-0,92	0,01	*Rpl21-ps1*	-3,69	0,00	*Mpc2*	0,51	0,00
Rps24-ps2	0,74	0,05	*Mgat5*	0,53	0,04	*Nek2*	-1,23	0,01	*Mgst3*	0,60	0,00
Fabp4	1,00	0,00	*Thsd7b*	-2,28	0,00	*Hsd11b1*	-1,20	0,01	*Pbx1*	-0,51	0,00
Car3	1,03	0,00	*Ikbke*	1,01	0,01	*Il15ra*	-0,74	0,02	*Sh2d1b1*	2,95	0,00
Tmem212	6,39	0,00	*Slc45a3*	2,23	0,00	*Cacnb2*	0,73	0,02	*Dusp23*	0,52	0,05
Rpl32-ps	1,44	0,03	*Cdk18*	1,57	0,00	*Clic3*	-1,99	0,00	*Fcer1a*	-3,45	0,04
Trim2	0,52	0,04	*Klhdc8a*	1,93	0,01	*Ptgds*	1,07	0,00	*Ifi203*	-0,79	0,00
Lrrc71	1,44	0,03	*Etnk2*	0,76	0,01	*Fc0*	1,67	0,01	*Akt3*	-0,69	0,05
Tsacc	-0,89	0,04	*Lax1*	2,47	0,05	*Rpsa-ps9*	0,69	0,00	*Cenpf*	-0,63	0,01
Rab25	3,60	0,00	*Fmod*	1,15	0,03	*Lrrc8a*	-0,57	0,05	*Nenf*	0,76	0,00
S100a3	3,90	0,01	*Mybph*	-2,61	0,01	*Crat*	-0,70	0,00	*9430037O13Rik*	-3,99	0,03
Riiad1	2,39	0,01	*Adora1*	-1,10	0,01	*Lamc3*	-1,10	0,01	*Lpgat1*	-0,57	0,00
Rpl31-ps11	-0,56	0,05	*Ipo9*	0,53	0,00	*Aif1l*	-0,82	0,00	*Ccdc3*	-1,35	0,00
Ecm1	-0,69	0,00	*Ptprc*	0,74	0,04	*Lcn2*	-1,41	0,01	*Itih5*	-0,84	0,00
Car14	-1,39	0,00	*Rgs2*	0,70	0,00	*Slc25a25*	-0,90	0,03	*2900092N22Rik*	-1,57	0,03
Ankrd35	-0,88	0,02	*Rgs13*	3,01	0,00	*Fam102a*	-0,83	0,00	*Mllt10*	-0,58	0,02
Ovgp1	5,94	0,00	*Ivns1abp*	0,84	0,02	*Traf1*	-1,11	0,04	*Dph7*	-0,68	0,04
Henmt1	3,38	0,03	*Fam129a*	-1,09	0,00	*Hc*	1,69	0,03	*Mrpl41*	0,50	0,01
Tmem56	0,94	0,02	*Ncf2*	1,02	0,00	*Arhgap15*	0,88	0,03	*Fbxw5*	0,69	0,00
Ddit4l	-1,03	0,00	*A830008E24Rik*	3,03	0,00	*Fap*	1,48	0,03	*Bmyc*	0,64	0,00
Bmpr1b	-0,82	0,02	*Tdrd5*	1,63	0,03	*Csrnp3*	-0,74	0,01	*Rpl7a*	-0,52	0,00
Rpsa-ps10	0,72	0,01	*Axdnd1*	0,80	0,02	*Rbm45*	-1,50	0,00	*Brd3os*	0,90	0,04
Calb1	1,98	0,01	*Pappa2*	-1,81	0,00	*Olfr1033*	1,02	0,00	*Ralgds*	-0,78	0,02
Cfap206	4,48	0,01	*Serpinc1*	-1,66	0,04	*Mybpc3*	-1,79	0,00	*Ntng2*	-2,61	0,03
Ccl21a	-0,70	0,02	*Dnm3*	0,75	0,01	*Hsd17b12*	-0,55	0,00	*Kyat1*	0,78	0,04
Fam166b	5,10	0,00	*Prrx1*	-0,67	0,02	*2810002D19Rik*	0,85	0,01	*BC005624*	0,52	0,01
Arhgef39	-0,92	0,03	*Mpc2*	-0,77	0,00	*Cd44*	0,90	0,01	*Fam78a*	-0,82	0,02
Nr4a3	4,04	0,01	*Creg1*	0,71	0,00	*Dcdc5*	-5,24	0,00	*Ak1*	0,92	0,00
8030451A03Rik	-0,88	0,02	*Fam78b*	-1,33	0,02	*Kc04*	2,77	0,00	*Rpl12*	-0,78	0,00
Cdk5rap2	-0,68	0,03	*Pbx1*	0,51	0,00	*Lgr4*	-0,63	0,00	*Cntrl*	-0,57	0,03
Tex38	3,23	0,04	*Rgs5*	-0,58	0,04	*Actc1*	-2,64	0,03	*Wdr38*	-1,34	0,05
Tctex1d4	2,98	0,02	*Hsd17b7*	-2,44	0,00	*Meis2*	0,63	0,01	*Scai*	-0,63	0,03
D0li1	3,51	0,00	*Fcgr2b*	0,83	0,00	*Rasgrp1*	-1,31	0,04	*Kif5c*	-1,39	0,02
Mir692-2	1,47	0,04	*Fcgr4*	1,47	0,00	*Ankrd63*	1,17	0,03	*Prpf40a*	-0,51	0,01
Fam167b	-0,55	0,03	*Fcgr3*	1,22	0,00	*Chac1*	-2,51	0,01	*Kcnj3*	-2,37	0,00
Ubxn10	3,47	0,01	*Apoa2*	-2,22	0,03	*Mapkbp1*	0,65	0,00	*Nr4a2*	-1,14	0,02
Fhad1	3,47	0,01	*Fcer1g*	1,09	0,00	*Tmem87a*	-0,98	0,01	*Pkp4*	-0,67	0,01
Casz1	0,97	0,01	*Rpl27-ps1*	-1,12	0,05	*Tmem62*	-0,80	0,02	*5330411J11Rik*	-3,48	0,04
Mmel1	-1,01	0,01	*Ly9*	2,77	0,00	*Adal*	-0,85	0,01	*Tanc1*	-0,53	0,01
Fam213b	-0,53	0,01	*Slamf7*	2,80	0,00	*Strc*	2,80	0,04	*Baz2b*	-0,50	0,01
Slc35e2	0,60	0,01	*Cd48*	0,82	0,04	*Ell3*	-3,20	0,03	*Ppig*	-0,66	0,00
7330423F06Rik	1,29	0,00	*Cd84*	1,27	0,00	*Sord*	-0,55	0,03	*Atf2*	-0,52	0,01
Ccdc146	2,59	0,02	*Slamf8*	2,21	0,02	*Hdc*	-0,68	0,04	*Ccdc141*	-0,72	0,02
Drc1	3,15	0,03	*Cadm3*	3,50	0,00	*Ckap2l*	-0,93	0,04	*Zfp385b*	-0,99	0,04
1700001C02Rik	5,00	0,00	*Ifi204*	-0,70	0,03	*Rassf2*	-0,85	0,01	*Ube2l6*	0,53	0,04
C1qtnf7	-0,52	0,05	*Cox20*	-0,75	0,00	*Cst12*	-1,63	0,00	*Pex16*	0,64	0,00
0610040J01Rik	3,31	0,01	*Coq8a*	1,17	0,00	*Cst8*	-2,00	0,00	*Commd9*	0,64	0,00
Lnx1	-1,37	0,01	*Itpkb*	1,07	0,00	*Rps15a-ps7*	-5,15	0,00	*Cd59a*	0,61	0,00
0aa	0,65	0,04	*Ephx1*	1,64	0,00	*Mcts2*	-0,51	0,03	*Rpl10-ps1*	-0,96	0,01
Fam47e	3,48	0,01	*Rpl35a-ps2*	0,86	0,02	*Bpifb6*	-1,61	0,01	*Mpped2*	-1,07	0,01
Tmem119	-0,73	0,03	*Susd4*	1,22	0,01	*Bpifa2*	-3,96	0,00	*Emc4*	0,59	0,00
1500011B03Rik	-0,69	0,02	*Taf1a*	0,66	0,04	*Cbfa2t2*	-0,61	0,03	*Arhgap11a*	-0,66	0,04
D0h10	2,65	0,04	*Mark1*	0,54	0,02	*Acss2*	-1,37	0,00	*Thbs1*	-0,54	0,00
Cldn15	0,87	0,04	*Ptpn14*	-0,75	0,01	*Snhg11*	0,62	0,03	*Knl1*	-0,78	0,03
Pdgfa	0,82	0,01	*Atf3*	2,57	0,00	*Adig*	-1,22	0,04	*Rmdn3*	0,59	0,02
Rasl11a	-0,90	0,00	*Nmt2*	-0,71	0,00	*Mybl2*	1,12	0,04	*Stard9*	-0,65	0,04
Wnt16	2,74	0,03	*Suv39h2*	1,05	0,04	*Wisp2*	-1,27	0,00	*Ctdspl2*	-0,54	0,04
E330009J07Rik	0,89	0,03	*Bend7*	0,82	0,04	*Wfdc2*	-2,45	0,01	*Ap4e1*	-0,67	0,03
Aoc1	1,47	0,04	*Phyh*	0,77	0,00	*Wfdc3*	-1,78	0,02	*1810024B03Rik*	1,22	0,00
Mthfd2	-0,64	0,01	*Ccdc3*	0,86	0,03	*Tnnc2*	4,65	0,03	*Chchd5*	0,52	0,01
Cfap100	2,79	0,03	*Dhtkd1*	1,27	0,04	*Eya2*	-0,53	0,05	*Spef1*	0,91	0,00
Aldh1l1	-0,56	0,03	*Celf2*	0,57	0,03	*Arfgef2*	-0,87	0,00	*Ap5s1*	0,69	0,02
Tpt1-ps3	0,56	0,03	*Itih2*	5,08	0,00	*Bmp7*	-1,07	0,01	*Rpl26-ps5*	-2,69	0,01
Cidec	1,49	0,01	*Itih5*	1,23	0,04	*Pck1*	-1,53	0,00	*0a20*	0,57	0,00
Timp4	1,35	0,01	*Pfkfb3*	-0,91	0,02	*Rpl22-ps1*	-0,83	0,02	*Thbd*	0,56	0,04
Fxyd4	3,69	0,02	*Vim*	-0,62	0,00	*Ebp*	-0,64	0,00	*Zfp442*	-0,82	0,05
Il17ra	-0,63	0,04	*Hacd1*	-0,72	0,01	*Rpl3-ps1*	-1,88	0,00	*Defb19*	0,54	0,02
Mug-ps1	-3,01	0,02	*Hnmt*	2,02	0,00	*Maoa*	-0,57	0,00	*Pdrg1*	0,52	0,00
Cd27	-0,79	0,01	*Psd4*	1,27	0,03	*Maob*	-1,13	0,00	*Hck*	1,23	0,01
Pde6h	1,86	0,05	*Clic3*	-2,27	0,00	*Cfp*	0,53	0,02	*Zfp341*	1,18	0,02
Etfbkmt	-0,51	0,02	*Ptgds*	2,36	0,00	*Nsdhl*	-1,25	0,00	*Rpl5-ps2*	-1,40	0,00
Cyp2f2	3,54	0,01	*Kcnt1*	0,91	0,01	*Rpl3-ps2*	-0,57	0,00	*Edem2*	0,64	0,00
Ppp1r14a	-0,65	0,02	*Card9*	2,90	0,03	*D0se1l1*	-0,73	0,02	*Top1*	-0,54	0,00
Cox7a1	-1,02	0,01	*Adamtsl2*	0,91	0,05	*Heph*	0,52	0,00	*Lpin3*	0,62	0,04
Lin37	-0,62	0,03	*Sardh*	0,54	0,04	*Cited1*	-1,66	0,00	*Chd6*	-0,59	0,01
Rhpn2	1,41	0,01	*Brd3*	0,57	0,01	*Slc16a2*	-0,58	0,00	*Oser1*	0,55	0,02
Tulp2	-1,30	0,02	*Rxra*	0,74	0,00	*Diaph2*	0,59	0,01	*Wisp2*	0,98	0,00
Saa3	-0,67	0,03	*Ptges*	0,52	0,04	*Arxes1*	-1,19	0,00	*Ctsa*	0,56	0,00
Saa1	-1,23	0,05	*Ass1*	3,41	0,00	*Tbc1d8b*	0,62	0,05	*Pltp*	0,64	0,01
E2f8	-1,21	0,04	*Aif1l*	-0,93	0,00	*Acsl4*	-1,35	0,00	*Eya2*	-0,89	0,02
Rps12l1	1,21	0,02	*1110008P14Rik*	0,77	0,00	*Tmem164*	0,51	0,00	*Zfas1*	-0,52	0,01
Prss23	-0,65	0,01	*Lcn2*	2,50	0,00	*Fgd1*	0,60	0,04	*Cebpb*	-0,71	0,03
Ankrd42	1,24	0,02	*Eng*	-0,64	0,03	*Rpl21-ps15*	0,61	0,00	*Fam210b*	0,60	0,01
Cox20-ps	-0,93	0,02	*Rpl12*	-0,50	0,04	*Car5b*	-0,98	0,01	*Aurka*	0,55	0,05
Folr1	-0,79	0,00	*Stom*	0,57	0,03	*Piga*	0,74	0,02	*Rtf2*	0,51	0,01
Hbb-bt	-2,18	0,00	*Strbp*	-0,50	0,01	*Mid1*	-1,07	0,01	*Adrm1*	0,50	0,00
Hbb-bs	-2,02	0,00	*Nr5a1*	0,76	0,00	*Fgf2*	-0,83	0,03	*Lama5*	0,70	0,02
Sez6l2	-0,55	0,02	*Arhgap15*	-1,81	0,00	*Rpl21-ps10*	-2,88	0,00	*Rps21*	-0,70	0,00
Prss8	2,68	0,01	*Arhgap15os*	-1,15	0,01	*Postn*	1,36	0,00	*Ogfr*	0,58	0,01
Fgfr2	0,64	0,02	*Nmi*	0,59	0,04	*Mme*	1,33	0,00	*Praf2*	0,53	0,03
Caly	3,26	0,02	*Arl5a*	-0,95	0,00	*Lxn*	0,57	0,02	*Timm17b*	0,56	0,00
Urah	4,58	0,05	*Cacnb4*	2,44	0,00	*Ppm1l*	-0,64	0,00	*Rpl22-ps1*	-1,05	0,01
Plagl1	-0,62	0,04	*Cytip*	1,58	0,05	*Sfrp2*	2,76	0,00	*Kdm6a*	-0,54	0,02
Cited2	0,64	0,03	*Acvr1*	0,95	0,01	*Trim2*	-0,51	0,01	*Rpl36-ps10*	-1,33	0,01
Slc2a12	2,56	0,01	*Upp2*	0,87	0,02	*Kirrel*	-0,57	0,04	*Rpl39*	-0,68	0,00
Ctgf	-0,97	0,01	*Cd302*	0,81	0,00	*Rab25*	-4,03	0,01	*Thoc2*	-0,58	0,00
Cd24a	0,83	0,01	*Ly75*	1,50	0,03	*1500004A13Rik*	0,89	0,04	*Rps2-ps13*	0,65	0,01
Bves	0,99	0,04	*Cers6*	0,83	0,00	*Fdps*	-1,29	0,00	*Mir503*	-1,23	0,04
Lrrtm3	-3,59	0,04	*Nostrin*	-1,12	0,00	*Pmvk*	-1,25	0,00	*C430049B03Rik*	-0,80	0,01
2610028H24Rik	5,58	0,01	*Klhl23*	0,84	0,01	*S100a1*	-0,52	0,00	*Fam122b*	-0,89	0,02
Pdxk	0,87	0,04	*Dcaf17*	-0,55	0,01	*S100a4*	-1,68	0,01	*Smim10l2a*	-1,86	0,01
Misp	3,78	0,03	*Metap1d*	0,57	0,00	*Tchh*	-2,73	0,01	*Ints6l*	-0,52	0,03
Izumo4	-0,73	0,02	*Rapgef4*	0,81	0,04	*Cgn*	-0,80	0,01	*1110012L19Rik*	0,56	0,01
Pmch	-2,36	0,01	*Hspe1-ps5*	-1,99	0,00	*Ctss*	0,64	0,00	*Rpl30-ps10*	-1,55	0,00
Plxnc1	-0,80	0,02	*Ola1*	-0,63	0,00	*Hist2h2be*	-1,12	0,00	*Dkc1*	-0,50	0,01
Mgat4c	-1,15	0,02	*Zfp385b*	1,72	0,00	*Hist2h3c2*	-1,24	0,01	*Vbp1*	-0,61	0,00
Lgr5	0,73	0,02	*Pde1a*	-1,02	0,00	*Hist2h2aa2*	-0,95	0,00	*Tbl1x*	-0,55	0,00
Apof	-1,60	0,03	*A330069E16Rik*	-0,74	0,03	*Fcgr1*	1,09	0,04	*Dmd*	-0,59	0,01
Retn	0,95	0,02	*Pacsin3*	0,71	0,02	*Mov10*	0,60	0,02	*Arx*	-0,61	0,04
Lpl	0,53	0,02	*Lrp4*	1,26	0,00	*Kc02*	2,96	0,01	*Pola1*	-0,58	0,03
Hmox1	-0,67	0,00	*4631405J19Rik*	-2,14	0,01	*Slc6a17*	-1,86	0,01	*Heph*	-1,05	0,00
Rasd2	1,34	0,04	*Cd82*	0,55	0,00	*5330417C22Rik*	-3,05	0,00	*Rpl17-ps8*	-0,97	0,00
Ces1f	2,95	0,02	*Ldlrad3*	1,00	0,01	*Dpyd*	-0,61	0,04	*Ar*	-0,62	0,00
Mt3	-0,73	0,05	*Ehf*	1,09	0,03	*Tmem56*	-2,02	0,00	*Il2rg*	0,63	0,03
Ccdc113	4,66	0,00	*Ccdc73*	-1,14	0,01	*Slc44a3*	-0,75	0,02	*Taf1*	-0,73	0,00
Plekhg4	0,84	0,01	*Rpl10-ps1*	-1,14	0,00	*Synpo2*	-0,87	0,04	*Rps4x*	-0,56	0,00
Hsd11b2	-0,81	0,01	*Mpped2*	0,97	0,02	*Slc39a8*	-0,79	0,04	*Tsix*	-4,72	0,02
Cdh1	1,64	0,00	*Bdnf*	-1,54	0,01	*Adh1*	0,57	0,00	*Xist*	-0,59	0,00
Hp	0,93	0,01	*Lgr4*	-1,01	0,00	*Pdlim5*	-0,68	0,00	*Nexmif*	-1,19	0,00
Vac14	-0,72	0,02	*Actc1*	-3,65	0,01	*Rpsa-ps10*	0,93	0,00	*Diaph2*	-0,61	0,02
Dynlrb2	6,34	0,00	*Nusap1*	-0,63	0,03	*AI838599*	-1,32	0,02	*Rpl36a*	-0,60	0,00
Rps12-ps9	0,75	0,02	*Myef2*	0,62	0,02	*Mos*	-0,85	0,02	*Armcx5*	-1,16	0,00
Agt	-0,63	0,01	*1500011K16Rik*	-0,72	0,00	*Spink4*	0,83	0,02	*Arxes2*	-0,67	0,00
Acox2	4,75	0,00	*Mertk*	1,25	0,00	*Xpa*	-0,57	0,04	*Psmd10*	0,70	0,00
Sntn	6,16	0,01	*Fbln7*	0,74	0,05	*Trim14*	1,72	0,00	*Pak3*	-0,82	0,01
Kcnma1	0,60	0,02	*Sirpa*	1,42	0,00	*Rps15a-ps8*	-1,55	0,03	*Rpl21-ps15*	-0,72	0,00

Table S4: GO enrichment analysis of biological processes on DEGs in ovary of PNA, Peripubertal androgenized , 17NF, and maternal obesity animal models

PNA				Peripubertal androgenized				17NF				Maternal obesity			
ID	Term	p-value	adjust,p	ID	Term	p-value	adjust,p	ID	Term	p-value	adjust,p	ID	Term	p-value	adjust.p
GO:0006631	Lipid metabolism	0,00	0,00	GO:0019216	Lipid metabolism	8,41E-09	8,41E-09	GO:0006631	Lipid metabolism	1,04E-06	1,04E-06	GO:0006631	Lipid metabolism	0,01	0,01
GO:0022407	Regulation of cell cell adhesion	0,00	0,00	GO:0002683	Regulation of inflammatory response	0,00	0,00	GO:0006694	Regulation of steroid hormone metabolic process	7,75E-17	7,75E-17	GO:0016570	Histone modification	0,00	0,00
GO:0070371	ERK1 and ERK2 cascade	0,00	0,00	GO:0070371	ERK1 and ERK2 cascade	0,00	0,00	GO:0070371	ERK1 and ERK2 cascade	2,27E-06	2,27E-06	GO:0006109	Lipid metabolism	0,03	0,03
GO:0042698	Ovulation cycle	0,00	0,00	GO:0022407	Regulation of cell cell adhesion	0,00	0,00	GO:0045785	Regulation of cell cell adhesion	0,00	0,00	GO:0048638	Developmental maturation	0,00	0,00
GO:0001890	Placenta development	0,01	0,01	GO:0008202	Regulation of steroid hormone metabolic process	0,00	2,5E-05	GO:0043087	Regulation of GTPase activity	6,26E-05	6,26E-05	GO:0050727	Regulation of inflammatory response	0,00	0,00
GO:0001541	Ovarian follicle development	0,00	0,00	GO:0021700	Developmental maturation	0,00	0,00	GO:0046325	Glucose homeostasis	0,04	0,04	GO:0050796	Response to insulin	0,05	0,05
GO:0007281	Gonad development	0,01	0,01	GO:0042593	Glucose homeostasis	0,00	0,00	GO:0008585	Gonad development	0,03	0,03	GO:0008585	Gonad development	0,00	0,00
GO:1904659	Glucose homeostasis	0,03	0,03	GO:0008406	Gonad development	0,01	0,01	GO:0002866	Regulation of inflammatory response	0,00	0,00	GO:0022407	Regulation of cell cell adhesion	0,04	0,04
GO:0002437	Regulation of inflammatory response	0,03	0,03	GO:0001541	Ovarian follicle development	0,00	0,00	GO:0046628	Response to insulin	0,05	0,05	GO:0010906	Glucose homeostasis	0,04	0,04
GO:0032868	Response to insulin	0,03	0,03	GO:0016575	Histone modification	0,03	0,03	GO:0030728	Ovulation cycle	0,01	0,01	GO:0008202	Regulation of steroid hormone metabolic process	0,00	0,00
GO:0021700	Developmental maturation	0,02	0,02	GO:0042711	Maternal behavior	0,04	0,04					GO:0042698	Ovulation cycle	0,01	0,01
GO:2000831	Regulation of steroid hormone metabolic process	0,02	0,02	GO:1901142	Response to insulin	0,00	0,00					GO:0007264	Regulation of GTPase activity	0,02	0,02
				GO:0043547	Regulation of GTPase activity	0,00	0,00								
				GO:0042698	Ovulation cycle	0,01	0,01								

Table S5: Weighted gene co-expression network analysis (WGCNA) in PNA, Peripubertal androgenized, 17NF, and maternal obesity animal models.

1110003F10Rik	*Me2*
1700022I11Rik	*Mex3a*
1700109K24Rik	*Mex3c*
2610037D02Rik	*Mier1*
2810474O19Rik	*Mir1195*
4930488L21Rik	*Mir181b-2*
4930589O11Rik	*Mir1898*
4930596I21Rik	*Mir301*
4933413J09Rik	*Mir450b*
5031410I06Rik	*Mir99ahg*
5430434F05Rik	*Mndal*
6030400A10Rik	*Mycl*
6720427I07Rik	*Myo6*
9430037O13Rik	*Neb*
9430062P05Rik	*Nek10*
9530013L04Rik	*Nexmif*
A430027C01Rik	*Nlrp14*
A830082K12Rik	*Nlrp4f*
Abca8a	*Nlrp5*
Abi3bp	*Ntn4*
AC157566.3	*Ntng1*
AC166832.3	*Oosp1*
Acap2	*Pcdhga4*
Acsm3	*Pdcd4*
Akap9	*Pdpr*
Ank2	*Per3*
Ankrd12	*Phf13*
Ano4	*Pla2g2d*
Ar	*Plcd4*
Arhgap29	*Ppp1r12b*
Arid4b	*Ptbp2*
Atf7ip2	*Ptch1*
Atp2b3	*Purb*
Atrnl1	*Qk*
Atrx	*R0sel*
AW822252	*Rest*
Basp1	*Rev3l*
BB365896	*Rgs17*
Bcl11a	*Rgs2*
BE692007	*Rhpn2*
Bhlhe41	*Rn7sk*
Bmpr2	*Rnf213*
Bnc2	*Rock1*
Brd3	*Rpl10-ps2*
C130083A15Rik	*Rpl21-ps15*
C430049B03Rik	*Rpl21-ps8*
C86187	*Rpl7*
Camk2n1	*Rps19-ps7*
Cbx3-ps6	*Samd7*
Cc2d2a	*Scaf11*
Ccdc40	*Scai*
Ccdc88a	*Sclt1*
Ccl24	*Scml4*
Ccl28	*Selenop*
Ccr2	*Sema3g*
Cd5l	*Sf1*
Celf2	*Slc6a15*
Cenpe	*Slco1a4*
Cep83	*Slf2*
Chp2	*Smc2*
Chrdl1	*Smc6*
Clec1a	*Snora21*
Clk1	*Snord42b*
Cnnm1	*Sowaha*
Cobl	*Sox4*
Col11a1	*Speer4a*
Crebrf	*Sstr3*
D0jb14	*Syt16*
D630044L22Rik	*Sytl4*
D830026I12Rik	*Tcstv1*
Dbt	*Tef*
Dcn	*Tenm4*
Ddhd1	*Tet1*
Ddi1	*Tex15*
Diaph2	*Tiparp*
Dpysl2	*Tmeff1*
Dtwd2	*Tmigd1*
Dy0p	*Tnik*
Ear2	*Tnrc6b*
Ecm2	*Trim75*
Egr1	*Ubn2*
Eif4e3	*Vmn2r15*
Espn	*Wfdc3*
Esr2	*Wfdc6b*
Fam126b	*Xist*
Fam13a	*Zbtb20*
Fam196a	*Zc3h12b*
Fam81a	*Zcchc11*
Fbn2	*Zeb1*
Fcho2	*Zfhx3*
Frem1	*Zfp125*
Fubp1	*Zfp292*
Hipk2	*Zfp957*
Hist1h2ab	*Kcns2*
Hist1h3e	*Kctd12*
Hist1h4a	*Kdm5a*
Hmgcll1	*Kdm7a*
Ica1l	*Kif19a*
Ids	*Kitl*
Ifi203	*Klhl29*
Ikzf2	*Lancl3*
Ints6l	*Lig1*
Irf2bp2	*Lipa*
Itga4	*Lrrc7*
Itih5	*Luc7l2*
Jak1	*Luc7l3*
Jmjd1c	*Kcnq1ot1*
Kcnd2	

Table S6: Unique and overlapped genes are presented in the Venn diagram in PNA, Peripubertal androgenized, 17NF, and maternal obesity animal models

PNA			Peripubertal androgenized			17NF			Maternal Obesity		
Gene	Log2FoldChange	P-value	Gene	Log2FoldChange	P-value	Gene	Log2FoldChange	P-value	Gene	Log2FoldChange	P-value
Dnah7b	0,82	0,04	Lhx2	-0,66	0,00	Prim2	0,56	0,00	Serpinb12	-1,27	0,01
Usp37	-0,36	0,01	Egfl6	-0,24	0,04	Bai3	1,12	0,00	Mapkapk2	3,37	0,04
Mitd1	0,39	0,01	Tcl1b5	-0,18	0,03	Cflar	0,75	0,02	Arfgef1	-0,28	0,05
Tbc1d8	0,41	0,01	Spg7	-0,68	0,05	Syt14	1,36	0,01	Mitd1	0,47	0,00
Ankrd44	0,40	0,04	Rpa1	0,18	0,04	Pld5	2,19	0,01	Mrps9	0,59	0,04
Raph1	0,69	0,02	Fmr1	-0,35	0,03	1700112H15Rik	1,98	0,00	9430016H08Rik	3,11	0,01
Armc9	3,03	0,02	Luzp1	0,37	0,00	Lactb2	1,93	0,00	Hdlbp	-0,51	0,00
Lrrfip1	-0,38	0,04	Ube2c	-0,39	0,00	A830018L16Rik	1,10	0,04	Pam	1,40	0,02
Hdlbp	-0,52	0,01	Snd1	0,20	0,04	Lonrf2	-0,84	0,00	R3hdm1	-0,44	0,01
R3hdm1	-0,45	0,03	Sost	0,91	0,02	Dnah7b	-0,44	0,03	Nfasc	2,36	0,04
Rassf5	0,36	0,02	Jup	0,72	0,01	Pard3b	-1,02	0,03	Ipo9	1,20	0,02
Lgr6	-1,36	0,04	Eef1e1	-0,21	0,02	Spag16	1,20	0,00	2810025M15Rik	1,25	0,01
Abl2	0,33	0,01	Capns1	0,69	0,02	Xrcc5	0,80	0,00	Uck2	-1,23	0,00
Rfwd2	0,56	0,02	Nup214	0,12	0,03	Sh3bp4	-1,19	0,00	Mgst3	0,29	0,03
Uck2	-1,01	0,00	Mdp1	0,95	0,01	Rab3gap1	-0,35	0,03	Pbx1	4,49	0,00
Pbx1	4,12	0,02	Braf	-0,34	0,02	Camsap2	-0,50	0,00	Cdc42bpa	-0,29	0,02
Fmn2	0,35	0,04	Tsc2	0,71	0,01	Cdc73	-0,82	0,00	Fbxo28	0,45	0,00
Cdc42bpa	-0,35	0,03	Plekha3	-0,16	0,04	Stx6	-0,85	0,00	Spata17	-1,94	0,00
Fbxo28	0,45	0,02	Top3a	0,30	0,04	Rabgap1l	-0,37	0,03	Cenpf	-0,42	0,01
Ly96	2,86	0,05	Cd36	0,15	0,03	Rgs7	1,97	0,01	Rpl7	2,60	0,03
Tram2	-0,72	0,02	Cyp2c29	-0,44	0,05	Smyd3	0,40	0,04	Tfap2b	0,48	0,04
Khdc1a	0,72	0,01	Pafah1b2	0,39	0,01	Ptpn14	-1,77	0,03	Coa5	0,81	0,04
Mrpl30	1,22	0,04	Ap1m2	0,25	0,02	Tgfb2	0,59	0,00	Mrpl30	1,43	0,01
Bivm	0,68	0,02	Map3k11	0,88	0,01	Atp6v1h	1,30	0,00	Bivm	0,70	0,01
Ormdl1	-0,55	0,04	Pde1c	0,26	0,05	St18	-1,32	0,00	Stk17b	3,41	0,03
Casp8	-0,72	0,04	Rab11a	0,20	0,01	Adhfe1	1,20	0,00	Aox4	1,74	0,02
Trak2	1,35	0,03	Sgta	0,35	0,04	Arfgef1	-0,41	0,00	Fzd7	1,19	0,01
Als2	1,17	0,01	Cstb	-0,81	0,04	Prex2	4,55	0,00	Sumo1	0,40	0,03
Fzd7	1,31	0,01	Ufd1	0,40	0,01	Ncoa2	-0,39	0,00	Nop58	-0,43	0,00
Nop58	-0,54	0,00	Crbn	0,26	0,01	Slco5a1	1,09	0,01	Creb1	1,60	0,04
Fam117b	0,37	0,03	Kbtbd4	0,28	0,01	Eya1	0,46	0,00	Akr1cl	1,52	0,00
Ctla4	-2,79	0,00	Ctns	0,32	0,04	Tceb1	1,34	0,00	Idh1	0,88	0,02
Ccnyl1	0,41	0,05	Crkl	-0,27	0,02	Sbspon	1,55	0,02	Tmem169	-1,04	0,01
Ncl	-0,58	0,00	Cbfa2t3	-0,45	0,01	Col19a1	-0,58	0,04	Dnpep	2,06	0,01
Atg4b	-0,81	0,02	Cdt1	0,23	0,02	4931428L18Rik	0,64	0,00	Inha	4,11	0,01
Zcchc2	0,51	0,04	Usp19	-0,13	0,02	Fam178b	-0,68	0,05	Acsl3	0,35	0,01
Vps4b	-0,82	0,04	Cdc42	0,19	0,00	Inpp4a	1,30	0,00	C130026I21Rik	-1,59	0,02
Slc45a3	0,39	0,00	Eif1b	-0,30	0,01	Tmem131	-0,61	0,00	Cab39	0,75	0,00
Zc3h11a	-0,55	0,02	Homer1	-0,40	0,00	Mitd1	0,60	0,00	Itm2c	3,29	0,02
Snrpe	0,37	0,02	Khsrp	-0,31	0,01	Aff3	-1,71	0,00	Ncl	-0,70	0,00
Arpc5	-0,97	0,03	Rplp1	-0,50	0,00	Tbc1d8	-0,49	0,00	Eif4e2	1,54	0,04
Mettl18	2,37	0,02	Ap2s1	-0,30	0,02	Il1r1	1,08	0,00	Ramp1	3,15	0,01
Selp	-0,51	0,01	Herpud2	-0,13	0,03	Map4k4	-1,13	0,00	Pdcd1	5,18	0,01
Aldh9a1	-0,32	0,04	Rdh13	-0,88	0,03	Ankrd44	-0,45	0,00	Snrpe	0,33	0,04
Hsd17b7	-0,49	0,01	Arpc5	0,34	0,05	Ndufb3	0,94	0,00	Nr5a2	-0,67	0,05
Wdr26	0,39	0,01	Rps15a	-0,29	0,03	Nif3l1	1,66	0,00	Ptgs2	2,63	0,03
Brox	0,42	0,03	Dynll1	-0,34	0,01	Tmem237	0,99	0,00	Cep350	-0,76	0,02
Mia3	-0,34	0,05	Slc16a12	-0,53	0,00	Stradb	-0,29	0,03	Tdrd5	-0,80	0,00
Taf1a	-0,42	0,00	Cdk9	0,54	0,01	Plekhm3	-1,03	0,00	7420461P10Rik	-0,60	0,00
Eprs	-0,35	0,04	Ubp1	-0,14	0,03	Rpe	0,90	0,00	Ccdc181	-0,76	0,02
Slc30a1	0,74	0,03	Ick	0,26	0,03	Vwc2l	0,67	0,05	Aldh9a1	-0,39	0,01
Ttc17	-0,96	0,00	Cox4i2	-0,86	0,05	Bard1	-0,62	0,00	Sccpdh	3,99	0,00
Immp1l	0,41	0,01	Phlda2	-0,40	0,03	Smarcal1	-0,74	0,00	Cnih4	-0,49	0,05
Ttll11	-1,08	0,02	Zfp296	-0,21	0,04	Mar_4	-0,97	0,04	Capn2	3,81	0,00
Baz2b	-0,37	0,01	Ccnt1	0,40	0,00	Sgpp2	-1,13	0,01	Taf1a	-0,47	0,00
Agps	0,45	0,01	Tmem175	0,20	0,05	Serpine2	0,33	0,01	Eprs	-0,42	0,01
Tnks1bp1	0,75	0,00	Rnf170	0,19	0,02	Trip12	-0,26	0,01	Zeb2	3,18	0,02
Tmem62	1,82	0,04	Chp1	-0,16	0,02	Pid1	-0,50	0,01	Slc30a4	-0,44	0,04
Usp8	-0,79	0,00	Ndufa2	-0,64	0,01	Sp110	0,45	0,00	Rpn2	1,62	0,00
Stard7	0,29	0,03	Pam16	-0,23	0,03	Psmd1	0,46	0,00	Phactr3	-0,80	0,05
Slx4ip	-0,55	0,03	Cox6c	-0,32	0,04	Agap1	-0,71	0,00	Mllt10	-0,24	0,01
Raly	1,19	0,04	Rnf166	-0,46	0,05	Lrrfip1	-0,24	0,03	Edf1	2,02	0,04
Bcas1	-1,18	0,04	Srp19	-0,21	0,01	Col6a3	-0,38	0,01	Hc	-3,03	0,01
Dclre1c	-1,01	0,01	Mrps25	-0,20	0,04	Hdac4	-0,46	0,03	Ndufa8	0,89	0,03
Thnsl1	-0,78	0,03	Ptgds	-0,62	0,02	D1Ertd622e	-0,58	0,00	Neb	-0,73	0,02
Mastl	-0,27	0,02	Slc25a13	0,33	0,01	Pign	0,36	0,03	Itgb6	1,58	0,04
Prpf40a	-0,35	0,03	Psma2	-0,19	0,04	Daf2	1,29	0,00	Ttc21b	1,39	0,01
07-Mar	-0,31	0,03	Fnta	-0,25	0,01	Rassf5	-0,87	0,00	Ttn	-0,81	0,02
Nckap1	0,38	0,03	Nelfcd	0,27	0,02	Tmcc2	-0,60	0,00	Celf1	-0,56	0,04
Traf6	3,13	0,05	Prelid3b	0,21	0,04	Klhl12	-0,85	0,00	Ptprj	-0,94	0,05
Fbxo3	-1,01	0,02	Ube2a	-0,36	0,01	Nav1	-1,18	0,00	Ehf	-0,54	0,00
Eif2ak4	0,39	0,01	Ppdpf	-0,48	0,01	Swt1	0,26	0,01	Emc7	1,27	0,02
Bahd1	0,94	0,01	Plgrkt	0,55	0,02	Rgl1	0,71	0,01	Aqr	-0,71	0,02
Mapkbp1	0,66	0,02	Foxred2	-0,24	0,03	Lamc1	-0,73	0,00	Tmem62	1,83	0,01
Ehd4	-0,46	0,02	Eif3d	-0,26	0,05	Xpr1	-0,86	0,00	Usp8	-0,67	0,02
Zscan29	0,96	0,04	H3f3b	-0,12	0,05	Ralgps2	-0,34	0,00	Itpripl1	5,06	0,00
Mfap1b	-0,81	0,01	Birc5	-0,24	0,02	Abl2	-0,91	0,00	Rnf24	-0,61	0,01
Ncaph	-0,70	0,03	Slc12a7	-0,27	0,04	Astn1	1,58	0,05	Slx4ip	-0,85	0,00
Snrpb	3,01	0,03	Cox7c	-0,60	0,01	Rfwd2	-0,94	0,00	Kif16b	-0,62	0,03
Nop56	-0,63	0,02	Mybl2	-0,22	0,04	Rc3h1	-0,86	0,00	Chd6	-0,49	0,00
Cdc25b	-1,75	0,01	Baiap2l2	-0,44	0,04	Suco	-0,51	0,00	Rpp38	-0,54	0,05
Snrpb2	0,29	0,02	Pfn1	0,34	0,02	Nme7	0,88	0,00	Mrpl41	2,43	0,03
Insm1	1,26	0,00	Ksr1	-0,42	0,00	Pou2f1	-0,97	0,00	Ssna1	0,43	0,02
Trp53inp2	0,51	0,02	Myo1b	0,27	0,01	Mgst3	0,39	0,00	Anapc2	1,50	0,00
Trpc4ap	0,57	0,04	Ccdc170	0,35	0,02	Atf6	-0,64	0,00	Ubac1	-0,71	0,03
Wfdc15a	1,58	0,00	Frk	0,53	0,00	Copa	-0,42	0,01	Dnlz	2,46	0,02
Zfp334	0,58	0,05	Clvs2	-0,15	0,03	Cnst	-0,42	0,00	Setx	-0,77	0,01
Cstf1	-0,79	0,04	Cd164	-0,11	0,04	Acbd3	-0,39	0,00	Set	1,08	0,00
Pmepa1	3,30	0,01	Arfgef3	0,24	0,04	Enah	-0,53	0,00	Tor1b	1,92	0,02
Ppp1r3d	0,55	0,01	Vta1	0,19	0,01	Fbxo28	-0,45	0,00	Hspa5	-0,58	0,02
Lsm14b	0,87	0,00	Tmpo	-0,24	0,01	Rab3gap2	-0,49	0,00	Nr5a1	2,99	0,04
Pcmtd2	1,57	0,04	Fgd6	0,19	0,00	Cenpf	-0,29	0,01	Tnfaip6	4,79	0,00
Zfhx4	1,33	0,02	Cry1	-0,14	0,00	Kctd3	-0,55	0,00	Arl5a	1,40	0,04
Tnik	-0,88	0,01	Sirt1	-0,23	0,05	Lpgat1	-0,76	0,00	Stam2	1,19	0,02
Unc5c	0,41	0,03	Plek	0,34	0,02	Batf3	-1,36	0,00	Prpf40a	-0,34	0,01
Cenpe	-0,33	0,05	Uqcr11	-0,72	0,01	Kcnh1	-0,71	0,03	Dync1i2	-0,31	0,03
Sec62	0,33	0,02	Yeats4	0,36	0,00	Plxna2	-0,57	0,01	Atp5g3	1,87	0,02
Foxo1	0,31	0,00	Txnrd1	-0,21	0,02	Mrpl15	-1,38	0,00	Frzb	-2,58	0,05
Fam160a1	-0,54	0,02	D10Wsu102e	0,32	0,00	Lypla1	0,78	0,00	Slc39a13	2,78	0,04
Lrba	1,12	0,02	1700093K21Rik	0,74	0,02	Tcea1	0,45	0,00	Traf6	2,55	0,05
Dclk2	0,33	0,05	Chac2	0,24	0,04	Oprk1	2,75	0,02	Fam98b	-0,71	0,00
Ash1l	-0,77	0,02	Erlec1	-0,64	0,04	Rb1cc1	-0,66	0,00	Thbs1	3,13	0,05
E2f5	0,44	0,01	Clk4	0,46	0,03	Rrs1	-5,91	0,00	Mapkbp1	0,66	0,01
Samd7	-1,07	0,01	Cdkn2aipnl	0,28	0,00	Vcpip1	-0,76	0,00	Zscan29	0,85	0,02
Fxr1	-0,54	0,01	Med7	0,23	0,05	Snhg6	1,44	0,00	Mfap1b	-0,89	0,00
Plk4	-0,27	0,05	4921536K21Rik	0,60	0,01	Cops5	1,03	0,00	B2m	1,25	0,01
Exosc8	0,33	0,05	Rnf185	0,13	0,04	Tram1	-0,36	0,00	Slc27a2	-2,74	0,01
Siah2	0,46	0,00	Mfap3	0,16	0,02	Trpa1	0,81	0,00	Trpm7	-0,71	0,01
Pygo2	1,34	0,01	Twistnb	0,51	0,00	Terf1	-0,43	0,02	Ncaph	-0,61	0,03
Txnip	0,27	0,03	Map2k6	0,66	0,04	Defb41	1,56	0,04	Bub1	-0,61	0,00
Chd1l	-1,72	0,00	Cmpk2	0,25	0,01	Tfap2b	-0,64	0,02	Chchd5	2,06	0,04
Hsd3b1	4,78	0,00	Mboat2	0,19	0,03	Mcm3	-0,79	0,00	Snrpb	2,98	0,01
Aknad1	-0,85	0,00	Ddx42	0,32	0,03	Il17f	2,73	0,00	Idh3b	4,52	0,02
Dnttip2	-0,34	0,02	Itgb4	-0,52	0,04	Tram2	0,65	0,00	Nop56	-0,64	0,00
Ints12	-0,51	0,00	Luc7l3	-0,16	0,04	Tmem14a	0,40	0,04	Vps16	2,69	0,01
Usp33	-0,43	0,05	Dvl2	-0,34	0,02	B3gat2	1,18	0,02	Snrpb2	0,39	0,00
Inpp5b	-0,85	0,00	Tmem107	-0,29	0,00	Fam135a	0,72	0,00	Mgme1	-1,47	0,01
Map3k7	1,30	0,01	Krt12	-0,36	0,02	1110058L19Rik	0,85	0,00	Cst3	2,20	0,02
Dcaf10	1,30	0,00	Eftud2	0,28	0,04	Lmbrd1	1,70	0,00	Bpifa6	-3,04	0,03
Ikbkap	-0,98	0,05	Fkbp3	-0,43	0,04	4931408C20Rik	-3,77	0,00	Adnp	2,96	0,04
Ccdc171	-0,48	0,03	Sav1	-0,20	0,05	Phf3	-0,56	0,00	Pmepa1	3,57	0,02
Nfia	1,17	0,04	Timm9	-0,28	0,00	Rab23	-0,67	0,00	Ctsz	4,90	0,00
Pik3r3	-0,48	0,03	Papola	-0,15	0,04	AA619741	0,46	0,01	Lsm14b	0,52	0,03
Csmd2	4,06	0,00	Pigh	-0,59	0,02	Fam168b	-0,97	0,00	Pcmtd2	1,90	0,02
Phc2	0,38	0,02	Pcnx	0,20	0,02	Hs6st1	-1,09	0,00	Kcnmb2	0,81	0,04
Arid1a	0,25	0,03	Esyt2	-0,15	0,02	Cox5b	0,87	0,00	Ccrn4l	0,37	0,04
Alpl	4,01	0,01	Trip11	0,20	0,03	Unc50	0,30	0,01	Plch1	3,22	0,04
Fbxo42	0,39	0,05	Pitrm1	0,29	0,04	Tsga10	0,59	0,00	D930015E06Rik	-0,43	0,01
Prdm16	-0,67	0,04	Tmem14c	-0,23	0,02	Lipt1	0,39	0,02	Lrba	1,08	0,01
Esrp1	0,32	0,05	Fbp2	-0,35	0,02	Txndc9	0,83	0,00	Ubap2l	-0,64	0,00
Decr1	4,71	0,04	Nsd1	0,23	0,01	Pdcl3	1,24	0,00	Rprd2	-0,52	0,02
Usp45	0,85	0,03	Pcbd2	-0,47	0,04	Cnot11	-0,79	0,00	Notch2	1,31	0,01
Pnisr	-0,46	0,02	Cxcl14	-1,34	0,01	Rnf149	-1,62	0,00	Fnbp1l	-0,39	0,00
Topors	-0,70	0,02	Atg10	0,46	0,00	Slc9a4	0,67	0,03	Sgms2	1,56	0,03
Nudt2	0,95	0,03	Elovl7	0,25	0,01	Mfsd9	-0,54	0,01	Manba	4,35	0,00
Rnf38	0,20	0,04	Rnf180	0,39	0,04	Tgfbrap1	-0,96	0,00	Rap1gds1	0,63	0,01
Tmem38b	0,58	0,01	Ngly1	0,14	0,02	Tex30	2,32	0,00	St6galnac3	1,09	0,01
Tle1	1,41	0,01	Camk2g	0,27	0,00	Kdelc1	0,70	0,00	Hps3	-0,89	0,03
C8b	0,93	0,05	Mettl6	0,47	0,00	Bivm	0,41	0,04	Samd7	-1,06	0,05
Fam151a	-1,72	0,02	Ebpl	0,54	0,00	Stat1	0,66	0,05	Skil	2,27	0,02
Calr4	-0,65	0,03	Mapk8	0,17	0,03	1700019D03Rik	1,27	0,00	Ndufb5	0,92	0,01
Urod	-0,98	0,03	Ints9	0,19	0,04	Ormdl1	0,67	0,00	Fxr1	-0,79	0,00
Kdm4a	-1,13	0,00	Esco2	0,13	0,04	Sf3b1	0,22	0,02	Ndufc1	2,18	0,01
Rragc	0,23	0,01	Ppp2r2a	-0,23	0,00	Mob4	0,92	0,00	Alg5	3,87	0,00
Mycbp	0,43	0,02	Rcbtb2	0,51	0,02	Coq10b	0,60	0,00	Siah2	0,33	0,04
Clspn	-0,36	0,03	Dad1	-0,23	0,04	9130024F11Rik	1,51	0,00	Ssr3	0,46	0,02
Psmb2	0,37	0,02	Haus4	0,34	0,00	Tyw5	0,93	0,00	Smc4	-0,26	0,03
Ak2	-0,57	0,02	Oxct1	0,16	0,04	1700066M21Rik	-0,81	0,00	Etfdh	2,62	0,04
Rsrp1	-0,59	0,03	Cmbl	0,76	0,02	Kctd18	-0,56	0,01	Rps3a1	1,41	0,05
Tmem201	0,35	0,04	Rrm2b	0,17	0,03	Sgol2	-0,27	0,02	Apoa1bp	2,94	0,01
Phf13	0,23	0,02	Dpys	-0,32	0,03	Bzw1	0,53	0,00	Arhgef2	3,31	0,03
Arhgef16	-1,08	0,03	Mief1	0,45	0,01	Ppil3	0,86	0,02	Cks1b	0,40	0,02
9330182L0	-0,66	0,03	Nudt16l1	0,54	0,02	Clk1	1,34	0,00	Atp8b2	3,41	0,05
Fbxl13	3,35	0,05	Cyc1	1,08	0,01	Orc2	-0,44	0,00	Slc39a1	3,24	0,00
Afap1	-0,72	0,04	Rabl2	0,29	0,01	Casp8	1,23	0,00	S100a1	3,34	0,01
Stk32b	-0,68	0,03	Tfg	0,29	0,01	Fzd7	-1,43	0,00	S100a10	2,67	0,03
Fip1l1	-0,64	0,01	Sdf2l1	0,36	0,01	Sumo1	0,68	0,00	Tdrkh	-0,54	0,05
Kit	0,45	0,01	Dnm1l	0,22	0,01	Fam117b	-0,62	0,00	Ecm1	3,22	0,01
Pptc7	0,43	0,01	Tnk2	-0,28	0,01	Cyp20a1	1,20	0,00	Mrps21	0,73	0,04
Ttyh3	0,65	0,04	Ndufb4	-0,60	0,04	Ino80d	-0,33	0,03	Hsd3b1	2,93	0,03
Rac1	0,51	0,00	Ets2	-0,23	0,01	Eef1b2	-0,35	0,01	Sike1	1,92	0,04
Smurf1	0,72	0,03	Scn8a	0,31	0,03	Ccnyl1	-0,67	0,00	Hipk1	1,85	0,02
Cdk8	0,50	0,02	Rpp14	0,24	0,02	2810408I11Rik	0,71	0,00	Ahcyl1	0,64	0,03
B3glct	-1,82	0,01	Creld2	0,31	0,00	D630023F18Rik	2,21	0,00	Tmem167b	1,49	0,04
Slc26a5	-0,94	0,02	Cdca3	-0,15	0,03	Idh1	0,60	0,03	Aknad1	-0,74	0,00
Tomm7	1,18	0,05	C1qtnf12	0,27	0,00	Kansl1l	-1,91	0,00	Fam102b	2,88	0,02
Rnf32	-1,05	0,00	Rhebl1	-0,18	0,04	Cps1	1,13	0,00	Slc35a3	1,15	0,04
Slc30a3	-0,66	0,00	Pigx	0,73	0,00	Pecr	0,88	0,00	Ostc	0,76	0,01
Slc4a1ap	-0,48	0,04	Hcfc1r1	-0,26	0,04	Tmem169	-0,66	0,02	Ints12	-0,47	0,00
Ppp1cb	0,72	0,00	Cyp39a1	0,16	0,02	Tmbim1	-0,43	0,04	Ube2d3	0,26	0,02
Tacc3	-0,89	0,00	Ttc27	0,16	0,04	Ctdsp1	-1,40	0,00	H2afz	2,28	0,01
Nop14	-0,54	0,03	Hagh	0,78	0,03	Stk36	0,70	0,00	Rpf1	0,33	0,04
Lyar	-0,31	0,03	Dusp1	-0,17	0,03	Abcb6	-1,13	0,03	Cth	0,67	0,01
Ncapg	-0,45	0,03	Cdh2	0,22	0,05	Cnppd1	-0,62	0,00	Cdk5rap2	-1,01	0,00
Ociad1	-0,98	0,05	Bag6	-0,38	0,05	Mff	0,81	0,00	Inpp5b	-0,73	0,01
Nup54	-0,29	0,05	Wdr33	-0,12	0,03	Slc19a3	1,40	0,02	Wasf2	-0,39	0,03
Tmem150	2,20	0,05	Trim26	0,27	0,03	Fbxo36	1,14	0,00	Nbn	-0,74	0,01
Ephx4	1,35	0,04	Ik	0,29	0,01	Sp100	1,57	0,00	Mms22l	0,40	0,05
Pgam5	-0,88	0,02	Pggt1b	0,23	0,03	Sp140	1,28	0,00	Melk	0,31	0,02
Pole	-1,92	0,04	Lars	0,25	0,02	Cab39	-0,55	0,00	AI314180	-0,52	0,00
Coq5	-1,28	0,01	Ptpn2	0,19	0,00	B3gnt7	-0,99	0,00	Ccdc171	-0,50	0,01
Sirt4	-0,62	0,01	Me2	0,33	0,02	Pde6d	1,43	0,00	Mllt3	-0,46	0,00
Nos1	-2,45	0,00	Tmx3	-0,27	0,05	Atg16l1	-0,72	0,00	Nfia	2,88	0,00
Oas1c	-0,37	0,04	Asrgl1	0,74	0,05	Dgkd	-0,57	0,01	Rab3b	-0,78	0,04
Mapkapk5	0,75	0,01	Zp1	0,19	0,02	Ackr3	0,62	0,05	Pik3r3	-0,43	0,01
Brap	0,25	0,02	Ddb1	-0,12	0,03	Cops8	-0,38	0,02	Eri3	4,11	0,00
Sh2b3	0,76	0,05	Rtn3	0,21	0,03	Rbm44	-0,77	0,04	Fbxo42	0,39	0,05
Atp2a2	0,29	0,03	Mrpl21	-0,20	0,03	Hes6	-0,69	0,05	Tmem51	3,19	0,03
Zfp655	-0,25	0,01	Cdk2ap2	-0,14	0,04	Ndufa10	0,42	0,01	Ssu72	0,35	0,04
Ptpro	0,47	0,04	Klc2	0,63	0,01	Capn10	-1,37	0,00	Rps20	2,16	0,04
Asz1	-0,30	0,05	Fam45a	0,33	0,00	02-Sep	0,42	0,01	Ufl1	-0,37	0,01
Rmnd5a	0,54	0,00	Aldh18a1	0,20	0,03	Dtymk	0,65	0,00	1700003M02Rik	-2,94	0,00
Nup210	1,87	0,04	Lcor	0,31	0,01	Zcchc2	-1,04	0,00	B4galt1	2,62	0,00
Cecr2	0,49	0,01	Nt5c2	0,27	0,03	Kdsr	-0,32	0,03	Rnf38	0,26	0,05
Atf7ip	-0,69	0,03	Tdrd1	0,26	0,02	Serpinb3a	0,81	0,00	Plin2	2,21	0,01
Ndufa4	0,40	0,03	Afap1l2	-0,12	0,02	Tsn	0,46	0,00	Fam151a	-1,70	0,01
Vwde	-1,68	0,00	Ints4	0,26	0,04	3110009E18Rik	0,42	0,04	Magoh	0,31	0,04
Pot1a	-0,63	0,04	Aspscr1	0,36	0,04	Actr3	-0,29	0,03	Echdc2	3,86	0,00
Rbm28	-0,41	0,05	Mob2	0,34	0,01	Ubxn4	-0,45	0,00	Calr4	-0,60	0,02
Slc35b4	-0,70	0,01	Ogfod3	-0,51	0,03	Cd55	0,46	0,00	Stil	-1,88	0,04
Parp12	0,26	0,01	Fyco1	0,15	0,03	Il20	1,50	0,00	Uqcrh	0,62	0,02
Nobox	-0,74	0,00	Rps26	-0,52	0,02	Fam72a	0,31	0,02	Nasp	-0,52	0,01
Repin1	0,61	0,03	Rnf41	0,57	0,04	Slc41a1	-1,22	0,00	Atp6v0b	0,58	0,04
Fam221a	-0,57	0,01	Pias2	0,21	0,02	Slc45a3	-0,98	0,00	Cited4	3,91	0,01
Tax1bp1	-0,30	0,01	Paox	0,37	0,03	Dstyk	-0,71	0,00	D830031N03Rik	-1,43	0,04
Tril	1,46	0,02	Psmd13	1,01	0,02	Tmem81	-2,12	0,00	Clspn	-0,27	0,05
Elmod3	-2,57	0,04	Apool	0,27	0,02	Mdm4	0,93	0,00	E330017L17Rik	3,21	0,05
Hk2	0,87	0,04	Sumf2	0,68	0,05	Atp2b4	-0,88	0,02	Xkr8	3,01	0,01
Pcbp1	0,40	0,02	Mkln1	-0,13	0,03	Btg2	0,54	0,00	Sh3bgrl3	1,03	0,03
Mcm2	0,36	0,03	Usp16	0,18	0,01	Ube2t	1,44	0,00	Pnrc2	1,00	0,04
Wnt7a	1,54	0,04	Mcrip2	-0,59	0,02	Kif14	-0,36	0,00	Hmgcl	3,43	0,01
Thumpd3	-1,47	0,03	Plk4	0,13	0,03	Zfp281	0,71	0,05	Tcea3	-0,64	0,00
Mbd4	-0,71	0,05	Mfsd8	0,64	0,01	F13b	1,86	0,03	Camk2n1	3,08	0,01
Tmem72	-1,01	0,01	Nrp1	0,17	0,03	Rgs2	0,58	0,00	Emc1	3,16	0,00
Bid	2,27	0,03	Thoc3	0,35	0,01	BC003331	0,63	0,00	Sdhb	2,69	0,00
Pex26	0,78	0,02	Tfap2b	0,29	0,04	Prg4	0,69	0,00	Zbtb17	2,81	0,02
Usp5	-1,88	0,01	Tmem14a	-0,24	0,03	Rnf2	-0,23	0,03	Fbxo6	3,26	0,05
Tulp3	0,77	0,00	Tmem70	0,26	0,02	Shcbp1l	0,52	0,02	Acot7	1,64	0,05
Arntl2	-0,43	0,05	Fastkd2	0,26	0,03	Rgsl1	1,01	0,01	Mmp23	3,72	0,04
Ipo8	0,29	0,02	Ndufb3	-0,42	0,00	Tor1aip1	-0,40	0,01	Slc35e2	3,31	0,02
Adamts17	2,08	0,00	Il1r2	0,50	0,04	Tnn	-1,41	0,02	Coq2	0,69	0,05
Ticrr	0,30	0,05	Ormdl1	0,25	0,01	Mrps14	0,52	0,00	Pclo	-0,62	0,04
Nlrp4e	0,55	0,03	Mff	-0,17	0,05	Serpinc1	0,41	0,01	9330182L06Rik	-0,67	0,03
Rhpn2	0,33	0,01	Fam135a	0,26	0,03	Myoc	0,72	0,04	Akap9	-0,38	0,02
Tarsl2	2,55	0,01	Sdhaf4	-0,35	0,00	7420461P10Rik	0,87	0,00	Fam126a	0,59	0,03
Rab38	-0,68	0,01	Serpinb8	0,74	0,02	Prrc2c	-0,27	0,01	Cenpa	0,42	0,03
Xrra1	-1,84	0,04	Nr5a2	0,35	0,00	Scyl3	-0,69	0,03	Stk32b	-0,79	0,02
Sox6	2,29	0,05	Ube2t	-0,25	0,04	F5	2,24	0,01	Stim2	-0,61	0,00
Cdr2	0,57	0,00	Slc45a3	-0,16	0,04	Blzf1	0,68	0,00	Pds5a	-1,25	0,04
Prkcb	2,14	0,01	Ahctf1	-0,14	0,03	Sft2d2	-1,22	0,00	Rfc1	-0,40	0,01
Tial1	0,65	0,02	Mpzl1	0,37	0,02	Creg1	-1,79	0,00	Fip1l1	-0,49	0,02
Tacc2	0,28	0,05	Tex35	-1,00	0,04	Aldh9a1	-0,42	0,02	Pkd2	1,06	0,05
Obox5	0,24	0,03	Ralgps2	0,13	0,03	Tmco1	0,50	0,00	Clip1	-0,44	0,02
Strn4	3,11	0,02	Iars2	0,66	0,02	Nuf2	0,87	0,00	Mphosph9	-0,71	0,00
Zfp296	-0,77	0,01	Nek2	0,46	0,00	Uhmk1	-1,10	0,00	Prkrip1	-0,69	0,02
Nlrp5	-0,39	0,03	Fam107b	-0,15	0,01	Dusp12	0,31	0,01	Rac1	0,43	0,01
Zfp235	0,66	0,01	Fmo4	0,48	0,00	Sdhc	0,48	0,00	Rnf216	-0,56	0,02
Zfp108	-0,96	0,00	Stxbp1	0,43	0,02	Usp21	-0,70	0,01	Cdk8	0,48	0,03
Ceacam2	-1,44	0,01	Tsc1	0,29	0,03	Alyref2	-1,73	0,00	Fzd1	2,60	0,03

Table S7: GO annotation revealed common biological processes involved in PNA, Peripubertal androgenized, 17NF, and maternal obesity animal models

PNA				Peripubertal androgenized				17NF				Maternal obesity			
ID	Term	p-value	adjust.p	ID	Term	p-value	adjust.p	ID	Term	p-value	adjust.p	ID	Term	p-value	adjust.p
GO:0060070	Canonical Wnt signaling pathway	0,00	0,00	GO:0006633	Fatty acid biosynthetic process	0,01	0,01	GO:0090090	Canonical Wnt signaling pathway	0,00	0,00	GO:0046034	ATP metabolic process	4,90E-08	4,90E-08
GO:0032869	Response to insulin	0,04	0,04	GO:0042593	Glucose metabolic process	0,00	0,00	GO:0001541	Ovarian follicle development	0,05	0,05	GO:0001824	Blastocyst development	0,00	0,00
GO:0006338	Chromatin remodeling	0,01	0,01	GO:0008286	Response to insulin	0,04	0,04	GO:0032868	Response to insulin	0,00	0,00	GO:0006338	Chromatin remodeling	0,02	0,02
GO:0045137	Development of primary sexual characteristics	0,00	0,00	GO:1902275	Chromatin remodeling	0,03	0,03	GO:0045137	Development of primary sexual characteristics	0,01	0,01	GO:0006306	DNA methylation	0,04	0,04
GO:0007281	Germ cell development	0,00	0,00	GO:0044784	Meiotic cell cycle	0,02	0,02	GO:0006338	Chromatin remodeling	0,00	0,00	GO:0009062	Fatty acid biosynthetic process	0,02	0,02
GO:0006006	Glucose metabolic process	0,01	0,01	GO:0046034	ATP metabolic process	0,01	0,01	GO:0070192	Meiotic cell cycle	0,02	0,02	GO:0061647	Histone modification	0,03	0,03
GO:0051321	Meiotic cell cycle	0,00	0,00	GO:0001835	Blastocyst development	0,02	0,02	GO:0007281	Germ cell development	0,02	0,02	GO:0051321	Meiotic cell cycle	0,00	0,00
GO:0006306	DNA methylation	0,01	0,01	GO:0006338	Histone modification	0,03	0,03	GO:0006006	Glucose metabolic process	0,00	0,00	GO:0032868	Response to insulin	0,04	0,04
GO:0006006	Glucose metabolic process	0,01	0,01	GO:0006479	DNA methylation	0,02	0,02	GO:0016570	Histone modification	0,00	0,00	GO:0006007	Glucose metabolic process	0,01	0,01
GO:0032872	Regulation of stress-activated MAPK cascade	0,01	0,01					GO:0006306	DNA methylation	0,00	0,00	GO:0045137	Development of primary sexual characteristics	0,01	0,01
GO:0036124	Histone modification	0,01	0,01					GO:0001824	Blastocyst development	0,00	0,00				
								GO:0043401	Steroid hormone signaling pathway	0,00	0,00				
								GO:0032873	Regulation of stress-activated MAPK cascade	0,05	0,05				
								GO:0038127	ERBB signaling pathway	0,01	0,01				

Table S8: Transcriptomic analysis of subcutaneous adipose tissue showed common and unique DEGs in PNA, Peripubertal androgenized, 17NF, and maternal obesity animal models

PNA			Peripubertal androgenized			17NF			Maternal Obesity		
Gene	Log2FoldChange	P-value	Gene	Log2FoldChange	P-value	Gene	Log2FoldChange	P-value	Gene	Log2FoldChange	P-value
Jph2	-6,26	0,00	*Alkal2*	3,63	0,00	*Lactb2*	-0,89	0,00	*2610203C22Rik*	-1,71	0,03
Tpm1	-4,66	0,00	*C4a*	1,66	0,00	*Tfap2b*	1,36	0,04	*Cops5*	-0,68	0,04
Myoz1	-9,00	0,00	*Abhd2*	-1,30	0,00	*Mcm3*	1,43	0,01	*Lactb2*	-0,78	0,03
Ampd1	-7,45	0,00	*Agt*	2,38	0,00	*Col9a1*	-3,06	0,02	*Rpl5-ps1*	1,35	0,02
Klhl41	-7,12	0,00	*Acss2*	-1,83	0,00	*Ptp4a1*	-0,90	0,02	*Ptpn18*	1,16	0,04
Ddit4l	-5,93	0,00	*Areg*	-4,14	0,00	*Ptpn18*	1,22	0,01	*Neurl3*	1,66	0,02
Pvalb	-8,28	0,00	*Rprml*	2,90	0,00	*Zap70*	2,02	0,03	*Arid5a*	1,29	0,03
Tnnc2	-8,05	0,00	*Slc7a2*	-2,71	0,00	*Il18r1*	1,94	0,00	*Ankrd23*	-3,86	0,01
Tmem38a	-5,58	0,00	*Ankrd2*	2,53	0,00	*Slc40a1*	-1,46	0,02	*Zap70*	4,11	0,00
Ttn	-6,08	0,00	*Prelp*	1,86	0,00	*Stat1*	0,78	0,03	*Coa5*	-0,55	0,04
Myom1	-5,80	0,00	*Rps2*	-0,55	0,00	*Ankrd4*	0,80	0,04	*Aff3*	1,66	0,04
Hspb6	-4,43	0,00	*Emp1*	2,04	0,00	*Coq10b*	-1,22	0,00	*Creg2*	3,93	0,05
Pgam2	-8,22	0,00	*Itgbl1*	2,28	0,00	*Cd28*	1,60	0,04	*Il18r1*	2,81	0,00
Mybph	-6,54	0,00	*Procr*	1,62	0,00	*Icos*	1,94	0,01	*Il18rap*	2,40	0,02
Pfkm	-4,28	0,00	*Sorcs2*	-2,34	0,00	*Ndufs1*	-0,61	0,01	*Mettl21c*	-2,97	0,00
Sh3bgr	-5,77	0,00	*Ifi27l2a*	1,53	0,00	*Fn1*	0,83	0,03	*Stat4*	2,46	0,03
Dhrs7c	-6,90	0,00	*Ccl11*	1,56	0,00	*Pecr*	-1,45	0,01	*Nemp2*	2,08	0,03
Ppp1r3a	-7,58	0,00	*Scara5*	1,49	0,00	*Pinc*	-4,04	0,03	*Stk17b*	1,98	0,00
Smtnl2	-5,68	0,00	*Fam213*	1,63	0,00	*C53004*	-4,53	0,03	*Ankrd44*	1,22	0,02
Mup11	-2,59	0,00	*Asph*	1,34	0,00	*Rufy4*	-2,91	0,00	*Hspe1*	-0,72	0,00
Tmod4	-6,73	0,00	*Slco2b1*	1,64	0,00	*Arpc2*	0,53	0,03	*Clk1*	0,57	0,02
Ckm	-8,74	0,00	*Rarres2*	1,91	0,00	*Mogat1*	-0,87	0,03	*Fzd7*	-0,94	0,04
Hfe2	-6,83	0,00	*Tead2*	-2,08	0,00	*Dock10*	1,28	0,02	*Cd28*	3,11	0,00
Myh1	-7,19	0,00	*Cd209d*	1,46	0,00	*Irs1*	-0,81	0,01	*Rpl18-ps1*	1,12	0,03
Ak1	-4,59	0,00	*Cfb*	1,86	0,00	*A53003*	1,83	0,05	*Icos*	3,72	0,00
Casq1	-10,73	0,00	*Ecm2*	1,36	0,00	*A53004*	1,82	0,02	*Rpl10a-ps1*	1,19	0,00
Ryr1	-5,80	0,00	*Lgals7*	-3,64	0,00	*Sp110*	1,63	0,00	*Pecr*	-0,98	0,02
Pygm	-7,73	0,00	*Acaca*	-1,41	0,00	*Sp140*	1,29	0,01	*Plcd4*	-2,69	0,01
Nexn	-4,28	0,00	*Endou*	2,46	0,00	*Sp100*	0,97	0,03	*Cyp27a1*	-0,76	0,03
Tcap	-8,39	0,00	*Krtdap*	4,16	0,00	*311007*	3,15	0,00	*Prkag3*	-3,34	0,01
Synm	-3,71	0,00	*Paip1*	-1,06	0,00	*Inpp5d*	1,18	0,00	*Tuba4a*	-1,43	0,01
Mybpc2	-7,79	0,00	*Alas2*	2,06	0,00	*Ugt1a6*	-3,24	0,01	*Des*	-3,04	0,03
Itgb1bp2	-5,92	0,00	*Tnxb*	1,75	0,00	*Ugt1a6*	-2,38	0,00	*Speg*	-2,90	0,00
Speg	-3,56	0,00	*Slc6a13*	2,46	0,00	*Arl4c*	1,25	0,04	*Dock10*	1,96	0,00
Apobec2	-6,56	0,00	*Slc44a4*	-3,41	0,00	*Dsel*	-0,97	0,04	*A630081D01Rik*	2,90	0,03
Rtn2	-4,76	0,00	*Anxa6*	0,83	0,00	*Steap3*	-1,06	0,01	*Irs1*	-0,89	0,03
Mybpc1	-6,23	0,00	*Sulf2*	1,27	0,00	*Mcm6*	1,48	0,00	*Snord82*	1,26	0,04
Prr33	-6,50	0,00	*Mustn1*	1,50	0,00	*Chil1*	-1,94	0,01	*Inpp5d*	2,05	0,00
Srl	-5,47	0,00	*Sorl1*	-1,86	0,00	*Ppfia4*	1,45	0,04	*Arl4c*	1,77	0,01
Smyd1	-5,13	0,00	*Lamb2*	1,32	0,00	*Ptpn7*	1,40	0,01	*Per2*	2,03	0,01
Flnc	-3,86	0,00	*Lox*	1,59	0,00	*Phlda3*	-0,96	0,00	*St8sia4*	1,40	0,00
Sgca	-6,54	0,00	*Fhl1*	1,99	0,00	*Aspm*	2,99	0,02	*Bcl2*	1,72	0,00
Sypl2	-6,86	0,00	*Agps*	-1,41	0,00	*B3galt2*	-0,67	0,04	*Steap3*	-0,86	0,02
Trdn	-7,10	0,00	*N0t*	2,70	0,00	*Rgs1*	2,23	0,02	*Marco*	4,02	0,04
Pdlim3	-3,62	0,00	*Hnrnpa*	-0,82	0,00	*Ncf2*	1,08	0,02	*Cxcr4*	1,73	0,04
Mypn	-7,27	0,00	*Ifngr1*	0,99	0,00	*Acbd6*	-0,64	0,04	*Rassf5*	2,37	0,00
Mrps24	-1,01	0,00	*Prodh*	1,43	0,00	*Qsox1*	-0,86	0,02	*Lax1*	2,62	0,02
Alpk3	-7,25	0,00	*Ramp2*	1,16	0,00	*Ralgps2*	1,86	0,01	*Prelp*	0,73	0,01
Adssl1	-3,71	0,00	*Thbd*	1,71	0,00	*493052*	0,68	0,02	*Btg2*	0,92	0,03
Dusp13	-7,00	0,00	*261052*	-5,29	0,00	*Fmo1*	-1,01	0,01	*Ppp1r12b*	-0,96	0,05
Mylk4	-5,88	0,00	*Dkkl1*	-2,80	0,00	*Fmo2*	-1,09	0,01	*Ptpn7*	2,09	0,01
Pkia	-3,39	0,00	*Slc43a3*	1,50	0,00	*Atp1b1*	-1,10	0,01	*Shisa4*	-2,32	0,00
Des	-3,80	0,00	*Pon1*	2,57	0,00	*Xcl1*	2,47	0,01	*Kif21b*	2,90	0,00
Pdlim7	-2,67	0,00	*Mmp9*	1,74	0,00	*Dcaf6*	-1,10	0,00	*Ptprc*	2,53	0,01
Ttll7	-3,66	0,00	*Prom1*	-3,12	0,00	*Mpc2*	-0,63	0,05	*A130071D04Rik*	2,85	0,00
Hrc	-7,80	0,00	*Dmkn*	2,83	0,00	*Adcy10*	-1,31	0,03	*Cac01e*	3,42	0,01
Kcnj11	-5,31	0,00	*Cldn6*	-5,20	0,00	*Mpzl1*	-1,36	0,02	*Ralgps2*	2,29	0,00
Stac3	-5,33	0,00	*Bgn*	0,76	0,00	*Cd247*	1,78	0,04	*4930523C07Rik*	1,33	0,00
Jph1	-4,46	0,00	*C2*	1,04	0,00	*Tmco1*	-0,66	0,02	*Sell*	4,10	0,01
Cacnb1	-4,04	0,00	*Tmem2*	-1,50	0,00	*Hsd17b*	-1,12	0,04	*Cd247*	2,39	0,02
Tmem52	-5,42	0,00	*Rassf3*	-1,26	0,00	*Fcgr2b*	0,84	0,03	*Dusp27*	-3,74	0,02
Phka1	-3,18	0,00	*Acvrl1*	0,86	0,00	*Fcgr4*	1,75	0,04	*Pbx1*	-0,55	0,04
Tmod1	-3,58	0,00	*Col4a3*	5,75	0,00	*Fcer1g*	1,16	0,00	*Fcrla*	3,95	0,03
Jsrp1	-8,34	0,00	*Rgs7bp*	2,00	0,00	*Arhgap*	1,33	0,03	*Pcp4l1*	-1,64	0,05
Ly6d	3,24	0,00	*Oxtr*	-2,87	0,00	*Ly9*	1,72	0,03	*Ufc1*	-0,77	0,02
Rbfox1	-5,25	0,00	*Phgdh*	1,37	0,00	*Slamf7*	2,12	0,00	*Rpl27-ps1*	1,11	0,04
Mup10	-2,11	0,00	*Serpine*	1,80	0,00	*Cd48*	1,48	0,01	*Arhgap30*	2,29	0,00
Ank1	-7,63	0,00	*B93009*	5,48	0,00	*Tma7-p*	1,14	0,00	*Ly9*	2,56	0,00
Wfdc1	-5,04	0,00	*Nnmt*	1,75	0,00	*Pex19*	-0,80	0,01	*Slamf7*	1,72	0,03
Plpp7	-3,78	0,00	*Tshz2*	1,44	0,00	*Tagln2*	0,68	0,02	*Cd48*	1,31	0,05
Cacng1	-8,17	0,00	*Chrdl1*	1,28	0,00	*Aim2*	1,78	0,02	*Cd84*	1,59	0,01
Pkib	3,52	0,00	*Slc35f3*	-4,55	0,00	*Ifi206*	3,32	0,00	*Aim2*	1,69	0,02
Lrrc2	-5,60	0,00	*Lbp*	1,85	0,00	*Ifi213*	2,75	0,00	*Ifi214*	2,42	0,02
Mylf-ps	-5,39	0,00	*Irx3*	-2,43	0,00	*Ifi209*	2,46	0,04	*Ifi209*	1,73	0,01
Tcea3	-3,44	0,00	*Tgfbr3*	0,86	0,00	*Ifi203-p*	-1,57	0,01	*Kmo*	2,17	0,02
Ppp1r14c	-5,72	0,00	*C1ra*	0,95	0,00	*Mndal*	1,45	0,00	*Cnst*	-0,92	0,05
Synpo2l	-4,98	0,00	*Postn*	-1,57	0,00	*Ifi203*	1,06	0,01	*Coq8a*	-0,96	0,05
Neurl1a	-5,35	0,00	*Trp53in*	1,60	0,00	*Ifi205*	0,84	0,02	*Ephx1*	0,71	0,02
Tuba8	-3,47	0,00	*C1s1*	1,12	0,00	*Kmo*	1,72	0,01	*Dusp10*	-1,39	0,01
Lrrc14b	-4,54	0,00	*Chdh*	1,21	0,00	*Cnst*	-1,21	0,00	*Prox1*	2,40	0,02
Hspb2	-2,84	0,00	*Anxa8*	2,04	0,00	*Sccpdh*	-1,34	0,00	*Traf3ip3*	2,77	0,01
Rpgrip1	2,70	0,00	*Cks2*	-2,14	0,00	*Coq8a*	-0,71	0,02	*Cr2*	4,42	0,02
Mylk2	-8,94	0,00	*Serpi03*	1,63	0,00	*Hhipl2*	-2,47	0,02	*Fam107b*	1,23	0,02
Atcayos	-6,11	0,00	*Serpi03*	2,51	0,00	*Rpl21-p*	-2,12	0,04	*Atp5c1*	-0,76	0,02
Klhl31	-8,77	0,00	*Prrg3*	2,20	0,00	#####	-0,96	0,01	*Hacd1*	-1,80	0,01
Trim54	-7,25	0,00	*Cd9*	-1,33	0,00	#####	-0,64	0,03	*Pip4k2a*	1,20	0,03
Prkab2	-2,22	0,00	*Pth1r*	-1,38	0,00	*Lyplal1*	-1,77	0,00	*Apbb1ip*	1,56	0,00
Cox6a2	-7,42	0,00	*Il1r2*	2,32	0,00	*Kcnk2*	-1,47	0,00	*Psd4*	2,61	0,00
Shisa4	-2,81	0,00	*Cd163*	1,64	0,00	*Cenpf*	1,97	0,04	*Arrdc1*	1,21	0,03
Shisa2	-3,52	0,00	*Vim*	0,89	0,00	*Hsd11b*	-0,76	0,00	*Fut7*	3,29	0,01
Cac01e	4,51	0,00	*Mest*	2,47	0,00	*Echdc3*	-0,90	0,02	*Rpsa-ps9*	0,79	0,04
Ndufaf5	-2,55	0,00	*Slc15a5*	-5,43	0,00	*Etl4*	-0,80	0,04	*Ptges*	-1,11	0,02
Mrln	-5,99	0,00	*Cd24a*	-2,09	0,00	*Hnmt*	-1,01	0,02	*Fnbp1*	0,86	0,01
Myhas	-6,46	0,00	*Cers4*	1,17	0,00	*Psd4*	1,95	0,01	*Qrfp*	4,95	0,02
Coro2a	1,06	0,00	*Mt2*	1,21	0,00	*Agpat2*	-0,89	0,00	*Plpp7*	-2,92	0,01
Stap1	3,70	0,00	*Haus3*	-1,37	0,00	*Slc2a6*	2,25	0,05	*1110008P14Rik*	-0,75	0,03
Tpbgl	-3,11	0,00	*Lrp1*	1,17	0,00	*Vav2*	0,99	0,02	*Ak1*	-3,27	0,02
Cxcr5	5,40	0,00	*Rab3ip*	-1,13	0,00	*Cel*	-3,42	0,03	*Sh2d3c*	1,37	0,02
Nedd9	1,89	0,00	*Pde11a*	2,49	0,00	*Crat*	-0,96	0,00	*Traf1*	2,57	0,00
Abra	-5,56	0,00	*Slc2a13*	1,76	0,00	*Ptges*	-1,03	0,00	*Psmb7*	-0,72	0,03
Asb12	-6,08	0,00	*Mmd2*	-3,88	0,00	*Hmcn2*	-1,26	0,04	*Arhgap15*	2,51	0,00
Siglecg	4,47	0,00	*Mpzl2*	-1,74	0,00	*Ass1*	1,83	0,00	*Cytip*	1,57	0,00
Smarcd3	-2,54	0,00	*Dctn3*	0,89	0,00	*Uck1*	-1,02	0,00	*Ccdc148*	4,59	0,04
Slc15a5	-2,11	0,00	*Smim1*	1,51	0,00	*Traf1*	1,39	0,04	*Ldha-ps*	-1,52	0,05
Myot	-7,40	0,00	*Fgfr2*	-2,02	0,00	*Rpl35*	0,50	0,01	*Ly75*	2,50	0,01
Scn1b	-1,99	0,00	*BC0308*	-5,41	0,00	*Kif5c*	1,35	0,05	*Slc25a12*	-1,58	0,02
Trim63	-9,15	0,00	*Map2*	-2,88	0,00	*Nmi*	0,78	0,04	*Map3k20*	-1,08	0,02
Capn3	-3,95	0,00	*Aebp1*	1,14	0,00	*Pla2r1*	-1,14	0,04	*Wipf1*	1,19	0,02
Asb10	-5,27	0,00	*Etv6*	-1,06	0,00	*Scn7a*	-0,88	0,00	*Osbpl6*	-1,89	0,03
Asb16	-5,98	0,00	*Cldn8*	-3,22	0,00	*Itga6*	-0,53	0,04	*Itga4*	1,59	0,02
AI662270	3,18	0,00	*Tnxa*	3,54	0,00	*Pdk1*	-0,93	0,01	*Madd*	1,42	0,00
Nos1	-3,68	0,00	*Pdk1*	-1,02	0,00	*Chn1*	-1,29	0,00	*Pacsin3*	-1,49	0,01
Mb	-4,04	0,00	*Adam1*	-0,74	0,00	*Hoxd8*	-1,05	0,01	*Mapk8ip1*	-1,07	0,04
Mettl21c	-3,28	0,00	*Nme1*	-0,87	0,00	*Itga4*	1,25	0,03	*Slc35c1*	1,29	0,02
Plcd4	-3,89	0,00	*Egr2*	-3,39	0,00	*Nckap1*	-0,65	0,01	*B230118H07Rik*	-0,94	0,03
Rragd	-4,26	0,00	*Serpine*	2,60	0,00	*Olfr103*	-1,74	0,05	*Cd44*	1,19	0,02
Sit1	6,47	0,00	*Pygl*	-1,30	0,00	*Mtch2*	-0,72	0,03	*Immp1l*	-0,99	0,02
Mup17	-2,13	0,00	*Entpd2*	1,34	0,00	*Spi1*	1,31	0,03	*BB218582*	-4,26	0,03
Tmem233	-7,81	0,00	*Perp*	-2,53	0,00	*Pex16*	-0,69	0,03	*Rasgrp1*	3,05	0,00
Ckmt2	-8,66	0,00	*Trim29*	-2,83	0,00	*Mapk8i*	-1,07	0,01	*Plcb2*	2,46	0,01
Coch	6,09	0,00	*Sfrp5*	2,64	0,00	*Trp53i1*	1,01	0,01	*Exd1*	-1,63	0,01
Fbxo40	-5,03	0,00	*Csf1r*	1,03	0,00	*Tspan1*	-0,84	0,00	*Mga*	0,54	0,04
Icos	4,01	0,00	*Heph*	1,49	0,00	*Hsd17b*	-1,02	0,00	*Capn3*	-2,90	0,01
Kif21b	3,16	0,00	*Palm*	1,69	0,00	*Ldlrad3*	-1,79	0,00	*Zfp106*	-1,10	0,03
Arhgap30	2,84	0,00	*Casp12*	1,09	0,00	*Pdhx*	-0,89	0,00	*Ppip5k1*	-0,90	0,02
1110008P14R	-0,84	0,00	*Tmem5*	-2,84	0,00	*Rasgrp1*	1,77	0,02	*Hypk*	0,99	0,04
Arhgap45	3,14	0,00	*Ednrb*	1,11	0,00	*Plcb2*	1,48	0,03	*Dusp2*	3,40	0,01
Myadml2	-6,26	0,00	*Il34*	1,45	0,00	*Rmdn3*	-0,98	0,00	*Bcl2l11*	1,00	0,01
Mup16	-2,21	0,00	*S100a1*	-3,72	0,00	*Chac1*	-1,96	0,04	*Cdc25b*	1,16	0,04
Ly9	2,81	0,00	*Prr32*	3,18	0,00	*Ehd4*	0,62	0,04	*Rassf2*	0,91	0,01
Clcn1	-6,24	0,00	*C5ar1*	1,51	0,00	*Slc28a2*	0,97	0,04	*Ndufaf5*	-1,67	0,02
Mup15	-2,33	0,00	*Cygb*	0,95	0,00	*Slc30a4*	-0,92	0,02	*Insm1*	4,91	0,02
Zc3h12d	4,15	0,00	*Cutal*	-2,91	0,00	*Fbn1*	0,83	0,05	*Tcf15*	-1,17	0,04
Btk	3,12	0,00	*Ccdc42*	2,87	0,00	*Secisbp*	-0,52	0,05	*Hck*	1,64	0,03
Chchd2-ps	-1,23	0,00	*Atp2b4*	1,53	0,00	*Prom2*	2,27	0,01	*Tspyl3*	2,10	0,02
Cd79b	4,41	0,00	*Raver2*	1,45	0,00	*Sirpa*	0,78	0,04	*Bpifb2*	3,69	0,03
Prkag3	-3,78	0,00	*Dpt*	1,60	0,00	*Atrn*	-1,22	0,01	*Bpifb3*	5,29	0,01
Fbp2	-3,98	0,00	*Tmem4*	1,58	0,00	*170003*	-0,91	0,00	*Acss2*	-0,92	0,04
Iglc2	4,32	0,00	*H6pd*	1,23	0,00	*Cdc25b*	1,10	0,02	*N0t*	1,41	0,00
D0se1l3	3,88	0,00	*B43011*	2,19	0,00	*Smox*	1,47	0,04	*Jph2*	-3,49	0,02
2310009B15R	-1,21	0,00	*Nr2f6*	-1,03	0,00	*Lrrn4*	-3,31	0,00	*Stk4*	1,15	0,02
Igkv6-29	6,24	0,00	*Snhg18*	1,34	0,00	*Plcb1*	-0,99	0,02	*Wfdc2*	0,95	0,01
Nrap	-6,63	0,00	*Casc4*	-1,85	0,00	*Ralgapa*	-0,66	0,03	*Wfdc3*	1,44	0,04
Mup3	-2,26	0,00	*Abi3bp*	1,39	0,00	*Cst7*	2,79	0,00	*Cd40*	1,92	0,01
Ikzf1	3,15	0,00	*Fam124*	1,63	0,00	*Apmap*	-1,02	0,01	*Ncoa3*	0,96	0,03
Rasd2	-3,89	0,00	*Cst3*	0,70	0,00	*Srxn1*	-0,72	0,04	*Prex1*	1,20	0,04
2210408F21R	-1,75	0,00	*Calca*	-5,14	0,00	*Rps15a-*	-3,55	0,00	*Rpl30-ps5*	1,60	0,01
Tm6sf1	1,17	0,00	*Sesn2*	1,27	0,00	*Hck*	1,53	0,03	*Prelid3b*	-0,66	0,03
Sell	4,07	0,00	*Ccnd1*	-1,47	0,00	*Acss2*	-1,16	0,00	*Zfp831*	3,73	0,01
Csf2rb2	2,37	0,00	*Cadm3*	1,84	0,00	*Acss2os*	-4,22	0,03	*Slc17a9*	2,40	0,00
Mup1	-2,18	0,00	*Klhl2*	1,35	0,00	*Samhd1*	1,18	0,00	*Helz2*	1,18	0,05
Bcl11a	3,86	0,00	*953001*	-3,33	0,00	*N0t*	3,78	0,00	*Rgs19*	0,94	0,05
Fbxo32	-1,83	0,00	*Aspn*	1,49	0,00	*Tgm2*	0,96	0,02	*Magix*	-1,99	0,02
Mir142	4,16	0,00	*E0h*	-1,05	0,00	*Snhg11*	-0,99	0,00	*Pim2*	2,36	0,01
Prkaa2	-2,60	0,00	*Slc25a2*	0,97	0,00	*Pkig*	0,77	0,02	*Was*	1,95	0,01
Fam169b	4,03	0,00	*Tmem1*	-1,66	0,00	*Wisp2*	-2,35	0,00	*Srpx*	0,66	0,04
Slc16a3	-4,18	0,00	*Dsg2*	-2,62	0,00	*Rims4*	-1,41	0,03	*Rpl30-ps11*	2,63	0,00
Samsn1	2,86	0,00	*Cfh*	1,01	0,00	*Wfdc2*	2,37	0,03	*Maob*	-1,11	0,01
Fam13b	1,59	0,00	*Stard10*	-1,71	0,00	*Ctsa*	0,57	0,02	*Dock11*	1,42	0,00
Rilpl1	-2,06	0,00	*H19*	-3,54	0,00	*Prex1*	1,28	0,02	06-Sep	1,79	0,00
Mettl22	-2,04	0,00	*Ms4a4d*	1,13	0,00	*Kcnb1*	-0,83	0,01	*Sash3*	3,19	0,00
Fam213b	-1,57	0,00	*Sbsn*	1,76	0,00	*Cebpb*	-1,13	0,00	*Elf4*	2,00	0,01
Elf4	2,45	0,00	*Tph1*	-3,40	0,00	*Ptpn1*	0,67	0,03	*Stk26*	2,42	0,01
Obscn	-7,76	0,00	*Cxcl14*	1,47	0,00	*Zbp1*	2,42	0,00	*Rps2-ps13*	0,73	0,05
Sar1b	-1,77	0,00	*Cnr1*	1,90	0,00	*Nkain4*	-5,93	0,01	*Fhl1*	-1,12	0,03
Tespa1	3,39	0,00	*Mdk*	-1,59	0,00	*Helz2*	1,24	0,01	*Xlr4c*	3,06	0,04
Myom2	-8,01	0,00	*Hspa12*	1,27	0,00	*Foxp3*	2,62	0,01	*Haus7*	-0,83	0,05
Ighv1-78	6,34	0,00	*Fgfr1*	1,08	0,00	*Pim2*	1,29	0,02	*Bgn*	0,64	0,05
H2-Ob	4,23	0,00	*Sgcd*	1,60	0,00	*Was*	1,74	0,01	*Arhgap4*	2,75	0,00
Dnlz	-0,88	0,00	*Acot1*	-3,02	0,00	*Cybb*	1,32	0,00	*F8*	2,96	0,00
Mup18	-2,80	0,00	*S100a1*	0,85	0,00	*Tspan7*	-0,83	0,01	*Fundc2*	-0,76	0,05
Camk2a	-3,80	0,00	*Ermp1*	0,91	0,00	*Rpl3-ps*	-0,70	0,01	*Rps24-ps3*	0,97	0,04
Lck	3,86	0,00	*Map3k6*	1,35	0,00	*Cfp*	0,79	0,01	*Apoo*	-1,12	0,04
H19	-3,16	0,00	*Hmgcl*	0,75	0,00	*Dock11*	1,05	0,01	*Asb12*	-3,29	0,02
Hrasls	-5,03	0,00	*Idh2*	0,80	0,00	*Pgrmc1*	-0,78	0,02	*Msn*	0,78	0,03
Ppm1l	-2,08	0,00	*Krt23*	-5,12	0,00	*Prr32*	4,55	0,01	*Foxo4*	-1,14	0,03
Flt3	3,63	0,00	*Cnn3*	-0,65	0,00	*Sash3*	1,45	0,04	*Il2rg*	2,64	0,00
Ctxn3	-5,87	0,00	*Dab2*	1,13	0,00	*Elf4*	1,32	0,02	*Itgb1bp2*	-3,90	0,04
Clec2i	3,01	0,00	*Cdk1*	-2,82	0,00	*Rps2-ps*	1,24	0,00	*Phka1*	-2,55	0,00
B430306N03	3,55	0,00	*Cyb5r4*	-0,74	0,00	*Phf6*	0,88	0,04	*Zdhhc15*	2,01	0,04
Car14	-4,21	0,00	*Set*	-0,73	0,00	*Mmgt1*	-1,11	0,00	*Pgk1*	-1,04	0,02
Cd53	2,30	0,00	*Upk2*	-4,08	0,00	*Slc9a6*	-1,39	0,01	*P2ry10*	3,65	0,00
Nctc1	-7,03	0,00	*C1qb*	0,98	0,00	*Nsdhl*	-1,16	0,00	*Btk*	2,70	0,00
Mustn1	-2,31	0,00	*Tob1*	-1,08	0,00	*Zfp185*	-1,77	0,02	*Bex4*	-2,66	0,02
Arhgap27os3	5,62	0,00	*Sema3e*	3,67	0,00	*Bgn*	0,61	0,03	*Tceal5*	-2,23	0,05
Cap2	-3,29	0,00	*Unc5a*	-1,45	0,00	*Srpk3*	2,88	0,01	*Bex1*	-1,47	0,04
H2-DMb2	3,93	0,00	*Mfsd2a*	-3,57	0,00	*Arhgap*	1,46	0,04	*Zcchc18*	2,94	0,04
Cystm1	-2,17	0,00	*Basp1*	-1,12	0,00	*Maged1*	-0,72	0,01	*Tsc22d3*	0,83	0,03
Lbx1	-5,81	0,00	*Rtl8c*	1,16	0,00	*Srpx2*	1,15	0,01	*Sh3kbp1*	0,83	0,01
Igkv4-59	5,54	0,00	*Neat1*	0,89	0,00	*Btk*	1,37	0,03	*Nhs*	-2,11	0,05
Sox6os	-2,07	0,00	*Wfdc21*	2,06	0,00	*Chrdl1*	-1,24	0,05	*Rps24-ps2*	1,32	0,02
Ighv1-58	6,03	0,00	*Ebf1*	0,61	0,00	*Pfkfb1*	-0,86	0,03	*Pkia*	-2,97	0,00
Csrp3	-5,89	0,00	*Adamts*	1,57	0,00	*Rpl21-p*	0,91	0,00	*Pag1*	1,34	0,02
Klhl40	-7,77	0,00	*Ntm*	2,72	0,00	*Pdha1*	-0,79	0,00	*Car1*	3,47	0,02
Tnfrsf13b	3,75	0,00	*Apol6*	1,62	0,00	*Txlng*	-0,73	0,04	*Trim55*	-5,35	0,00
Ndrg2	-2,21	0,00	*Tfcp2l1*	-2,34	0,00	*Car5b*	-1,06	0,01	*Rpl23a-ps5*	1,73	0,04
Tmtc1	-2,55	0,00	*Cavin3*	1,60	0,00	*Tmsb4x*	0,80	0,00	*Slc7a11*	3,33	0,04
Ppp1r3c	-2,31	0,00	*Pgr*	-3,19	0,00	*Fabp5*	-0,75	0,00	*Foxo1*	0,92	0,00
Dock10	1,94	0,00	*Cdcp1*	-2,63	0,00	*Mccc1*	-0,88	0,01	*Pfn2*	-1,91	0,01
Ptprc	2,78	0,00	*Mtss1*	-0,62	0,00	*Ccdc14*	-3,81	0,02	*Selenot*	-0,53	0,04
Fam46c	3,64	0,00	*Ccdc80*	1,46	0,00	*Cc02*	1,94	0,05	*P2ry13*	2,27	0,04
Igkv4-72	5,32	0,00	*Col7a1*	-3,29	0,00	*Rps23-p*	0,59	0,01	*Schip1*	-2,59	0,02
Slc25a4	-1,62	0,00	*Plekhh1*	-2,36	0,00	*Rpl21-p*	-1,82	0,04	*Trim59*	1,57	0,03
Dock2	2,38	0,00	*Plin4*	1,60	0,00	*Pgrmc2*	-0,76	0,01	*Ppm1l*	-1,75	0,01
Unc45b	-1,90	0,00	*Serinc2*	-2,74	0,00	*Exosc8*	0,75	0,05	*Tmem131l*	1,04	0,04
Snrnp48	1,00	0,00	*Rpsa-ps*	-1,02	0,00	*Rpl13-p*	1,04	0,01	*Fcrl5*	5,06	0,02
Tnfrsf13c	4,57	0,00	*Prune2*	-1,41	0,00	*P2ry13*	1,96	0,02	*Etv3*	0,85	0,03
Mup13	-1,73	0,00	*Cyp2e1*	1,25	0,00	*Mme*	-0,89	0,01	*Sh2d2a*	5,22	0,00
Actc1	-5,10	0,00	*Slc25a1*	-1,19	0,00	*Smc4*	0,98	0,01	*Fam189b*	1,65	0,02
Mrps28	-0,98	0,00	*Lrg1*	1,39	0,00	*Trim59*	1,42	0,02	*Slc50a1*	0,66	0,04
Sipa1	1,89	0,00	*Mgl2*	1,30	0,00	*Ppm1l*	-1,16	0,00	*Pmvk*	-1,00	0,01
Hacd1	-2,17	0,00	*Ap1m2*	-2,65	0,00	*Bche*	-0,94	0,02	*Ube2q1*	0,56	0,04
Fnbp1	1,06	0,00	*Ndrg1*	0,91	0,00	*Rbm46*	-3,21	0,01	*S100a13*	-0,51	0,04
Usp13	-4,60	0,00	*Lims2*	1,48	0,00	*Fgb*	-5,65	0,00	*S100a16*	-0,64	0,02
Hspb7	-5,24	0,00	*Tie1*	0,87	0,00	*Fcrl5*	2,27	0,04	*Sprr2a1*	3,59	0,00
Pou2f2	3,35	0,00	*Slc1a3*	1,18	0,00	*Sh2d2a*	1,54	0,03	*Sprr2a3*	3,96	0,00
Plcg2	2,55	0,00	*Prlr*	-2,32	0,00	*Tmem7*	-0,97	0,00	*Sprr1a*	5,07	0,01
Pnn	0,68	0,00	*Snca*	2,26	0,00	*Fam189*	1,68	0,01	*Rplp0-ps1*	1,09	0,04
Basp1	0,93	0,00	*Irf6*	-2,51	0,00	*Pmvk*	-0,61	0,01	*Rorc*	-1,24	0,03
Rock1	0,74	0,00	*Cdh11*	-1,85	0,00	*Tpm3*	0,79	0,01	*Riiad1*	-3,21	0,01
Ptprcap	3,92	0,00	*Enho*	-1,80	0,00	*S100a1*	3,14	0,01	*Rpl31-ps11*	1,26	0,01
Blnk	2,87	0,00	*Mup13*	-2,16	0,00	*Sprr1a*	5,19	0,00	*Psmb4*	-0,52	0,02
Wnk2	-1,97	0,00	*Bcat2*	0,95	0,00	*Rorc*	-0,77	0,04	*Tnfaip8l2*	1,64	0,02
Cst6	-4,35	0,00	*Sgms2*	-1,45	0,00	*Tnfaip8*	1,51	0,03	*Ctss*	1,26	0,02
Syk	2,69	0,00	*Fam126*	0,70	0,00	*Ctss*	1,64	0,00	*Rps10-ps1*	0,79	0,03
Rassf5	2,41	0,00	*Ackr3*	1,47	0,00	*Mcl1*	0,57	0,02	*Car14*	-3,89	0,01
Igkv4-80	6,57	0,00	*Lypd3*	-4,14	0,00	*Phgdh*	0,95	0,02	*Prkab2*	-1,77	0,01
Adrm1	-0,68	0,00	*Prc1*	-2,84	0,00	*Slc16a1*	-0,85	0,02	*Hmgcs2*	1,17	0,03
Jakmip1	3,64	0,00	*Scg3*	-2,29	0,00	*Dennd2*	0,86	0,01	*Tbx15*	-1,63	0,03
Ubxn1	-0,64	0,00	*Anxa1*	0,93	0,00	*Cd53*	1,43	0,02	*Fam46c*	2,00	0,00
Plcb2	2,57	0,00	*Pf4*	1,53	0,00	*A93000*	0,83	0,03	*Cd2*	2,91	0,04
Foxd2os	2,48	0,00	*Arg2*	-2,90	0,00	*Dbt*	-0,64	0,05	*Csde1*	-0,67	0,03
Snn	1,81	0,00	*Syp*	1,58	0,00	*Agl*	-0,73	0,02	*Ptpn22*	2,41	0,01
Btla	4,28	0,00	*Msi2*	-0,71	0,00	*Dpyd*	-1,48	0,01	*Slc16a1*	-0,92	0,01
Cd72	2,97	0,00	*Slco2a1*	-1,61	0,00	*Abcd3*	-1,00	0,00	*Atp5f1*	-0,72	0,02
Tacc2	-1,79	0,00	*Pid1*	1,03	0,00	*Enpep*	-1,67	0,00	*Dennd2d*	1,21	0,03
Mthfd1l	3,30	0,00	*Mccc2*	1,14	0,00	*Elovl6*	-1,23	0,00	*Cd53*	2,04	0,00
Rac2	3,08	0,00	*Kit*	-1,45	0,00	*Pla2g12*	-0,79	0,03	*AI504432*	2,38	0,05
Tnfsf8	4,56	0,00	*Aoc1*	-5,18	0,00	*Mcub*	1,45	0,03	*Vcam1*	1,88	0,00

Table S9: GO annotation revealed common biological processes involved in adipose tissue of PNA, Peripubertal androgenized , 17NF, and maternal obesity animal models

PNA				Peripubertal androgenized				17NF				Maternal obesity			
ID	Term	p-value	adjust.p	ID	Term	p-value	adjust.p	ID	Term	p-value	adjust.p	ID	Term	p-value	adjust.p
GO:0008654	Lipid metabolism	0,00	0,00	GO:0006631	Lipid metabolism	1,39E-11	1,39E-11	GO:0019216	Lipid metabolism	6,02E-10	6,02E-10	GO:0010876	Lipid metabolism	0,03	0,03
GO:0034109	Regulation of cell-cell adhesion	0,00	0,00	GO:0022407	Regulation of cell-cell adhesion	4,11E-07	4,11E-07	GO:0022408	Regulation of cell-cell adhesion	3,54E-09	3,54E-09	GO:0010906	Glucose metabolic process	0,04	0,04
GO:0006006	Glucose metabolic process	0,00	0,00	GO:0006006	Glucose metabolic process	5,07E-04	5,07E-04	GO:0006006	Glucose metabolic process	1,33E-06	1,33E-06	GO:0022409	Regulation of cell-cell adhesion	1,02E-20	1,02E-20
GO:0061179	Response to insulin	0,01	0,01	GO:0032868	Response to insulin	5,19E-07	5,19E-07	GO:0050796	Response to insulin	5,54E-06	5,54E-06	GO:0046676	Response to insulin	0,00	0,00
GO:0033145	Hormone metabolic process	0,03	0,03	GO:0042445	Hormone metabolic process	6,26E-07	6,26E-07	GO:0043434	Response to peptide hormone	0,00	0,00	GO:0090278	Response to peptide hormone	0,00	0,00
GO:0002263	Regulation of inflammatory response	1,15E-17	1,15E-17	GO:0060612	Adipose tissue development	8,87E-04	8,87E-04	GO:0046883	Hormone metabolic process	0,00	0,00	GO:0046888	Hormone metabolic process	0,03	0,03
GO:0070371	ERK1 and ERK2 cascade	1,81E-06	1,81E-06	GO:0070372	ERK1 and ERK2 cascade	7,72E-05	7,72E-05	GO:0034599	Response to oxidative stress	0,05	0,05	GO:0006979	Response to oxidative stress	0,00	0,00
GO:2001233	Regulation of apoptotic signaling pathway	2,78E-06	2,78E-06	GO:0006979	Response to oxidative stress	1,28E-05	1,28E-05	GO:0032735	Regulation of inflammatory response	0,00	0,00	GO:0050728	Regulation of inflammatory response	0,00	0,00
GO:0034599	Response to oxidative stress	0,01	0,01	GO:0050727	Regulation of inflammatory response	5,22E-08	5,22E-08	GO:0032868	Response to insulin	0,00	0,00	GO:0070371	ERK1 and ERK2 cascade	0,00	0,00
GO:0009166	Nucleotide metabolism	0,00	0,00	GO:0060612	Adipose tissue development	8,87E-04	8,87E-04	GO:2001235	Regulation of apoptotic signaling pathway	0,02	0,02	GO:2001233	Regulation of apoptotic signaling pathway	0,00	0,00
GO:0008654	Response to T	0,02	0,00	GO:2001233	Regulation of apoptotic signaling pathway	4,07E-06	4,07E-06	GO:0051153	Regulation of muscle development	0,04	0,04	GO:0051147	Regulation of muscle development	0,00	0,00
GO:0048634	Regulation of muscle development	0,00	0,00	GO:0051150	Regulation of muscle development	1,05E-05	1,05E-05	GO:0070371	ERK1 and ERK2 cascade	0,00	0,00	GO:0045980	Nucleotide metabolism	0,03	0,03
				GO:0043434	Response to peptide hormone	5,73E-07	5,73E-07	GO:0060612	Adipose tissue development	0,04	0,04	GO:0033574	Response to T	0,04	0,04
				GO:0019433	TCA	1,73E-02	3,92E-02	GO:0072350	TCA	1,10E-09	1,10E-09				
				GO:0033574	Response to T	8,34E-03	8,34E-03	GO:0045980	Nucleotide metabolism	0,04	0,05				

Table S10: Common genes between PNA, Peripubertal androgenized, 17NF, and maternal obesity models and PCOS patients

Common genes between PNA model and PCOS patients		
up regulated		
Human Ge	Fold Change in PNA	Fold change in PCOS women
C19orf38	2,40	1,12
B3GNT5	4,08	0,38
BANK1	3,17	0,32
BCL2A1	2,40	1,29
BCL2L11	1,02	0,26
BIN2	3,08	0,67
BTK	3,12	0,48
CCL22	2,81	1,77
CCL22	2,81	1,16
CCR7	3,11	1,02
CD52	2,84	0,76
CD72	2,97	0,63
CIITA	3,02	0,30
CIITA	3,02	0,61
CORO1A	2,74	0,61
CRLF3	1,93	0,26
CTSW	2,84	0,63
CYTIP	1,63	0,96
DOCK10	1,94	0,37
DOCK8	1,88	0,29
ELF4	2,45	0,35
EML3	0,76	0,22
FGD3	3,12	0,61
FGR	2,74	0,86
FMNL1	2,07	0,43
FMNL3	1,19	0,23
GSAP	2,23	0,27
GXYLT1	0,89	0,23
HELZ2	1,86	0,40
IL2RG	2,63	0,78
IL7R	3,43	0,85
IPCEF1	3,39	1,09
ITGA4	1,64	0,63
ITGB2	1,62	0,54
ITK	4,06	1,05
KLHL6	2,63	0,59
KMT2A	0,93	0,27
LCP1	2,00	0,39
LEF1	3,58	1,10
LY75	2,73	0,46
LY75-CD30	2,73	0,46
LYST	0,92	0,18
MAP3K1	1,14	0,21
MATK	4,06	1,00
MATK	4,06	0,90
MCTP2	2,49	0,96
MICAL1	1,80	0,26
MIER1	0,72	0,20
MS4A1	4,51	1,54
MS4A6E	1,56	3,09
MYO1F	1,85	0,62
NCF2	1,17	0,54
NFATC1	1,48	0,56
NFKB2	1,32	0,28
PARP8	1,43	0,37
PARVG	3,10	0,60
PIK3R5	1,87	0,67
PIP5K1C	1,27	0,35
PLAUR	1,60	0,57
PRKCB	2,27	0,80
PSD4	2,69	0,63
PTPN22	2,55	0,72
PTPRC	2,78	0,48
RAC2	3,08	0,80
RASSF5	2,41	0,65
RPS6KA1	1,27	0,62
RUNX3	3,10	0,85
RUNX3	3,10	0,55
SCML4	1,69	1,29
SFI1	1,22	0,45
SH3BP2	1,36	0,18
SH3BP2	1,36	0,17
SIPA1	1,89	0,29
SLAMF7	2,87	1,08
SLC16A6	1,91	1,12
SLC7A1	1,23	0,56
SLC7A11	3,49	0,99
SMAP2	1,51	0,28
SP140	1,38	1,03
SPAG5	1,42	0,46
STK4	1,50	0,20
TRAF3IP3	2,94	0,75
TRERF1	0,95	0,32
UAP1L1	1,01	0,29
WDFY4	2,61	0,53
down regulated		
ATP6V1G1	-0,92	-0,33
CHCHD10	-1,27	-0,33
DNLZ	-0,88	-0,49
FILIP1	-1,48	-0,56
GPR180	-0,72	-0,34
ITM2A	-1,06	-0,44
KLHL31	-8,77	-0,56
NDUFA12	-1,01	-0,29
PI16	-1,08	-0,70
TTLL7	-3,66	-0,50
UBC	-0,62	-0,50

Common genes between Peripubertal androgenized model and PCOS patients		
up regulated		
Human Gene	Fold Change in Peripubertal androgenized	Fold change in PCOS women
AOAH	0,99	0,66
AOX1	1,10	0,46
APOBR	0,93	1,27
ARL4A	0,92	0,32
KIAA1614	1,59	0,58
BEAN1	3,94	0,48
BMPER	1,28	0,46
BTRC	0,61	0,22
C5AR1	1,51	0,81
CATSPERG	1,86	0,88
CELF2	0,39	0,16
CLDN15	1,21	0,41
CNR1	1,90	0,53
COL4A4	3,22	0,75
CPEB4	0,79	0,31
CYP27A1	0,67	0,38
EHBP1L1	0,47	0,20
FAM189A2	1,05	0,50
GAA	0,49	0,40
HP	2,21	0,52
MNDA	0,99	0,69
IL1R2	2,32	1,07
MAP3K6	1,35	0,30
MOCOS	1,04	0,60
MOCOS	1,04	0,64
MS4A6E	1,41	3,09
MTHFR	0,92	0,25
PCSK6	1,55	0,63
PLA2G7	0,81	0,57
PPL	1,26	0,33
PRX	2,34	0,52
RAB11FIP5	0,52	0,31
RUSC2	1,00	0,19
SERPINE1	2,60	0,84
SIGLEC9	1,61	0,95
SIGLEC7	1,61	1,14
SYT12	1,17	0,71
TBC1D22A	0,66	0,23
TLR8	2,29	0,92
down regulated		
ACLY	-2,03	-0,40
ACSL3	-1,21	-0,32
ADCK5	-1,16	-0,35
AGTR1	-1,48	-0,36
CCT6A	-0,37	-0,23
CCT8	-0,29	-0,25
CHCHD10	-1,04	-0,33
CNN3	-0,65	-0,36
CRISPLD1	-1,49	-1,17
CRISPLD1	-1,49	-0,68
DCLK1	-0,93	-0,30
DLAT	-0,76	-0,48
ELOVL5	-0,51	-0,48
FADS2	-1,30	-0,75
HNRNPA1	-0,80	-0,39
HSPA9	-0,42	-0,33
LUM	-0,81	-0,48
ME1	-1,78	-0,39
PDHB	-0,98	-0,23
1,00 PGK	-0,64	-0,31
PLK2	-1,16	-0,27
PLOD2	-0,79	-0,40
POSTN	-1,57	-0,35
PPP1R1B	-3,31	-0,50
PYGL	-1,30	-0,39
RBM12	-0,82	-0,25
RGS2	-0,99	-0,38
SETX	-0,62	-0,16
SFPQ	-0,64	-0,24
SMCHD1	-0,63	-0,23
THSD4	-1,36	-0,73
THY1	-1,04	-0,19

Common genes between 17NF model and PCOS patients		
up regulated		
Human Gene	Fold Cha	Fold change in PCOS women
C19orf38	1,51	1,12
BCL2A1	1,63	1,29
BIN2	2,18	0,67
BTK	1,37	0,48
CCR5	1,19	0,91
CENPE	1,91	0,51
CIITA	1,95	0,30
CIITA	1,95	0,61
CLEC7A	2,33	0,81
DOCK10	1,28	0,37
DOCK8	1,02	0,29
ELF4	1,32	0,35
EVI2A	1,58	0,52
FAM189B	1,68	0,64
FGD3	1,52	0,61
FGR	1,95	0,86
FMNL1	1,52	0,43
GLIPR2	0,99	0,31
HELZ2	1,24	0,40
MNDA	0,84	0,69
IL2RB	1,87	0,72
IRF7	1,91	0,32
ITGA4	1,25	0,63
ITGAX	1,52	1,16
ITGAX	1,52	0,44
ITGB2	1,53	0,54
JADE2	0,85	0,27
KLHL6	1,64	0,59
LCP1	1,26	0,39
LRRK1	0,98	0,35
LST1	1,31	0,62
MS4A6E	1,50	3,09
MYO1F	1,05	0,62
NCF2	1,08	0,54
PADI2	1,76	1,33
PARP8	1,76	0,37
PARVG	1,96	0,60
PIK3R5	1,67	0,67
LILRA5	2,17	1,17
PLXNC1	1,66	0,72
PRF1	2,25	0,60
PRKCB	1,40	0,80
PSD4	1,95	0,63
PTP4A3	1,18	0,30
RGS1	2,23	1,42
RUNX3	1,89	0,85
RUNX3	1,89	0,55
SEMA4D	1,72	0,71
SH3BP2	1,40	0,18
SH3BP2	1,40	0,17
SIPA1	0,82	0,29
SLAMF7	2,12	1,08
SMAP2	0,94	0,28
SP140	0,97	1,03
SRPK3	2,88	0,87
down regulated		
ACADSB	-1,03	-0,23
ACLY	-1,22	-0,40
ACSL1	-0,71	-0,55
AGT	-2,95	-0,85
ALDH4A1	-1,34	-0,24
ARHGAP18	-0,69	-0,31
CEBPB	-1,13	-0,26
CEP70	-1,23	-0,37
CHCHD10	-0,87	-0,33
CHRDL1	-1,24	-0,45
COQ9	-0,72	-0,22
CYB5B	-0,96	-0,26
DDX3X	-0,47	-0,30
DGAT2	-1,18	-0,62
DLAT	-1,18	-0,48
DLD	-0,87	-0,34
ECHDC1	-1,52	-0,35
ELOVL3	-2,50	-0,58
ELOVL5	-1,77	-0,48
GLO1	-0,94	-0,25
GLUL	-0,77	-0,53
GPAM	-1,37	-0,56
GPT	-0,79	-0,33
HOXA9	-1,20	-0,38
HSPA8	-0,37	-0,45
HSPA9	-0,64	-0,33
ISCA1	-0,70	-0,29
LRRC58	-0,48	-0,27
MDH1	-0,86	-0,50
ME1	-1,00	-0,39
MGP	-2,41	-0,47
MGST1	-0,92	-0,45
MIB1	-0,75	-0,20
MMD	-0,84	-0,49
MME	-0,89	-0,26
MME	-0,89	-0,58
NCOA4	-1,45	-0,41
NDUFS1	-0,61	-0,31
NEGR1	-0,79	-0,30
ORMDL3	-0,73	-0,31
ORMDL3	-0,73	-0,38
PDHB	-0,92	-0,23
PGRMC1	-0,78	-0,31
PLA2G6	-0,95	-0,30
PLA2R1	-1,14	-0,21
PPA1	-1,07	-0,35
PRDX3	-1,01	-0,46
PYGL	-0,94	-0,39
RIOK3	-0,64	-0,15
SCD	-0,86	-0,58
SDHD	-0,60	-0,39
SLC25A16	-1,08	-0,39
SLC25A51	-0,64	-0,20
SLC25A51	-0,64	-0,33
SREBF1	-1,03	-0,58
ST13	-0,56	-0,37
THRSP	-0,95	-0,42
TMEM135	-0,77	-0,37
UBC	-0,70	-0,50
UGP2	-0,96	-0,50
UQCRC2	-0,57	-0,35
USP33	-0,74	-0,33
VEGFA	-0,76	-0,37
ZFAND6	-0,81	-0,34

Common genes between maternal obesity model and PCOS patients		
up regulated		
Human Gene	Fold Change in maternal obesity	Fold change in PCOS women
C19orf38	2,31	1,12
ARHGAP22	2,36	0,71
B3GNT5	3,57	0,38
B4GALNT1	1,77	0,89
BANK1	2,69	0,32
BCL2A1	2,18	1,29
BCL2L11	1,00	0,26
BIN2	3,27	0,67
BTK	2,70	0,48
CBX7	1,21	0,41
CCL22	3,33	1,77
CCL22	3,33	1,16
CCR7	3,32	1,02
CD22	3,97	0,80
CD52	2,68	0,76
CD69	2,47	0,74
CD72	2,51	0,63
CIITA	2,40	0,30
CIITA	2,40	0,61
CORO1A	2,49	0,61
CRLF3	1,91	0,26
CTSW	3,32	0,63
CXCR4	1,73	0,67
CYTIP	1,57	0,96
DOCK10	1,96	0,37
DOCK8	1,72	0,29
ELF4	2,00	0,35
FAM189B	1,65	0,64
FMNL1	2,32	0,43
FRMD8	0,92	0,23
GLIPR2	1,22	0,31
HELZ2	1,18	0,40
IFT140	1,14	0,37
IL2RB	2,56	0,72
IL2RG	2,64	0,78
IL7R	4,05	0,85
IPCEF1	3,73	1,09
ITGA4	1,59	0,63
ITGB2	1,45	0,54
ITK	4,31	1,05
KLHL6	1,88	0,59
LCP1	1,54	0,39
LEF1	3,79	1,10
LRRC45	1,04	0,39
LY75	2,50	0,46
LY75-CD302	2,50	0,46
LYST	0,79	0,18
MATK	4,22	1,00
MATK	4,22	0,90
MCTP2	3,00	0,96
MICAL1	1,80	0,26
MS4A6E	1,51	3,09
MYO1F	1,52	0,62
MYO9B	0,93	0,18
NFATC1	1,11	0,56
NFKB2	1,26	0,28
PARVG	2,30	0,60
PIK3R5	1,68	0,67
PIP5K1C	1,06	0,35
PRF1	2,99	0,60
PRKCB	1,79	0,80
PSD4	2,61	0,63
PTPN22	2,41	0,72
PTPRC	2,53	0,48
RAC2	2,87	0,80
RASSF5	2,37	0,65
RUNX3	2,44	0,85
RUNX3	2,44	0,55
SCML4	1,72	1,29
SIPA1	1,84	0,29
SLAMF7	1,72	1,08
SLC7A11	3,33	0,99
SMAP2	1,49	0,28
STK4	1,15	0,20
TRAF3IP3	2,77	0,75
UNC13D	2,03	0,48
WDFY4	2,04	0,53
down regulated		
ACLY	-1,24	-0,40
ACTA2	-0,49	-0,32
ATP6V1G1	-0,97	-0,33
CHCHD2	-0,73	-0,33
CISH	-1,16	-0,83
ELOVL3	-2,55	-0,58
ELOVL5	-0,52	-0,48
FILIP1	-1,72	-0,56
HINT1	-0,54	-0,30
HOXA9	-1,63	-0,38
IDI1	-1,08	-0,32
ME1	-1,07	-0,39
MRPL50	-0,86	-0,32
NDUFA12	-0,74	-0,29
NDUFA5	-0,73	-0,45
1,00 PGK	-1,04	-0,31
PI16	-0,50	-0,70
PRSS23	-0,92	-0,35
RCAN2	-2,32	-0,56
RCAN2	-2,32	-0,57
TTLL7	-2,20	-0,50
UBC	-0,60	-0,50
UGP2	-1,12	-0,50

Table S11: Common and uniquens upregulated and and downregulated genes across different animal models and different target tissues and cell

Models	Hypothalamus	Ovary	MII oocytes	Adipose Tissue
**PNA**				
	Atp1b1 ↓			*Atp2a3* ↓
	Ccl21a ↑	*Ccl21a* ↓		*Ccl5* ↑
	Rpl10-ps6 ↑			*Rpl3l* ↓
		Wfdc18 ↑		*Wfdc1* ↓
**Maternal Obesity**				
		Atp1a3 ↑		*Atp1a3* ↑
		Ccl4 ↑		*Ccl5* ↑
		Chchd2-ps ↑		*Chchd2-ps* ↓
		Elovl6 ↓		*Elovl5* ↓
		Fcer1a ↓		*Fcer2a* ↑
		Hspb1 ↑		*Hspb1* ↓
		Ms4a4c ↑		*Ms4a6b* ↑
		Rpl5-ps2 ↓		*Rpl5-ps1* ↑
**Peripubeteral androgenized**				
	Atp5j ↑	*Atp1a3* ↓		*Atp1a3* ↓
		C1qa ↑		*C1qa* ↑
		C1qb ↑		*C1qb* ↑
		C1qc ↑		*C1qc* ↑
		Ccl6 ↑		*Ccl6* ↑
		Cfp ↑		*Cfp* ↑
		Chchd2-ps ↓		*Chchd2-ps* ↑
		Csf1r ↑		*Csf1r* ↑
		Elovl5 ↓		*Elovl5* ↓
		Fcer1g ↑		*Fcer1g* ↑
		Hspb1 ↓		*Hspb1* ↑
		Ms4a7 ↑		*Ms4a7* ↑
		Prune2 ↓		*Prune2* ↓
		Rpl3-ps1 ↓		*Rpl3-ps1* ↓
		Tcim ↓		*Tcim* ↑
		Tkt ↓		*Tkt* ↓
	Wfdc18 ↓	*Wfdc17* ↑		*Wfdc17* ↑
**17NF**				
		Atp1a3 ↓		*Atp1a3* ↓
		C1qa ↑		*C1qa* ↑
		C1qb ↑	*C1qb* ↑	*C1qb* ↑
		C1qc ↑		*C1qc* ↑
		Ccl6 ↑		*Ccl6* ↑
		Cfp ↑		*Cfp* ↑
		Chchd2-ps ↓		*Chchd2-ps* ↓
		Csf1r ↑		*Csf1r* ↑
	Elovl6 ↑	*Elovl5* ↓	*Elovl5* ↑	*Elovl5* ↓
		Fcer1g ↑		*Fcer1g* ↑
		Hspb1 ↓	*Hspb1* ↑	*Hspb1* ↓
		Ms4a7 ↑		*Ms4a7* ↑
		Prune2 ↓		*Prune2* ↓
		Rpl3-ps1 ↓		*Rpl3-ps1* ↓
		Tcim ↓		*Tcim* ↓
		Tkt ↓		*Tkt* ↓
		Wfdc17 ↑		*Wfdc17* ↑

Table S12: Steriod hormone metabolism-regulating panel of genes in target tissues and MII oocytes in PNA, Peripubertal androgenized , 17NF, and maternal obesity animal models

Models	Hypothalamus	Ovary	MII oocytes	Adipose Tissue
**PNA**	*Hsd3b1* ↑	*Hsd11b2* ↓	*Hsd3b1* ↑	
**Maternal Obesity**	*Hsd3b1* ↓	*Hsd11b2* ↓	*Hsd3b1* ↑	
**Peripubeteral androgenized**		*Hsd17b7* ↓<br>*Hsd3b1* ↓	*Hsd17b12* ↑	*Hsd17b10* ↑
**17NF**		*Hsd11b1* ↓<br>*Hsd17b12* ↓<br>*Hsd17b7* ↓<br>*Hsd3b1* ↓	*Hsd17b13* ↑	*Hsd11b1* ↓<br>*Hsd17b12* ↓<br>*Hsd17b7* ↓

Table S13: A unique gene influenced by hyperandrogenism and exposure to maternal obesity n target tissues and MII oocytes in PNA, Peripubertal androgenized, 17NF, and maternal obesity animal models

Models	Hypothalamus	Ovary	MII oocytes	Adipose Tissue
**PNA**	*Cfd* ↓<br>*Cyp11a1* ↑<br>*Fabp4* ↓<br>*Fam131a* ↓<br>*Scd1* ↓	*Car14* ↓<br>*Cyp2f2* ↑<br>*Fabp4* ↑<br>*Fam213b* ↓<br>*Scd4* ↑	*Cyp11a1* ↓<br>*Fam221a* ↓	*Cfd* ↓<br>*Fabp3* ↑
**Maternal Obesity**	*Cfd* ↓<br>*Cyp11a1* ↓<br>*Fabp4* ↓<br>*Fam193b* ↑<br>*Scd1* ↓	*Car14* ↓<br>*Cyp11a1* ↑<br>*Cyp19a1* ↓<br>*Fam213b* ↑	*Cyp1b1* ↑<br>*Fam222b* ↓	*Cfd* ↓<br>*Car14* ↓<br>*Cyp27a1* ↓<br>*Scd3* ↓
**Peripubeteral androgenized**	*Fam133b* ↑	*Car3* ↑<br>*Cyp27a1* ↑<br>*Cyp2d22* ↓<br>*Fam213b* ↓	*Cyp2c29* ↓<br>*Fam220a* ↓	*Car3* ↑<br>*Cyp27a1* ↑<br>*Fam213b* ↑
**17NF**	*Scd1* ↑	*Car5b* ↓<br>*Cyp19a1* ↑<br>*Cyp2d22* ↓<br>*Fam213b* ↓	*Cyp19a1* ↑<br>*Fam214b* ↓	*Car5b* ↓<br>*Cfd* ↓<br>*Cyp2d22* ↓<br>*Fabp5* ↓<br>*Fam111a* ↑<br>*Scd2* ↓
