# Supplementary material for: Transcriptomic survey of key reproductive and metabolic tissues in mouse models of polycystic ovary syndrome
Source: Commun Biol. 2023 Jan 18;6:69. doi: 10.1038/s42003-022-04362-0 (PMC9849269; doi:10.1038/s42003-022-04362-0)
Supplement: Supplementary file 2 — Description of additional supplementary files [file 42003_2022_4362_MOESM2_ESM.docx]

**Description of Additional Supplementary**

Files File Name: Supplementary Data 1

Description: The source data for Fig. 2c-f

File Name: Supplementary Data 2

Description: The source data for Fig. 3c

File Name: Supplementary Data 3

Description: The source data for Fig. 5a-d

File Name: Supplementary Data 4

Description: The source data for Fig. 6c
