# Supplementary material for: Transcriptomic survey of key reproductive and metabolic tissues in mouse models of polycystic ovary syndrome
Source: Commun Biol. 2023 Jan 18;6:69. doi: 10.1038/s42003-022-04362-0 (PMC9849269; doi:10.1038/s42003-022-04362-0)
Supplement: Supplementary file 4 — Reporting summary [file 42003_2022_4362_MOESM4_ESM.pdf]

nature portfolio

Corresponding author(s): Qiaolin Deng

Last updated by author(s): Dec 8, 2022

Reporting Summary

Nature Portfolio wishes to improve the reproducibility of the work that we publish. This form provides structure for consistency and transparency in reporting. For further information on Nature Portfolio policies, see our Editorial Policies and the Editorial Policy Checklist.

Statistics

For all statistical analyses, confirm that the following items are present in the figure legend, table legend, main text, or Methods section.

n/a	Confirmed	
☐	☒	The exact sample size (*n*) for each experimental group/condition, given as a discrete number and unit of measurement
☐	☒	A statement on whether measurements were taken from distinct samples or whether the same sample was measured repeatedly
☐	☒	The statistical test(s) used AND whether they are one- or two-sided<br>*Only common tests should be described solely by name; describe more complex techniques in the Methods section.*
☐	☒	A description of all covariates tested
☐	☒	A description of any assumptions or corrections, such as tests of normality and adjustment for multiple comparisons
☐	☒	A full description of the statistical parameters including central tendency (e.g. means) or other basic estimates (e.g. regression coefficient) AND variation (e.g. standard deviation) or associated estimates of uncertainty (e.g. confidence intervals)
☐	☒	For null hypothesis testing, the test statistic (e.g. *F*, *t*, *r*) with confidence intervals, effect sizes, degrees of freedom and *P* value noted<br>*Give P values as exact values whenever suitable.*
☒	☐	For Bayesian analysis, information on the choice of priors and Markov chain Monte Carlo settings
☒	☐	For hierarchical and complex designs, identification of the appropriate level for tests and full reporting of outcomes
☒	☐	Estimates of effect sizes (e.g. Cohen's *d*, Pearson's *r*), indicating how they were calculated

Our web collection on statistics for biologists contains articles on many of the points above.

Software and code

Policy information about availability of computer code

Data collection

The sequencing data can be found in SRA database.

Data analysis

RNA-seq data processing: Raw reads generated by Smart-seq3 protocol were mapped by zUMIs pipeline (Parekh et al., 2018). Raw reads generated by Smart-seq2 protocol were mapped to mouse reference genome (GRCm38/mm10) using STAR default arguments.

DEG analysis and Gene Ontology analysis: DEGs were calculated using DESeq2 method (R package "DEGseq2 of version 1.34.0). DEG genes were defined by p < 0.05 with log2 foldchange > 0.5 or log2 foldchange < -0.5. Gene Ontology analysis of DEGs was performed by 'Clusterprofiler' R package.

WGCNA analyses: Normalized data were performed by 'WGCNA' R package. The power parameter with soft threshold of 9 was selected by 'pickSoftThreshold' function. The Pearson correlation was used in the analyses and the correlation between module eigengenes and different treatment of mice model were calculated to identify modules of interest which were significantly associated with the treatment of mice model.

Cell-Cell communication analyses : To investigate cell-cell communication between MII oocyte and ovary cells, 'CellChat' R package was performed to analysis the ligand and receptor between oocyte and ovary. Ligand-receptor pairs are defined based on 'CellChatDB' database. Based on biological function, all the interactions are grouped into 229 signaling pathway families. The defferentially expressed signaling genes were identified by Wilcoxon rank sum test with significance level of 0.05.

Metabolic pathway analysis of MII oocyte: MII oocytes metabolic pathway quantification was conducted by 'scMetabolism' R package. The

function AUCell was used to quantify the metabolic activity after implement with Seurat pipeline. The genes for pathway analyses can be found online (https://github.com/wu-yc/scMetabolism).

For manuscripts utilizing custom algorithms or software that are central to the research but not yet described in published literature, software must be made available to editors and reviewers. We strongly encourage code deposition in a community repository (e.g. GitHub). See the Nature Portfolio guidelines for submitting code & software for further information.

Data

Policy information about availability of data

All manuscripts must include a data availability statement. This statement should provide the following information, where applicable:

- Accession codes, unique identifiers, or web links for publicly available datasets
- A description of any restrictions on data availability
- For clinical datasets or third party data, please ensure that the statement adheres to our policy

All raw data of bulk and single-cell RNA sequencing data of mouse tissues and MII oocytes are available at SRA database with accession number PRJNA856805. Both will be released on publication.

Human research participants

Policy information about studies involving human research participants and Sex and Gender in Research.

Reporting on sex and gender	N/A
Population characteristics	N/A
Recruitment	N/A
Ethics oversight	N/A

Note that full information on the approval of the study protocol must also be provided in the manuscript.

Field-specific reporting

Please select the one below that is the best fit for your research. If you are not sure, read the appropriate sections before making your selection.

☒ Life sciences ☐ Behavioural & social sciences ☐ Ecological, evolutionary & environmental sciences

For a reference copy of the document with all sections, see nature.com/documents/nr-reporting-summary-flat.pdf

Life sciences study design

All studies must disclose on these points even when the disclosure is negative.

Sample size	Sample size in the mice experiments was based on differences in AGD between CD+vehicle and CD+DHT lineage in our previous studies (see reference Risal S et al., Nature Medicine, 2019).
Data exclusions	No samples or animals were excluded from the analyses unless undetectable value.
Replication	Replicates are noted in the figure legends and experimental findings are reliable reproduced.
Randomization	Mice experiments: As given in Method section, in PNA mice were randomly assigned to be fed control diet or high fat/high sugar diet. During pregnancy, within each diet group were they randomly assigned to be exposed to dihydrotestosterone (DHT) or vehicle at embryonic day 16.5, 17.5 and 18.5.
Blinding	Group allocation during experiments were not blinded to the investigators. However, analyses were repeated by two or more investigators and controlled by an independent statistician not involved in the study. See Statistical Analysis in the Method section.

Reporting for specific materials, systems and methods

We require information from authors about some types of materials, experimental systems and methods used in many studies. Here, indicate whether each material, system or method listed is relevant to your study. If you are not sure if a list item applies to your research, read the appropriate section before selecting a response.

Materials & experimental systems

n/a	Involved in the study	
☒	☐	Antibodies
☒	☐	Eukaryotic cell lines
☒	☐	Palaeontology and archaeology
☐	☒	Animals and other organisms
☒	☐	Clinical data
☒	☐	Dual use research of concern

Methods

n/a	Involved in the study	
☒	☐	ChIP-seq
☒	☐	Flow cytometry
☒	☐	MRI-based neuroimaging

Animals and other research organisms

Policy information about studies involving animals; ARRIVE guidelines recommended for reporting animal research, and Sex and Gender in Research

Laboratory animals	For PNA, peripubertal androgenized, and obese model: Three-week-old female C57Bl/6J mice and eight to twelve weeks old male mice were were obtained from Janvier Labs (Le Genest-Saint-Isle, France).<br><br>For 17NF: The breeding, genetic background, and the generation of the transgenic 17NF mice (MGI Cat# 5662267, RRID: MGI:5662267) has previously described in detail (Dissen et al., 2009; Manti et al., 2020). These mice overexpressed NGF driven by the 17alpha-hydroxylase gene promoter in theca cells of the ovary. The transgene expression in each batch of homozygous 17NF mice was confirmed by genotyping.
Wild animals	The study did not involve wild animals.
Reporting on sex	This include only female mice.
Field-collected samples	The study did not involve samples collected from the field.
Ethics oversight	All animal experiments were approved by the Stockholm Ethical Committee for Animal Research (10798-2017 and 17538-2020) in accordance with the legal requirements of the European Community (SJVFS 2017:40) and the directive 2010/63/EU of the European Parliament on the protection of animals used for scientific purposes. Animal care and procedures were performed in accordance with guidelines specified by European Council Directive and controlled by Comparative Medicine Biomedicum, Karolinska Institutet, Stockholm, Sweden.

Note that full information on the approval of the study protocol must also be provided in the manuscript.
